# ZBTB18 Dysfunction Promotes Neuropathic Pain via CHD4‐based Epigenetic Disinhibition of CLIC1 Channels in Sensory Neurons

**DOI:** 10.1002/advs.77364

**Published:** 2026-08-27

**Authors:** Shoupeng Wang, Zitong Huang, Yu Tao, Yunmei Zhang, Yufang Sun, Dongsheng Jiang, Weiwei Lu, Fuhai Ji, Gang Chen, Min Xu, Yuan Zhang, Jin Tao

**Affiliations:** ^1^ The First Affiliated Hospital of Soochow University School of Basic Medical Sciences Suzhou Medical College of Soochow University Suzhou People's Republic of China; ^2^ Jiangsu Key Laboratory of Drug Discovery and Translational Research For Brain Diseases Centre For Ion Channelopathy Soochow University Suzhou People's Republic of China; ^3^ Precision Research Center For Refractory Diseases Shanghai General Hospital Shanghai Jiao Tong University School of Medicine Shanghai People's Republic of China; ^4^ Department of Anesthesiology & Department of Neurosurgery The First Affiliated Hospital of Soochow University Suzhou People's Republic of China; ^5^ Department of Neurosurgery Kunshan Hospital of Traditional Chinese Medicine Kunshan Affiliated Hospital of Yangzhou University Kunshan People's Republic of China; ^6^ Clinical Research Center of Neurological Disease Department of Geriatrics The Second Affiliated Hospital of Soochow University Suzhou People's Republic of China

**Keywords:** CLIC1, neuronal excitability, neuropathic pain: trigeminal ganglion neurons, ZBTB18

## Abstract

Nerve injury‐induced reprogramming of sensory neuron gene expression is a key driver of neuropathic pain. However, the transcriptional networks that orchestrate this maladaptive plasticity remain largely undefined. Here, we identify the transcriptional repressor ZBTB18 as a critical regulator of this pathogenic process. Peripheral nerve injury markedly downregulated the level of ZBTB18 in the injured trigeminal ganglion (TG) of rats. Restoring ZBTB18 expression reverses injury‐induced mechanical allodynia, while its knockdown in naive TG neurons is sufficient to recapitulate neuropathic pain symptoms. Mechanistically, ZBTB18 directly represses *Clic1* transcription by engaging a specific silencer element within its promoter. This repression is achieved through the recruitment of the nucleosome remodeling and deacetylase (NuRD) complex, an interaction mediated by the ZBTB18 BTB domain and the chromodomain helicase DNA‐binding protein 4 (CHD4). Disruption of this recruitment abrogates histone H3K27ac deacetylation at the *Clic1* promoter, enhancing RNA polymerase II occupancy and driving *Clic1* expression. Consequently, nerve injury‐induced loss of ZBTB18 relieves this epigenetic brake, leading to CLIC1 upregulation, increased chloride channel activity, and hyperexcitability of TG neurons that underlies mechanical hypersensitivity. In summary, these findings reveal a novel ZBTB18/NuRD/CLIC1 epigenetic axis in neuropathic pain and highlight this transcriptional pathway as a potential target for therapeutic intervention.

## Graphical abstract

In this study, we identify a novel functional role of ZBTB18 in regulating trigeminal‐mediated neuropathic pain. Nerve injury reduces ZBTB18 in trigeminal ganglion neurons, impairing CHD4/NuRD recruitment and de‐repressing Clic1. Elevated CLIC1 enhances chloride channel activity and neuronal hyperexcitability, thereby driving pain. Dissection of this ZBTB18–NuRD–CLIC1 axis in nociceptors may unveil new therapeutic targets for neuropathic pain management.

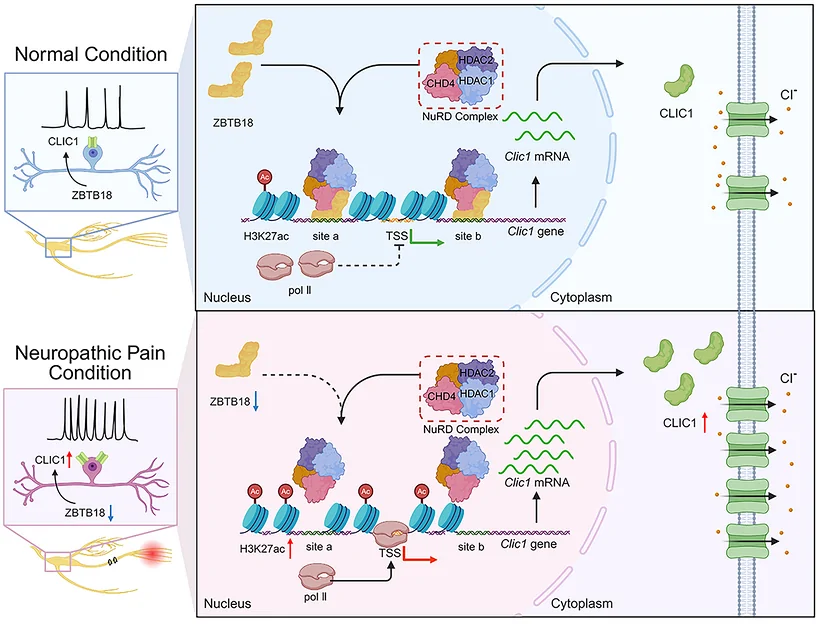

## 1 Introduction

Neuropathic pain, resulting from lesions or diseases of the somatosensory nervous system, is a debilitating chronic condition that severely compromises quality of life [1]. The clinical management of chronic neuropathic pain remains a significant challenge, as existing pharmacotherapies—primarily opioids and nonsteroidal anti‐inflammatory drugs (NSAIDs)—often yield suboptimal analgesia and are associated with considerable adverse effect profiles [2]. Elucidating its molecular pathogenesis is thus a prerequisite for the development of novel therapeutic and prophylactic interventions. Research in primary sensory neurons (e.g., trigeminal ganglion, TG) reveals that peripheral nerve injury can dysregulate pain‐related gene expression at both the transcriptional and translational levels [3, 4]. Such molecular alterations are thought to involve distinct genomic signatures and regulatory processes that contribute to the initiation and maintenance of neuropathic pain [5, 6, 7]. Thus, elucidating how such molecular alterations arise within the TG subsequent to nerve injury may reveal new therapeutic targets for neuropathic pain management.

Nerve injury triggers widespread upregulation of pronociceptive genes in sensory neurons, a process reflecting a loss of transcriptional repressive control [8]. Dysfunction of sequence specific transcriptional repressors represents a critical upstream mechanism underlying this maladaptive transcriptional reprogramming [9]. The zinc finger and BTB domain containing (ZBTB) proteins, which are characterized by an N terminal BTB domain and one or more C terminal C2H2/Krüppel type zinc finger domains [10], form an evolutionarily conserved family of transcription factors particularly well suited to mediate such regulation [11]. This modular architecture enables ZBTB proteins to directly recognize and bind target gene promoters via their zinc finger motifs, while concurrently recruiting epigenetic co repressor complexes—including NuRD and HDAC—through BTB domain mediated protein interactions [12]. Accumulating evidence has linked multiple ZBTB members to neuronal development, synaptic plasticity, and neuroinflammatory regulation [13, 14, 15]. Furthermore, independent transcriptomic analyses have documented differential expression of numerous ZBTB transcripts in sensory ganglia following peripheral nerve injury, highlighting this family as an underappreciated regulatory layer in neuropathic pain pathogenesis [16, 17]. To date, at least 49 ZBTB members have been identified in the human genome, fulfilling diverse roles in both physiological and pathological contexts [18, 19, 20]. Among them, ZBTB18 was initially discovered as a translin‐interacting protein expressed in lymphocytes [21]. It encodes a C2H2‐type zinc finger protein harboring a conserved subclass of POK (POZ and Krüppel)/BTB domains and primarily functions as a transcriptional repressor in various biological processes, including cellular differentiation, development, and metabolism [22, 23, 24, 25]. ZBTB18 has also been implicated in intellectual disability, steatohepatitis, and glioblastoma [26, 27, 28]. In glioblastoma, ZBTB18 is frequently downregulated and functions as a tumor suppressor, whose loss correlates with aggravated malignant phenotypes [28, 29]. Mechanistically, ZBTB18 executes its transcriptional repressive function through interactions with co‐regulatory proteins, including CtBP2, which in turn modulates tumor‐associated transcriptional programs [30, 31]. Moreover, its haploinsufficiency has been associated with corpus callosum dysplasia and impaired glutamatergic synaptic function, ultimately contributing to cognitive deficits [26]. Nonetheless, the potential involvement of ZBTB18 in pain regulation has not yet been elucidated.

In this study, we revealed a pivotal role of ZBTB18 in the development and maintenance of trigeminal neuropathic pain through its regulation of CLIC1 channels. Nerve injury downregulates ZBTB18, which interacts with CHD4 via its BTB domain to recruit the NuRD complex to the *Clic1* promoter. This epigenetic alteration enhances H3K27 acetylation and RNA polymerase II enrichment at the *Clic1* promoter, leading to its transcriptional activation. Restoring ZBTB18 expression in injured TGs abrogated the CCI‐ION‐induced increase in CLIC1 expression and channel currents, thereby suppressing neuronal hyperexcitability and mechanical hypersensitivity. These mechanistic insights identify the ZBTB18‐NuRD‐CLIC1 axis as a potential therapeutic target for neuropathic pain management.

## 2 Results

### 2.1 Nerve Injury Induces ZBTB18 Downregulation in TG Neurons

**FIGURE 1:**
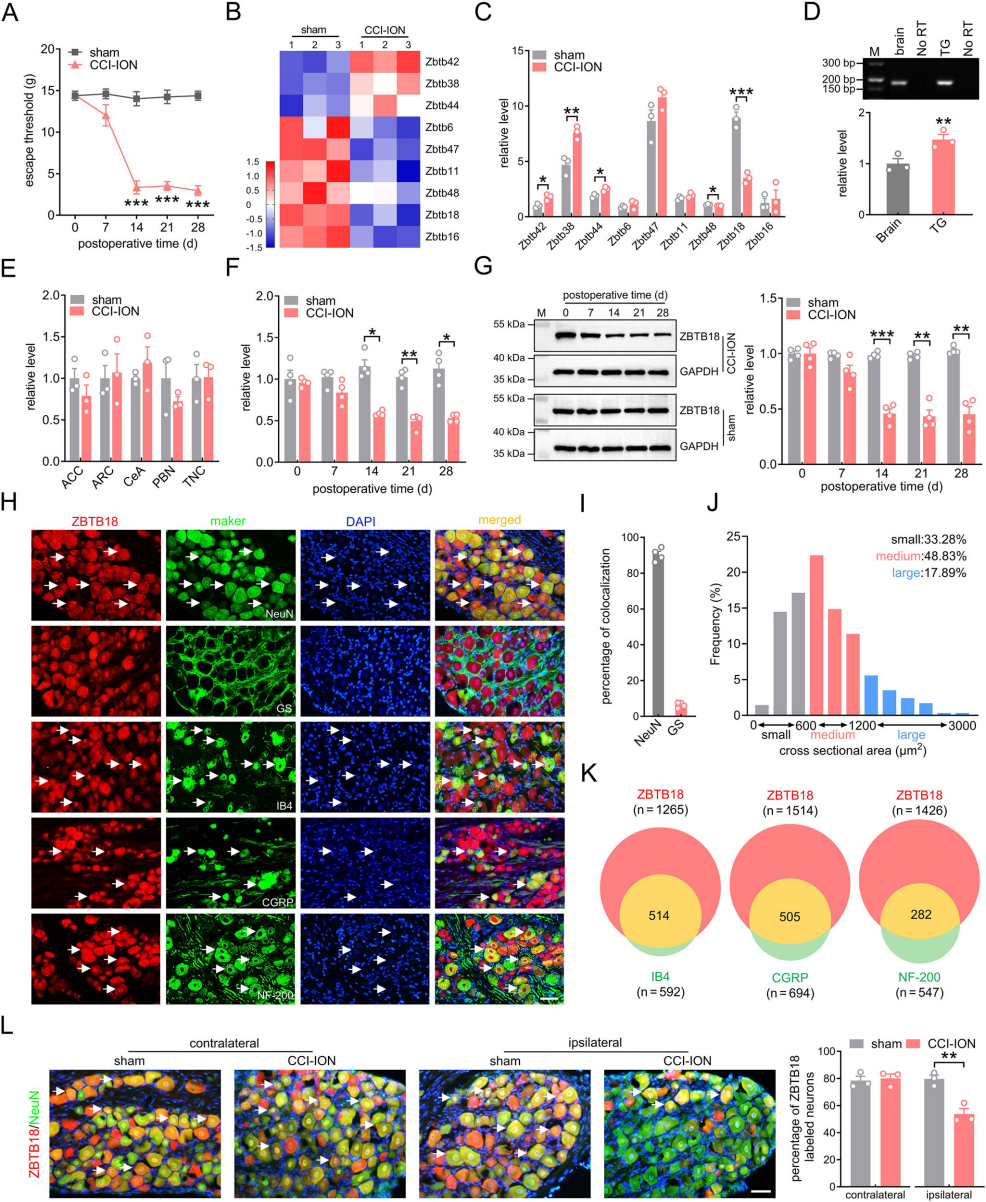
Nerve injury induces ZBTB18 downregulation in TG neurons. (A) Mechanical escape thresholds in response to orofacial stimuli in rats subjected to CCI‐ION or sham surgery. ***p < 0.001 compared with corresponding sham group, two‐way ANOVA, *n* = 6–8 rats/group. (B) Heatmap of RNA‐seq data showing expression changes of 9 ZBTB family genes in ipsilateral TGs on day 14 after CCI‐ION. *n* = 6 rats/group. (C) qPCR validation of the 9 ZBTB family transcripts highlighted in panel *B*. *p < 0.05, **p < 0.01, ***p < 0.001 compared with corresponding sham group, multiple *t*‐test, *n* = 3 rats/group. (D) *Zbtb18* mRNA levels in TGs and brains of intact rats. **p < 0.01 compared with brain group, unpaired *t*‐test, *n* = 3 rats/group. Shown blots are representative of three independent experiments. (E) *Zbtb18* mRNA levels in various brain regions (ACC, ARC, CeA, PBN, TNC) 14 days after CCI‐ION or sham surgery. *p* >0.05 compared with corresponding sham group, multiple *t*‐test, *n* = 3 rats/group. (F) Time‐dependent changes in *Zbtb18* mRNA expression post‐CCI‐ION. *p < 0.05, **p < 0.01 compared with corresponding sham group, two‐way ANOVA, *n* = 4 rats/time point/group. (G) Immunoblot analysis of ZBTB18 protein expression over time. **p < 0.01, ***p < 0.001 compared with corresponding sham group, two‐way ANOVA, *n* = 4 rats/time point/group. Shown blots are representative of four independent experiments. (H) Immunofluorescence co‐localization of ZBTB18 (*red*) with neuronal markers (NeuN, GS, IB4, CGRP, NF200; *green*) in TG neurons of intact rats. Arrows indicate co‐localization. Scale bar, 50 µm. (I) Quantification of ZBTB18‐positive cells coexpressing NeuN or GS. *n* = 4 biological replicates. (J) Proportion of ZBTB18‐positive neurons in intact TGs. *n* = 5 rats. (K) Venn diagram illustrating the overlap of ZBTB18‐positive neurons with IB4‐, CGRP‐, or NF200‐positive subpopulations. *n* = 3–6 rats. (L) Representative images (*left panel*) and quantification (*right panel*) of ZBTB18‐positive neurons in ipsilateral and contralateral TGs 14 days after CCI‐ION or sham surgery. **p < 0.01 compared with corresponding sham group, unpaired *t*‐test, *n* = 3 rats/group. Arrows indicate examples of positive neurons. Scale bar, 50 µm.

Following unilateral chronic constriction injury of the infraorbital nerve (CCI‐ION), a rat model of trigeminal neuropathic pain was well‐established. Mechanical allodynia was confirmed by a significant decrease in the escape threshold on post‐operative day 14 relative to sham‐operated controls, a phenotype that persisted through day 28 (Figure 1A). To systematically characterize the role of BTB domain‐containing zinc finger proteins (ZBTB) and their associated regulatory networks in neuropathic pain pathogenesis, high‐throughput RNA sequencing (RNA‐seq) was performed on ipsilateral TGs 14 days after CCI‐ION (GEO accession number GSE224814). Of the 48 ZBTB transcription factors expressed in the rat TG, nine showed significant differential expression following nerve injury (Figure 1B). Their expression was further validated using qPCR analysis, and *Zbtb18* was the most robustly downregulated in the ipsilateral TG tissue of CCI‐ION rats (Figure 1C). Thus, we chose to analyze the expression profile and functional characteristics of *Zbtb18*. While *Zbtb18* transcripts were also present in the brain, their abundance was significantly lower than that in the TG (Figure 1D). Interestingly, compared with those in the sham‐operated groups, the expression levels of *Zbtb18* in brain areas associated with pain perception, such as the anterior cingulate cortex (ACC), arcuate nucleus (ARC), parabrachial nucleus (PBN), central amygdala (CeA) or trigeminal nucleus caudalis (TNC) remained unaltered, as depicted in Figure 1E. We next determined the temporal dynamics of *Zbtb18* expression in the ipsilateral TG. *Zbtb18* mRNA level was markedly decreased at postoperative day 14 following CCI‐ION, with this suppression persisting through day 28, whereas the sham surgery showed no difference (Figure 1F). This transcriptional downregulation was paralleled at the protein level, where western blot analysis demonstrated a time‐dependent reduction of ZBTB18 protein in the ipsilateral TG of CCI‐ION rats (Figure 1G), while its expression in the contralateral TG remained unchanged throughout the testing period (Figure S1). As anticipated, sham‐operated control animals exhibited stable, baseline levels of ZBTB18 expression in the ipsilateral TG (Figure 1G). We further validated this expression pattern in female rats and observed a consistent time‐dependent downregulation of ZBTB18 at both the mRNA and protein levels in the injured TG (Figure S2). We next employed double‐labeling immunofluorescence to characterize the cellular identity of ZBTB18‐expressing cells. ZBTB18 immunoreactivity was primarily detected in NeuN‐positive neurons, with only faint signal observed in a small subset of glutamine synthetase (GS)‐positive satellite glial cells in the TG (Figure 1H). Quantitative analysis revealed that approximately 90.32% of ZBTB18‐positive TG cells were labeled by NeuN, while only 6.37% co‐localized with GS, corroborating its predominant expression in neuronal populations (Figure 1I). Size frequency analysis revealed that ∼33.28% of ZBTB18‐labeled neurons were small (<600 µm^2^ in area), ∼48.83% were medium (600–1200 µm^2^ in area), and ∼17.89% were large (>1200 µm^2^ in area) (Figure 1H,J). Further characterization of ZBTB18 in peripheral nociceptors revealed its distinct expression across TG neuron subtypes. ZBTB18‐positive neurons showed strong co‐localization with markers of small‐diameter neurons: approximately 40.63% were IB4‐positive (non‐peptidergic), and 33.36% were CGRP‐positive (peptidergic). In contrast, co‐localization with NF200—a marker for large, myelinated Aβ neurons—was significantly lower, at only 19.78% (Figure 1H,K). Moreover, consistent with the transcriptional and protein expression data, double staining assays revealed a significant decrease in the proportion of ZBTB18‐positive neurons in the ipsilateral TG on Day 14 post CCI‐ION compared with the sham group (sham, 80.08 ± 5.41% vs. CCI‐ION, 53.72 ± 7.09%), with no change observed contralaterally (Figure 1L and Figure S3).

We next investigated the mechanism underlying ZBTB18 downregulation after nerve injury. DNA methylation and histone modifications are two major epigenetic regulators of gene expression [32]. MethPrimer analysis identified CpG islands and three BSP target fragments within the *zbtb18* promoter (Figure S4A), but bisulfite sequencing revealed no significant change in promoter methylation after CCI‐ION (Figure S4B). Given our previous findings that CCI‐ION elevates H3K9me2 and H3K27me3 in rat TG [33, 34], we examined whether these histone marks contribute to *zbtb18* repression. ChIP‐PCR across 12 promoter fragments detected H3K9me2 at F4, F7, and F9 (Figure S5A), and H3K27me3 at F3, F6, F8, F9, and F11 (Figure S6A). CCI‐ION did not alter H3K9me2 at any site (Figure S5B), but significantly increased H3K27me3 at F6, F9, and F11 (Figure S6B). Notably, pharmacological inhibition of H3K27me3 with GSK503 upregulated ZBTB18 expression (Figure S7). Collectively, these results indicate that nerve injury‐induced H3K27me3 deposition at the *zbtb18* promoter mediates its transcriptional repression.

### 2.2 ZBTB18 Regulates Neuropathic Pain‐Related Behaviors

**FIGURE 2:**
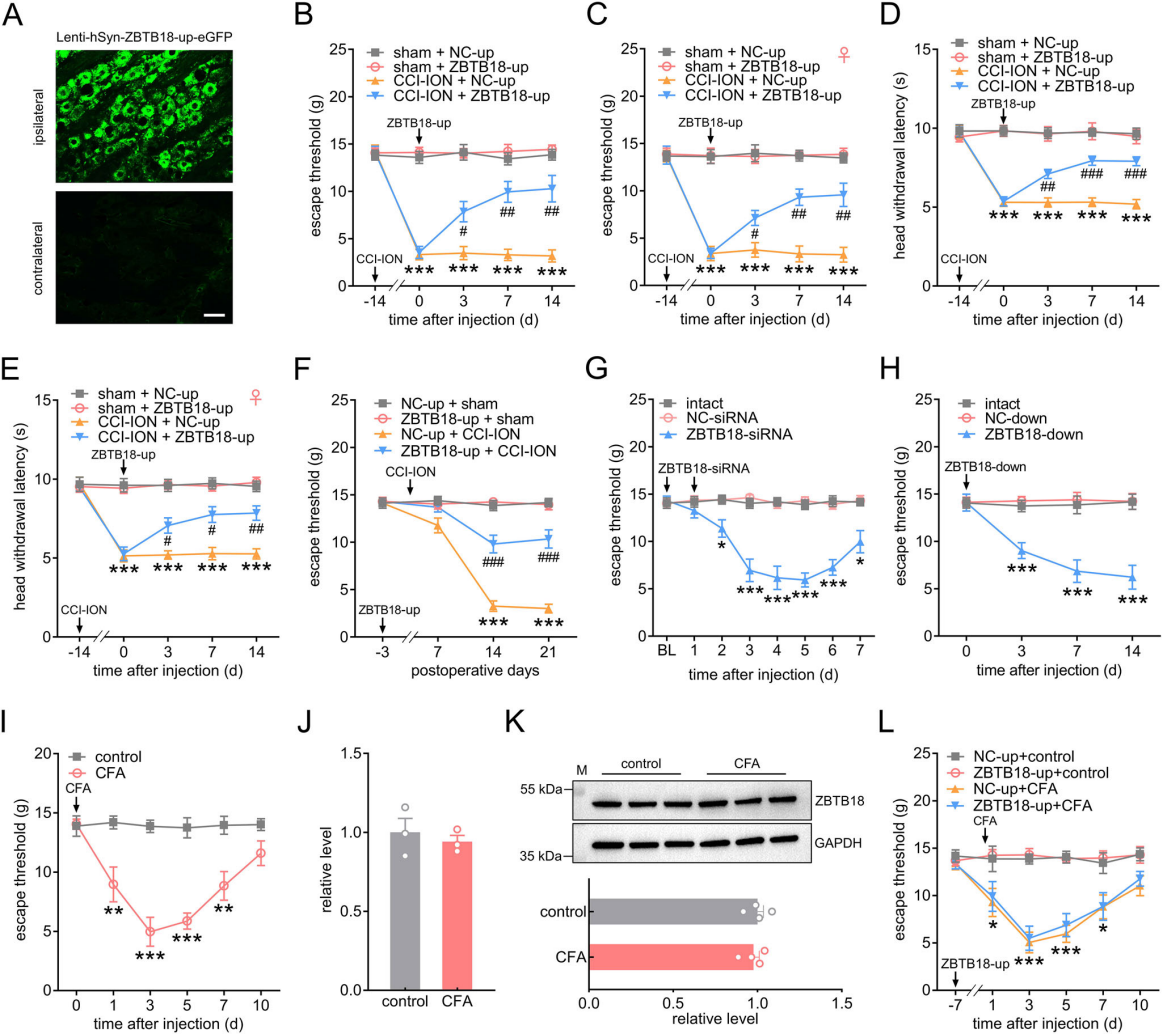
ZBTB18 regulates neuropathic pain‐related behaviors. (A) Representative fluorescence images showing eGFP expression in the ipsilateral and contralateral TG at 7 days after intra‐TG injection of lenti‐hSyn‐ZBTB18‐up (ZBTB18‐up) into the ipsilateral side. Scale bar, 50 µm. (B, C) Effect of ZBTB18‐up on mechanical allodynia in male (*B*) or female (*C*) rats following CCI‐ION or sham surgery. ***p < 0.001 compared with corresponding sham + NC‐up group; ^#^ *p* < 0.05, ^##^ *p* < 0.01 compared with corresponding CCI‐ION + NC‐up group, two‐way ANOVA, *n* = 6‐9 rats/group. (D, E) Intra‐TG injection of ZBTB18‐up ameliorated thermal hyperalgesia in male (*D*) or female (*E*) rats following CCI‐ION. ***p < 0.001 compared with corresponding sham + NC‐up group; ^#^ *p* < 0.05, ^##^ *p* < 0.01, ^###^ *p* < 0.001 compared with corresponding CCI‐ION + NC‐up group, two‐way ANOVA, *n* = 6 rats/group. (F) ZBTB18‐up pretreatment prevented the reduction in mechanical threshold induced by CCI‐ION. ***p < 0.001 compared with corresponding NC‐up + sham group; ^###^ *p* < 0.001 compared with corresponding NC‐up + CCI‐ION group, two‐way ANOVA, *n* = 8 rats/group. (G) ZBTB18‐siRNA induced mechanical hypersensitivity in intact rats. *p < 0.05, ***p < 0.001 compared with corresponding NC‐siRNA group, two‐way ANOVA, *n* = 8 rats/group. (H) ZBTB18‐down induced mechanical hypersensitivity in intact rats. ***p < 0.001 compared with corresponding NC‐down group, two‐way ANOVA, *n* = 8 rats/group. (I) Unilateral buccal pad injection of CFA induced persistent mechanical hypersensitivity. **p < 0.01, ***p < 0.001 compared with corresponding control group, two‐way ANOVA, *n* = 6‐8 rats/group. (J) qPCR analysis showed no significant change in *Zbtb18* mRNA levels following CFA administration. *p* >0.05 compared with control group, unpaired *t*‐test, *n* = 3 rats/group. (K) Immunoblot analysis confirmed no significant change in ZBTB18 protein after CFA treatment. Shown blots are representative of four independent experiments. *p* >0.05 compared with control group, unpaired *t*‐test, *n* = 4 rats/group. (L) ZBTB18‐up failed to reverse CFA‐induced mechanical hypersensitivity. *p < 0.05, ***p < 0.001 compared with corresponding NC‐up + control group, two‐way ANOVA, *n* = 6–8 rats/group.

To determine the functional role of ZBTB18 in trigeminal‐mediated neuropathic pain, we employed a neuron‐specific lentiviral approach to modulate its expression in TG neurons. By using a vector driven by the human synapsin 1 promoter (lenti‐hSyn‐ZBTB18‐up; ZBTB18‐up) and equipped with an eGFP reporter, we observed both lenti‐hSyn‐ZBTB18‐up (ZBTB18‐up) and its negative control vector (NC‐up) drove sustained transgene expression in the ipsilateral TG from day 7 through day 21 postinjection, with no eGFP signal detected in the contralateral TG (Figure 2A & Figure S8), confirming the precision and regional specificity of our intra‐TG delivery. Immunoblot analysis revealed significant ZBTB18 upregulation on day 7 following ZBTB18‐up transduction (Figure S9). Moreover, when ZBTB18‐up was delivered unilaterally intra‐TG on day 14 after CCI‐ION in both male (Figure 2B) and female (Figure 2C) rats, it resulted in a significant attenuation of established mechanical allodynia observed from day 3 to day 14 post ZBTB18‐up transduction. Similarly, the application of ZBTB18‐up markedly alleviated CCI‐ION‐induced heat hyperalgesia in both male (Figure 2D) and female (Figure 2E) rats. Additionally, we evaluated the involvement of ZBTB18 in the development of neuropathic pain by injecting ZBTB18‐up before CCI‐ION. Preemptive overexpression of ZBTB18 produced a significant reduction in the development of mechanical allodynia from days 14 to 21 post‐CCI‐ION compared to rats treated with a negative control vector (NC‐up) (Figure 2F). Subsequently, to determine if the downregulation of ZBTB18 observed after nerve injury is sufficient to modulate pain sensitivity, we first screened four independent ZBTB18‐targeting siRNAs in PC12 cells to identify sequences with high knockdown efficiency. The top two candidates were further validated in vivo via intra‐TG injection, and the siRNA with the most robust in vivo silencing activity, siRNA‐647 (ZBTB18‐siRNA), was selected for all subsequent siRNA‐based experiments (Figure S10A,B). Unilateral delivery of this chemically modified ZBTB18‐siRNA but not NC‐siRNA, into the TG of intact rats significantly reduced ZBTB18 protein abundance (Figure S10C), concomitant with the development of mechanical allodynia (Figure 2G). Moreover, the role of ZBTB18 in pain‐related behaviors was further evaluated following its knockdown with lentivirus‐mediated shRNAs. An eGFP‐labeled lentiviral vector, lenti‐hSyn‐ZBTB18‐down (ZBTB18‐down), was used for neuron‐specific gene delivery. ZBTB18 protein expression was markedly downregulated on day 7 following ZBTB18 knockdown (Figure S10D). Intra‐TG administration of ZBTB18‐down induced robust mechanical allodynia by day 3 and persisted through day 14, whereas NC‐down treatment did not result in any reduction in the escape threshold (Figure 2H). Furthermore, to evaluate the specificity of ZBTB18's role in neuropathic pain, we tested its effect in a model of inflammatory pain—a distinct subchronic pain condition with mechanisms that partially overlap with neuropathic pain [35, 36]. As depicted in Figure 2I, unilateral CFA injection into the buccal pad induced mechanical allodynia, whereas saline treatment did not result in any reduction in the escape threshold. Notably, examination of *ZBTB18* mRNA levels (Figure 2J) and its protein expression (Figure 2K) in the ipsilateral TG tissue showed no significant change following CFA‐induced inflammatory pain. Further, the administration of ZBTB18‐up failed to alleviate the inflammatory pain (Figure 2L), indicating a modality‐specific function for ZBTB18 in neuropathic pain pathogenesis.

### 2.3 CLIC1 Is a Downstream Target of ZBTB18

**FIGURE 3:**
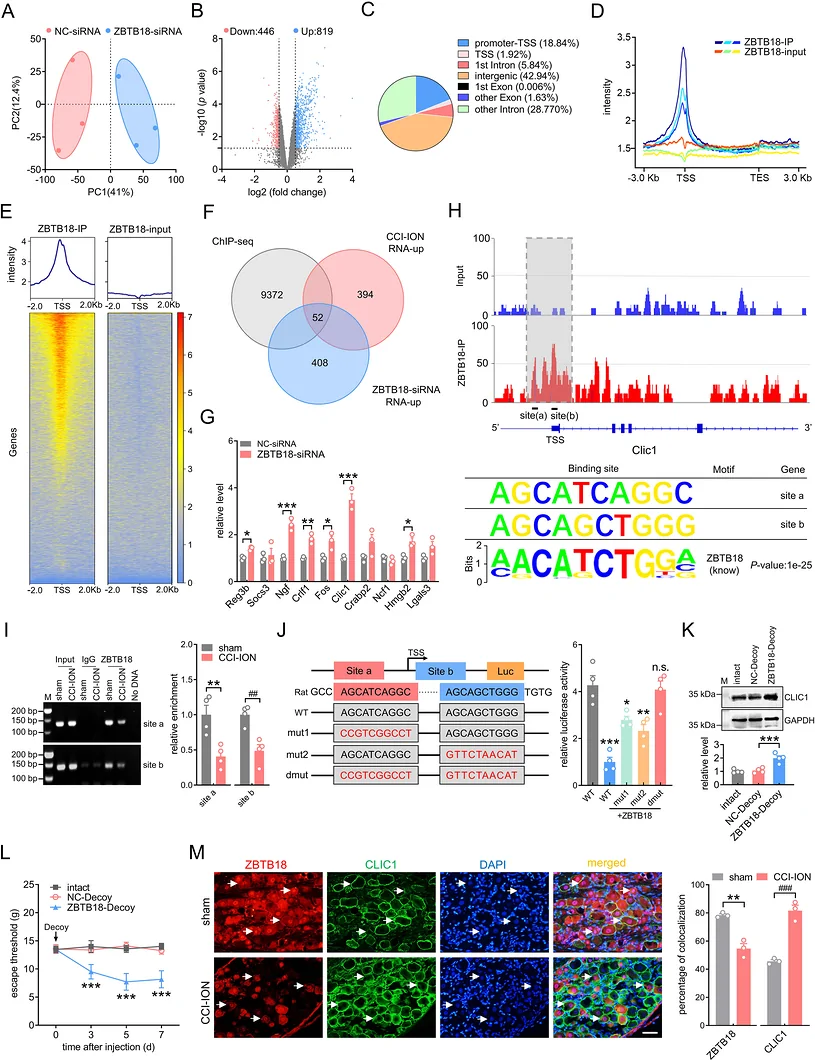
CLIC1 is a downstream target of ZBTB18. (A) Principal component analysis (PCA) plot showing distinct transcriptomic profiles between TG tissues treated with NC‐siRNA and ZBTB18‐siRNA. *n* = 6 rats per group. (B) Volcano plot illustrating transcripts upregulated (*blue*) and downregulated (*red*) in ZBTB18‐siRNA‐treated TGs compared to NC‐siRNA controls. (C) Genomic distribution of ZBTB18 binding peaks identified by ChIP‐seq, annotated to promoter‐TSS, transcription termination site (TTS), exonic, intronic, and intergenic regions. (D) Average profile of ZBTB18 ChIP‐seq signals across gene bodies, plotted from 3 kb upstream of the TSS to 3 kb downstream of the transcription end site (TES). (E) Heatmap showing genome‐wide ZBTB18 ChIP‐seq signal around TSS in TGs. 2‐kb region flanking the TSS is plotted. Color gradient reflects relative enrichment levels. (F) Integrative analysis of ChIP‐seq targets with genes upregulated after CCI‐ION or ZBTB18 knockdown. (G) qPCR validation of the top ten candidate target genes upregulated in ipsilateral TGs 3 days after ZBTB18‐siRNA injection. *p < 0.05, **p < 0.01, ***p < 0.001 compared with corresponding NC‐siRNA group, multiple *t*‐test, *n* = 3 rats/group. (H) Browser tracks of ZBTB18 ChIP‐seq signals at the *Clic1* promoter. Two binding motifs (site a and site b) identified by HOMER are indicated. (I) ChIP‐qPCR quantification of ZBTB18 occupancy at *Clic1* promoter sites a and b in TGs on day 14 after CCI‐ION or sham surgery. **p < 0.01 for site a compared with sham group; ^##^ *p* < 0.01 for site b compared with sham group, multiple *t*‐test. *n* = 16 rats/group. Representative data from four experiments. (J) Schematic of luciferase reporter constructs (*left*) and their relative activities (*right*) for wild‐type (WT) and ZBTB18‐binding site mutants (mut1, mut2, dmut) of the *Clic1* promoter. *p < 0.05, **p < 0.01, ***p < 0.001 compared with WT group, one‐way ANOVA, *n* = 4. (K) Immunoblot analysis of CLIC1 protein expression in TGs 3 days after intra‐TG delivery of ZBTB18‐Decoy or NC‐Decoy. Shown blots are representative of four independent experiments. ***p < 0.001 compared with NC‐Decoy group, one‐way ANOVA, *n* = 4 rats/group. (L) Mechanical hypersensitivity induced by intra‐TG injection of ZBTB18‐Decoy in intact rats. ***p < 0.001 compared with corresponding NC‐Decoy group, two‐way ANOVA, *n* = 8 rats/group. (M) Immunofluorescence staining of ZBTB18 (*red*) and CLIC1 (*green*) in ipsilateral TGs on day 14 after sham or CCI‐ION operation. Nuclei were counterstained with DAPI (*blue*). Arrows indicate co‐localization (*left panel*). Quantification shows the proportion of ZBTB18^+^/CLIC1^+^ neurons among DAPI^+^ cells (*right panel*). **p < 0.01 for ZBTB18 compared with sham group; ^###^ *p* < 0.001 for CLIC1 compared with sham group, multiple *t*‐test, *n* = 3 rats/group. Scale bar, 50 µm.

To elucidate the molecular mechanisms by which ZBTB18 regulates neuropathic pain, we sought to identify its downstream transcriptional targets by conducting RNA‐Seq on TG cells from intact rats treated with either NC‐siRNA or ZBTB18‐siRNA (GEO accession number GSE310186). Principal component analysis revealed a clear separation between the two groups, demonstrating that ZBTB18 knockdown significantly reshapes the global transcriptional landscape (Figure 3A). Among the 1265 differentially expressed genes (DEGs; *p* < 0.05, |log_2_foldchange| >0.5), the vast majority were significantly upregulated following ZBTB18 depletion (Figure 3B), supporting a predominantly repressive role for ZBTB18 in this context. We next identified direct transcriptional targets of ZBTB18 by ChIP‐seq analysis and obtained 67 318 high‐confidence binding peaks (GEO accession number GSE310187). Genomic annotation revealed that these peaks were predominantly located in intronic (34.61%), intergenic (42.94%), and promoter regions (20.76%), with a smaller fraction in exonic regions (1.64%) (Figure 3C). Notably, we observed a significant enrichment of ZBTB18 binding signals at promoter‐transcription start site (TSS) regions compared to the input control (Figure 3D, E). Gene Ontology (GO) molecular function enrichment analysis further showed that ZBTB18‐bound genes were prominently associated with ion channel activity and inorganic anion transmembrane transporter activity, while genes upregulated upon ZBTB18 knockdown were enriched for ion channel activity, anion binding and intracellular chloride channel activity (Figure S11). Overlaying these ChIP‐seq targets with RNA‐seq data from ZBTB18‐knockdown and CCI‐ION groups yielded 52 high‐confidence direct targets of ZBTB18 (Figure 3F). qPCR validation of the top 10 candidates showed that six genes (*Reg3b, Ngf, Crlf1, Fos, Clic1*, and *Hmgb2*) were significantly elevated in the TG after ZBTB18‐siRNA injection, with *Clic1* displaying the strongest upregulation (∼348.4%; Figure 3G). We further validated the expression of these candidate genes in ipsilateral TG tissues 14 days after CCI‐ION surgery. qPCR results confirmed that CLIC1 exhibited the most pronounced upregulation among all candidates, suggesting that it may serve as a key downstream effector (Figure S12). Accordingly, ChIP‐seq confirmed clear ZBTB18 binding enrichment at the *Clic1* promoter (Figure 3H). Furthermore, a *de novo* motif analysis with HOMER revealed a 10‐nucleotide consensus sequence for ZBTB18 (Figure 3H). This motif was mapped to two distinct sites within the Clic1 promoter (site a: −473 to −482; site b: +38 to +47), suggesting putative direct binding sites for ZBTB18 (Figure 3H). We then assessed the binding dynamics of ZBTB18 at the Clic1 locus by ChIP–qPCR. The results showed that ZBTB18 occupancy at both sites was significantly reduced in injured TGs at 14 days after CCI‐ION, compared to the sham group (Figure 3I). To define the functional role of these sites in regulating Clic1 transcription, we generated a series of mutant promoters with disruptions in single (mut1, mut2) or both (dmut) binding sites and analyzed their activity using luciferase reporter assays. Overexpression of ZBTB18 suppressed the luciferase activity of the wild‐type (WT), mut1, and mut2 promoters; the mut1 and mut2 constructs exhibited similar partial reductions. In contrast, ZBTB18 failed to repress the activity of the dmut promoter (Figure 3J), indicating that the two binding sites mediate synergistic inhibitory effects on Clic1 transcription. To functionally test the regulatory impact of ZBTB18 binding on CLIC1 expression and nociception, we disrupted the ZBTB18‐promoter interaction in intact TGs using a unilateral infusion of a ZBTB18‐targeting decoy DNA (Figure S13A). To verify that this approach broadly blocks ZBTB18 transcriptional activity rather than producing gene‐specific off‐target effects, we performed unbiased RNA‐seq on decoy‐treated TG tissues. Transcriptomic profiling revealed that the majority of differentially expressed genes were significantly upregulated after ZBTB18‐decoy administration, with far fewer genes showing downregulation (Figure S13B). This expression signature is fully consistent with the established role of ZBTB18 as a transcriptional repressor, confirming that the decoy effectively relieves ZBTB18‐dependent gene silencing at the genome‐wide level. In line with our mechanistic model, Clic1 was among the significantly upregulated genes, a result further validated by qPCR (Figure S13C). At the protein level, administration of the ZBTB18‐decoy, but not the NC‐decoy, significantly upregulated CLIC1 abundance (Figure 3K), and concurrently provoked mechanical allodynia (Figure 3L). These findings collectively identify the ZBTB18 binding sites as functional silencers of Clic1 transcription in TG neurons. Corroborating this, immunostaining analysis revealed strong colocalization of ZBTB18 and CLIC1 in TG neurons under both sham and CCI‐ION conditions (Figure 3M). Notably, in CCI‐ION tissues, the majority of neurons exhibiting high CLIC1 expression showed markedly reduced ZBTB18 fluorescence intensity (Figure 3M), providing direct histological evidence for the inverse correlation between ZBTB18 loss and CLIC1 upregulation.

### 2.4 ZBTB18 Recruits CHD4‐NuRD to Regulate Clic1 Transcription

**FIGURE 4:**
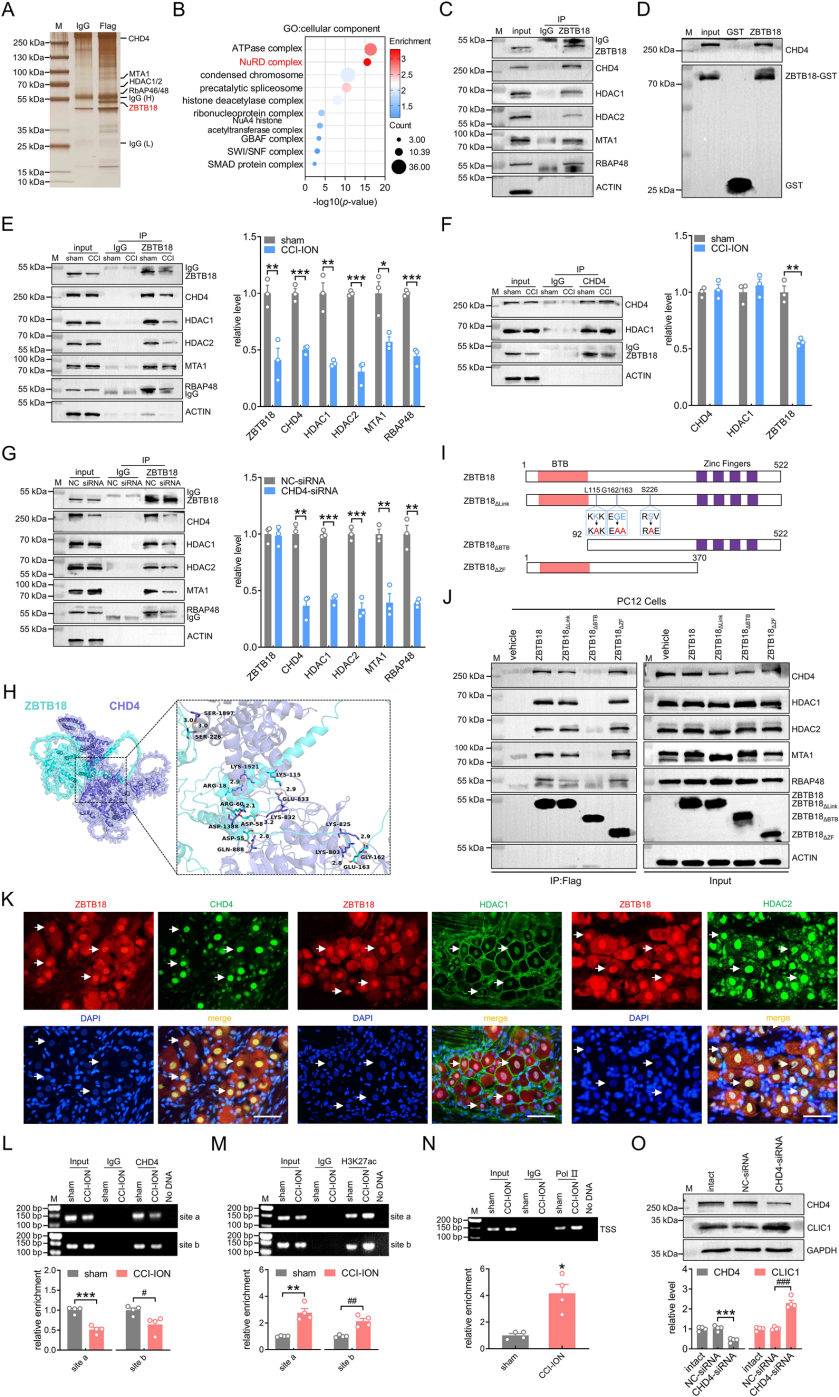
ZBTB18 recruits CHD4‐NuRD to regulate *Clic1* transcription. (A) Affinity purification of PC12 cells stably expressing Flag‐tagged ZBTB18. Eluates were separated by SDS–PAGE and silver‐stained, and specific bands were analyzed by mass spectrometry. (B) Gene Ontology (GO) analysis of enriched cellular component among the ZBTB18 interactome. (C) Co‐immunoprecipitation assays showing the interaction between ZBTB18 and subunits of NuRD complex in PC12 cell lysates using an anti‐ZBTB18 antibody. Representative blots from three experiments are shown. (D) GST pull‐down assay demonstrating a direct interaction between ZBTB18 and CHD4. Representative blots from two experiments are shown. (E) Validation of ZBTB18‐NuRD interaction in ipsilateral TGs 14 days after CCI‐ION or sham surgery. *p < 0.05, **p < 0.01, ***p < 0.001 compared with corresponding sham group, multiple *t*‐test, *n* = 6 rats/group. Representative blots from three experiments. (F) Co‐IP assays showing the interaction between CHD4 and HDAC1, and ZBTB18 in ipsilateral TGs 14 days after CCI‐ION or sham surgery. **p < 0.01 compared with corresponding sham group, multiple *t*‐test, *n* = 6 rats/group. Representative blots from three experiments. (G) Intra‐TG injection of CHD4‐siRNA inhibited the binding of ZBTB18 with NuRD subunits. **p < 0.01, ***p < 0.001 compared with corresponding NC‐siRNA group, multiple *t*‐test, *n* = 6 rats/group. Shown blots are representative of three independent experiments. (H) Predicted structural model of the ZBTB18‐CHD4 complex generated by GRAMM docking. (I) Schematic representation of full‐length ZBTB18 and its deletion mutants, highlighting the BTB domain (*red*) and zinc finger motifs (*purple*). (J) Immunoblot analysis of anti‐Flag immunoprecipitates (*left*) and whole‐cell lysates (*right*) from PC12 cells transfected with control vector, Flag‐ZBTB18, or its deletion mutants (ZBTBΔlink, ZBTBΔBTB, ZBTBΔZF). Blots were probed with indicated antibodies (representative of three experiments). (K) Immunofluorescence co‐localization of ZBTB18 (*red*) with CHD4, HDAC1, or HDAC2 (*green*) in TG neurons of intact rats. Arrows indicate co‐localization. Scale bar, 50 µm. (L) CHD4 occupancy decreases at the *Clic1* promoter in TGs on day 14 following CCI‐ION. ***p < 0.001 for site a compared with sham group; ^#^ *p* < 0.05 for site b compared with sham group, multiple *t*‐test. *n* = 16 rats/group. Representative data from four experiments. (M) Concurrent increase in H3K27ac enrichment at the *Clic1* promoter after nerve injury. **^/##^ *p* < 0.01 for both sites compared with sham, multiple *t*‐test, *n* = 16 rats/group. Representative blots from four experiments. (N) Enhanced RNA polymerase II recruitment to *Clic1* TSS after CCI‐ION. *p < 0.05 compared with sham group, unpaired *t*‐test, *n* = 16 rats/group. Representative blots from four experiments. (O) Intra‐TG injection of CHD4‐siRNA reduced CHD4 protein levels and increased CLIC1 expression. ***p < 0.001 for CHD4 compared with NC‐siRNA group; ^###^ *p* < 0.001 for CLIC1 compared with NC‐siRNA group, one‐way ANOVA, *n* = 4 rats/group. Representative blots from four experiments.

Transcriptional repression by sequence‐specific DNA‐binding proteins typically involves the recruitment of co‐repressor complexes [37, 38, 39]. To investigate the mechanistic basis for ZBTB18 dysfunction leading to CLIC1 upregulation in injured TGs, we expressed Flag‐tagged ZBTB18 in PC12 cells and immunoprecipitated it for LC–MS analysis to identify associated proteins (Figure 4A). Further Gene Ontology (GO) analysis indicated significant enrichment of the ZBTB18 interactome in components of the nucleosome remodeling and deacetylase (NuRD) complex (Figure 4B). Notably, among the four major mammalian chromatin remodeler families—INO80, ISWI, NuRD, and SWI/SNF—which modulate transcription through nucleosome mobilization, sliding, or eviction [40, 41], ZBTB18 specifically associated with multiple core NuRD subunits, including the chromatin remodeler CHD4, histone deacetylases HDAC1/2, MTA1, and RBAP46/48 (Figure S14). This interaction between ZBTB18 and the NuRD complex was further validated endogenously by co‐immunoprecipitation (Figure 4C). To delineate the direct intermolecular interactions between ZBTB18 and the NuRD complex, we performed in vitro pull‐down assays. Using bacterially expressed and purified GST‐tagged ZBTB18 and FLAG‐tagged NuRD subunits (CHD4, HDAC1/2, MTA1, and RBAP48), GST pull‐down assays identified a specific direct interaction between ZBTB18 and CHD4 (Figure 4D), but not with the other subunits (Figure S15). Consistent with this, co‐immunoprecipitation in native TG tissues revealed a physiological ZBTB18–NuRD corepressor complex, which was markedly weakened following CCI‐ION injury (Figure 4E). Importantly, although the protein levels of NuRD subunits (CHD4, HDAC1/2, MTA1, and RBAP48) were unaltered by nerve injury (Figure S16), co‐IP with an anti‐CHD4 antibody revealed a specific disruption of the ZBTB18–CHD4 interaction in injured TGs, while the CHD4–HDAC1 association remained intact (Figure 4F). These results establish that a ZBTB18–NuRD corepressor complex exists in rat TG neurons and that its integrity depends on direct physical interactions between ZBTB18 and CHD4. To test this model, we asked whether CHD4 depletion would disrupt the complex in vivo. We applied the same siRNA screening strategy as described above and selected siRNA‐4 (CHD4‐siRNA) from four independent candidates for subsequent functional experiments (Figure S17). Unilateral intra‐TG injection of CHD4‐siRNA, but not NC‐siRNA, into intact TGs markedly reduced the association of ZBTB18 with other NuRD components (Figure 4G).

Furthermore, to identify the ZBTB18 domain essential for CHD4 binding, we combined computational and experimental approaches. Docking and molecular dynamics simulations performed with the GRAMM‐X server indicated that ZBTB18 interacts with CHD4 via six hydrogen bonds and two electrostatic interactions, forming a stable dimer (Figure 4H & Figure S18). As guided by the GRAMM‐X docking model, CHD4‐interacting residues were found to cluster predominantly within the BTB domain (Arg18/60 and Asp55/58), with an additional dispersed set of residues located in the linker region (Lys115, Gly162, Glu163, Ser226). Based on this domain‐level distribution, we generated ZBTB18ΔBTB, ZBTB18Δlink, and an additional ZBTB18ΔZF mutant to selectively interrogate the contribution of each structural region to CHD4 interaction (Figure 4I). Co‐immunoprecipitation assays revealed that neither deletion of the C‐terminal zinc fingers (ZBTB18ΔZF) nor mutation of linker residues (ZBTB18Δlink) disrupted NuRD binding (Figure 4J). In contrast, deletion of the BTB domain (ZBTB18ΔBTB) completely abolished co‐immunoprecipitation with core NuRD components—CHD4, HDAC1/2, MTA1, and RBAP48 (Figure 4J)—demonstrating that ZBTB18 directly interacts with CHD4 through its N‐terminal BTB domain to assemble a ZBTB18–NuRD corepressor complex at the *Clic1* promoter. Supporting this conclusion, immunostaining of rat TG tissues showed strong colocalization of ZBTB18 with key NuRD components, including CHD4, HDAC1, and HDAC2, in TG neurons (Figure 4K). We next explored how the ZBTB18–CHD4 complex represses *Clic1* transcription. Compared to sham‐operated controls, injured TGs at 14 days after CCI‐ION exhibited significantly reduced CHD4 occupancy at ZBTB18‐binding sites in the *Clic1* promoter (Figure 4L), along with a marked increase in H3K27ac enrichment (Figure 4M) and elevated RNA polymerase II (Pol II) occupancy at the Clic1 promoter‐transcription start site (TSS) (Figure 4N). Consistent with these findings, administration of CHD4‐siRNA, but not NC‐siRNA, markedly elevated CLIC1 protein levels (Figure 4O). We further determined whether disruption of the ZBTB18‐NuRD complex specifically modulates injury‐induced *Clic1* transcriptional activation. ZBTB18 overexpression in injured TGs restored CHD4 binding (Figure S19A) and reversed the CCI‐ION‐induced increase in H3K27ac levels (Figure S19B) and Pol II occupancy at the Clic1 promoter (Figure S19C). Conversely, in intact rat TGs, ZBTB18 knockdown—mimicking the injury‐induced reduction in ZBTB18—recapitulated the transcriptional derepression phenotype, reducing CHD4 binding (Figure S20A), increasing H3K27ac enrichment (Figure S20B), and enhancing Pol II occupancy (Figure S20C). To establish the in vivo functional necessity of NuRD complex recruitment for ZBTB18‐mediated pain regulation, we performed a four‐group in vivo behavioral assay. All rats received intra‐TG injection of CHD4‐siRNA or NC‐siRNA on days 0, 3 and 6, and were co‐administered ZBTB18‐up on days 3 and 6 (Figure S21). In sham‐operated rats, selective CHD4 silencing was sufficient to induce significant mechanical allodynia, confirming that intact NuRD function is required to maintain basal nociceptive thresholds and target gene repression (Figure S21). In CCI‐ION rats, pre‐knockdown of CHD4 completely abolished the analgesic effect of ZBTB18 overexpression (Figure S21), demonstrating that the pain‐regulatory function of ZBTB18 is strictly dependent on its recruitment of the CHD4‐containing NuRD complex. In contrast, intra‐TG administration of the selective class 1 HDAC inhibitor entinostat failed to induce significant nociceptive hypersensitivity in intact rats (Figure S22), indicating that HDAC‐mediated deacetylase activity alone cannot fully account for the transcriptional repressive function of the ZBTB18‐NuRD complex.

### 2.5 CLIC1 Channels Participate in Regulating Neuropathic Pain Behaviors

**FIGURE 5:**
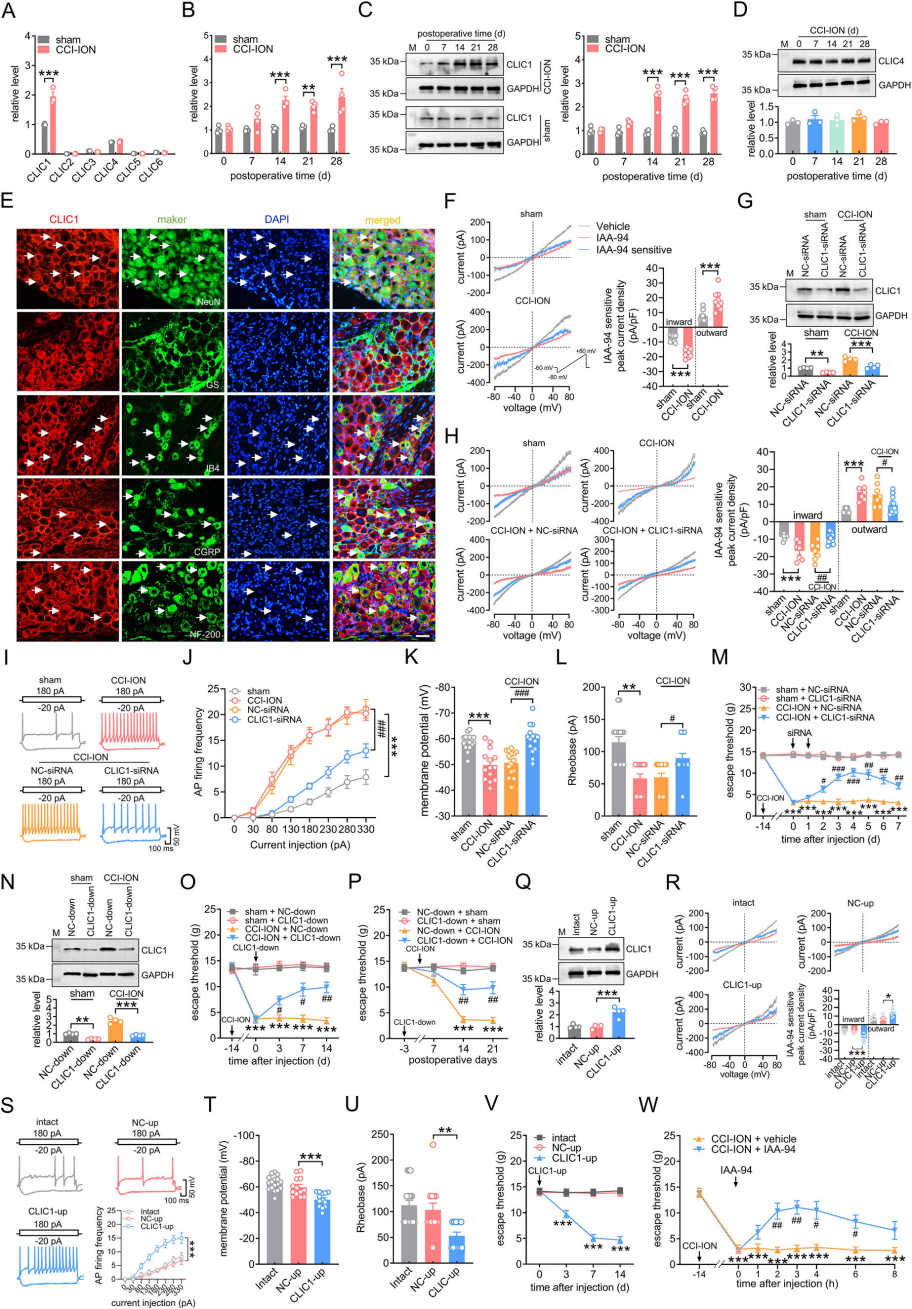
CLIC1 channels participate in regulating neuropathic pain behaviors. (A) qPCR analysis of Clics mRNA expression in ipsilateral TGs on day 14 following CCI‐ION or sham operation. Data were normalized to Clic1 mRNA levels in TGs from sham‐operated rats at day 14. ***p < 0.001 compared with corresponding sham group, multiple *t*‐test, *n* = 3 rats/group. (B) Time course of Clic1 mRNA expression. Data were analyzed by two‐way ANOVA. **p < 0.01, ***p < 0.001 compared with corresponding sham group, *n* = 4 rats/time point/group. (C) Immunoblot analysis of CLIC1 protein expression. ***p < 0.001 compared with corresponding sham group, two‐way ANOVA, *n* = 4 rats/time point/group. Representative blots from four experiments. (D) Immunoblot analysis of CLIC4 protein expression. *p* >0.05 compared with day 0 group, one‐way ANOVA, *n* = 3 rats/time point. Shown blots are representative of three independent experiments. (E) Immunofluorescence colocalization of CLIC1 (*red*) with markers (NeuN, GS, IB4, CGRP, or NF200; *green*) in TGs of intact rats. Arrows indicate colocalization. Scale bar, 50 µm. (F) Representative current traces and summary data of CLIC currents recorded from retrograde‐labeled small‐sized TG neurons. ***p < 0.001 compared with sham group, unpaired *t*‐test, *n* = 9–11 neurons from 4 rats. (G) CLIC1‐siRNA injection suppressed the CCI‐ION–induced upregulation of CLIC1 protein in ipsilateral TGs at day 14. Shown blots are representative of four independent experiments. **p < 0.01, ***p < 0.001 compared with corresponding NC‐siRNA group, one‐way ANOVA, *n* = 4 rats/group. (H) Representative traces and summary data showing that CLIC1‐siRNA abolished the increase in CLIC currents in small TG neurons following CCI‐ION. ***p < 0.001 compared with corresponding sham group; ^#^ *p* < 0.05, ^##^ *p* < 0.01 compared with corresponding CCI‐ION + NC‐siRNA group, one‐way ANOVA, *n* = 7–12 neurons from 4 rats. (I,J) Representative traces (*I*) and summary data (*J*) demonstrating that CLIC1‐siRNA attenuated the increase in action potential firing frequency following CCI‐ION. ***p < 0.001 compared with sham group; ^###^ *p* < 0.001 compared with CCI‐ION + NC‐siRNA group, two‐way ANOVA, *n* = 14–16 neurons from 5 rats. (K,L) Resting membrane potential (*K*) and rheobase (*L*) of TG neurons. **p < 0.01, ***p < 0.001 compared with sham group; ^#^ *p* < 0.05, ^###^ *p* < 0.001 compared with CCI‐ION + NC‐siRNA group, one‐way ANOVA, *n* = 14–16 neurons from 5 rats. (M) Intra‐TG injection of CLIC1‐siRNA ameliorated mechanical allodynia in CCI‐ION rats. ***p < 0.001 compared with corresponding sham + NC‐siRNA group; ^#^ *p* < 0.05, ^##^ *p* < 0.01, ^###^ *p* < 0.001 compared with corresponding CCI‐ION + NC‐siRNA group, two‐way ANOVA, *n* = 8 rats/group. (N) Lentiviral‐mediated CLIC1‐shRNA (CLIC1‐down) prevented CCI‐ION–induced CLIC1 protein upregulation. **p < 0.01, ***p < 0.001 compared with corresponding NC‐down group, one‐way ANOVA, *n* = 4 rats/group. Shown blots are representative of four independent experiments. (O) CLIC1‐down alleviated mechanical allodynia in CCI‐ION rats. ***p < 0.001 compared with corresponding sham + NC‐down group; ^#^ *p* < 0.05, ^##^ *p* < 0.01 compared with corresponding CCI‐ION + NC‐down group, two‐way ANOVA, *n* = 8–9 rats/group. (P) CLIC1‐down pretreatment reversed CCI‐ION–induced reduction in mechanical threshold. ***p < 0.001 compared with corresponding NC‐down + sham group; ^##^ *p* < 0.01 compared with corresponding NC‐down + CCI‐ION group, two‐way ANOVA, *n* = 8 rats/group. (Q) Intra‐TG delivery of lenti‐hSyn‐CLIC1‐up (CLIC1‐up) increased CLIC1 protein levels in intact rats. ***p < 0.001 compared with NC‐up group, one‐way ANOVA, *n* = 4 rats/group. (R) Representative traces and summary data showing that CLIC1‐up enhanced CLIC currents. *p < 0.05, ***p < 0.001 compared with NC‐up group, one‐way ANOVA, *n* = 7–11 neurons from 4 rats. (S) Example traces and summary data indicating that CLIC1‐up increased action potential firing frequency in intact rats. ***p < 0.001 compared with NC‐up group, two‐way ANOVA, *n* = 13–17 neurons from 5 rats. (T,U) Resting membrane potential (*T*) and rheobase (*U*) of TG neurons. **p < 0.01, ***p < 0.001 compared with NC‐up group, one‐way ANOVA, *n* = 13–17 neurons from 5 rats. (V) CLIC1‐up induced mechanical hypersensitivity in intact rats. ***p < 0.001 compared with NC‐up group, two‐way ANOVA, *n* = 8–10 rats/group. (W) IAA‐94 employment attenuated CCI‐ION–induced mechanical allodynia. *p < 0.05, **p < 0.01 compared with corresponding CCI‐ION + vehicle group, two‐way ANOVA, *n* = 8 rats/group.

We next assessed the functional role of TG CLIC1 in neuropathic pain behaviors. The CLIC protein family includes six conserved members (CLIC1–CLIC6) [42]. Among these, qPCR analysis detected substantial mRNA expression of only *Clic1* and *Clic4* in rat TGs (Figure 5A). Notably, *Clic1*—but not *Clic4*—was selectively upregulated in ipsilateral TGs at 14 days after CCI‐ION, with elevated expression sustained through day 28 (Figure 5A,B). Consistent with transcriptional data, CLIC1 protein levels were markedly increased in injured TGs on days 14, 21, and 28 post‐CCI‐ION (Figure 5C), whereas CLIC4 protein remained unchanged (Figure 5D). No changes in *Clic1* mRNA or CLIC1 protein were observed in sham‐operated (Figure 5B, C) or contralateral TG tissues (Figure S23). We further validated this expression pattern in female rats and observed a consistent time‐dependent upregulation of CLIC1 at both the mRNA and protein levels in the injured TG (Figure S24). We next characterized CLIC1 expression patterns in intact TG neurons. Immunostaining showed that CLIC1 was primarily expressed in neurons (NeuN^+^) and rarely in satellite glial cells (GS^+^) (Figure 5E). Statistical analysis revealed that ∼84.1% of the CLIC1^+^ TG neurons were labeled by NeuN, and ∼8.8% were labeled by GS (Figure S25). Moreover, CLIC1 was co‐expressed with CGRP and IB4, indicating localization in nociceptive neurons, with comparatively less overlap in NF200‐positive cells (Figure 5E). Approximately 43.22% of the CLIC1‐positive neurons were labeled by IB4, ∼34.58% by CGRP, and ·∼20.48% by NF200 (Figure S25).

We next assessed whether nerve injury‐induced CLIC1 upregulation alters chloride currents in DiI‐retrolabeled small TG neurons. Nerve injury significantly enhanced CLIC channel activity, increasing the inward peak current density from −7.74 ± 2.13 pA/pF (sham) to −17.7 ± 2.92 pA/pF (CCI‐ION), and the outward peak current density from 7.52 ± 2.92 pA/pF to 18.23 ± 6.62 pA/pF (Figure 5F). To determine whether this effect was specifically mediated by CLIC1, we applied the same siRNA screening strategy as described above and selected siRNA‐344 (CLIC1‐siRNA) from four independent candidates for subsequent functional experiments (Figure S26). We then knocked down CLIC1 expression in injured TGs using this chemically modified siRNA, and found that administration of CLIC1‐siRNA completely abolished the injury‐induced increase in both CLIC1 protein (Figure 5G) and CLIC currents (Figure 5H). Electrophysiological recordings of DiI‐labeled small TG neurons demonstrated a state of hyperexcitability in the CCI‐ION rats, characterized by increased action potential firing frequency (Figure 5I,J), depolarized resting membrane potential (Figure 5K), and reduced rheobase (Figure 5L). These changes were fully reversed by CLIC1‐siRNA, but not by control siRNA (Figure 5I–L). Consistent with a role in pain behavior, CLIC1 knockdown alleviated CCI‐ION–induced mechanical allodynia for up to 7 days post‐injection (Figure 5M), and this analgesic effect on mechanical allodynia was consistently replicated in female CCI‐ION rats (Figure S27). In addition, CLIC1 silencing also significantly reversed CCI‐ION‐induced thermal hyperalgesia in both male and female rats (Figure S28), further corroborating the pronociceptive function of CLIC1 in trigeminal neuropathic pain. We extended these findings using lentiviral‐mediated CLIC1 shRNA (CLIC1‐down). When administered 14 days after CCI‐ION, CLIC1‐down suppressed CLIC1 protein expression (Figure 5N) and attenuated mechanical allodynia from day 3 to day 14 (Figure 5O). Pretreatment with CLIC1‐down before nerve injury also reduced the development of neuropathic pain, with significant antiallodynic effects observed from days 14 to 21 post‐surgery (Figure 5P). Conversely, CLIC1 overexpression (CLIC1‐up) in intact TGs increased both CLIC1 protein (Figure 5Q) and CLIC currents (Figure 5R), and induced neuronal hyperexcitability resembling the injury phenotype—evidenced by elevated firing frequency, depolarized resting potential, and lower rheobase (Figure 5S–U). CLIC1‐up also elicited mechanical allodynia from day 3 to day 14 (Figure 5V), whereas control virus had no effect. Given that membrane‐localized CLIC1 functions as a chloride channel, we evaluated the chloride channel blocker IAA‐94 as a potential therapeutic agent. Systemic administration (i.p.) of IAA‐94 on day 14 after CCI‐ION significantly alleviated mechanical allodynia for 2–6 h post‐injection (Figure 5W).

### 2.6 ZBTB18 Regulates Nociceptive Behaviors via CLIC1 Channels

**FIGURE 6:**
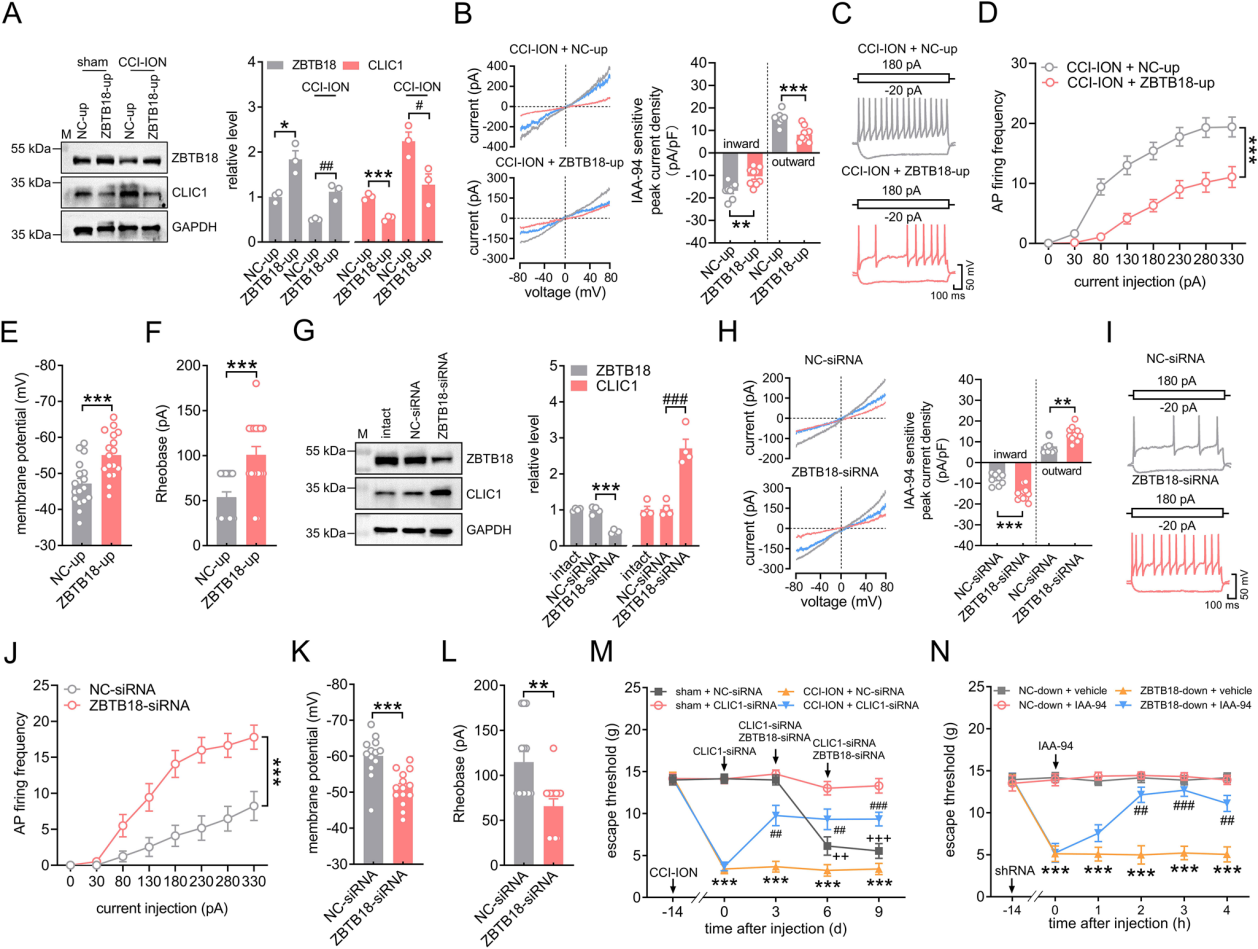
ZBTB18 regulates nociceptive behaviors via CLIC1 channels. (A) ZBTB18‐up abolished the downregulation of ZBTB18 and upregulation of CLIC1 protein following CCI‐ION. *p < 0.05, ***p < 0.001 compared with corresponding sham + NC‐up group; ^#^ *p* < 0.05, ^##^ *p* < 0.01 compared with corresponding CCI‐ION + NC‐up group, one‐way ANOVA, *n* = 3 rats/group. Representative blots from three experiments. (B) Representative traces and summary data of CLIC currents in TG neurons treated with ZBTB18‐up on day 14 post‐CCI‐ION. **p < 0.01, ***p < 0.001 compared with corresponding CCI‐ION + NC‐up group, one‐way ANOVA, *n* = 8–9 neurons from 3 rats. (C,D) Example traces (*C*) and summary data (*D*) showing that ZBTB18‐up reduced the action potential firing frequency in TG neurons on day 14 after CCI‐ION. ***p < 0.001 compared with CCI‐ION + NC‐up group, two‐way ANOVA, *n* = 17 neurons from 5 rats. (E,F) Resting membrane potential (*E*) and rheobase (*F*) of TG neurons. ***p < 0.001 compared with corresponding CCI‐ION + NC‐up group, unpaired *t*‐test, *n* = 17 neurons from 5 rats. (G) Knockdown of ZBTB18 reduced ZBTB18 and increased CLIC1 protein levels in TGs of intact rats. ***p < 0.001 for ZBTB18 compared with NC‐siRNA group; ^###^ *p* < 0.001 for CLIC1 compared with NC‐siRNA group, one‐way ANOVA, *n* = 4 rats/group. Representative blots from four experiments. (H) Example traces and summary data of CLIC currents in TG neurons treated with ZBTB18‐siRNA. **p < 0.01, ***p < 0.001 compared with corresponding NC‐siRNA group, one‐way ANOVA, *n* = 9–11 neurons from 4 rats. (I,J) Exemplary traces (*I*) and summary data (*J*) showing that ZBTB18‐siRNA increased the action potential firing frequency in TG neurons from intact rats. ***p < 0.001 compared with NC‐siRNA group, two‐way ANOVA, *n* = 13–14 neurons from 5 rats. (K,L) Resting membrane potential (*K*) and rheobase (*L*) of TG neurons. **p < 0.01, ***p < 0.001 compared with corresponding NC‐siRNA group, unpaired *t*‐test, *n* = 13–14 neurons from 5 rats. (M) CLIC1‐siRNA pretreatment prevented the exacerbation of mechanical allodynia induced by ZBTB18‐siRNA. ***p < 0.001 compared with corresponding sham + NC‐siRNA group; ^##^ *p* < 0.01, ^###^ *p* < 0.001 compared with corresponding CCI‐ION + NC‐siRNA group; ^++^ *p* < 0.01, ^+++^ *p* < 0.001 compared with sham + NC‐siRNA on day 0, two‐way ANOVA, *n* = 8 rats/group. (N) IAA‐94 completely abolished ZBTB18‐down–induced mechanical allodynia in intact rats. ***p < 0.001 compared with corresponding NC‐down + vehicle group; ^##^ *p* < 0.01, ^###^ *p* < 0.001 compared with corresponding ZBTB18‐down + vehicle group, two‐way ANOVA, *n* = 8 rats/group.

We next assessed whether modulating ZBTB18 expression in injured TGs would affect CLIC1 protein levels and functional activity. Intra‐TG delivery of ZBTB18‐up—but not the negative control (NC‐up)—significantly reversed the CCI‐ION–induced upregulation of CLIC1 expression at day 14 post‐injury (Figure 6A). Electrophysiological recordings from DiI‐labeled small‐diameter neurons showed that ZBTB18 overexpression prevented the injury‐induced enhancement of CLIC1 channel currents (Figure 6B) and suppressed neuronal hyperexcitability (Figure 6C,D), as evidenced by a lower firing frequency of action potentials (Figure 6D), a hyperpolarized resting membrane potential (Figure 6E), and an elevated rheobase (Figure 6F). To further determine whether ZBTB18 downregulation is sufficient to drive CLIC1 expression and neuronal excitability, we administered ZBTB18‐siRNA into intact TGs. Immunoblot analysis confirmed a marked increase in CLIC1 protein levels following ZBTB18 knockdown (Figure 6G). Correspondingly, ZBTB18 silencing enhanced CLIC1‐mediated currents (Figure 6H) and induced hyperexcitable phenotypes in small TG neurons (Figure 6I), including increased action potential firing (Figure 6J), depolarized resting membrane potential (Figure 6K), and reduced rheobase (Figure 6L). To determine whether CLIC1 is a downstream effector mediating ZBTB18‐dependent nociceptive behaviors, CLIC1‐siRNA was administered simultaneously with ZBTB18‐siRNA into the ipsilateral TGs following CCI‐ION (Figure 6M). Chemically modified CLIC1‐siRNA was delivered on days 0, 3, and 6, while ZBTB18‐siRNA was administered on days 3 and 6. Compared to NC‐siRNA controls, CLIC1 knockdown initiated on day 0 significantly attenuated CCI‐ION‐induced mechanical allodynia by day 3. Subsequent ZBTB18 silencing (from day 3 onward) failed to further alter nociceptive thresholds in CLIC1‐deficient rats, as evidenced by stable escape thresholds from days 3 to 9. In contrast, ZBTB18 knockdown in sham‐operated rats pretreated with NC‐siRNA markedly reduced mechanical pain thresholds. These results indicate that CLIC1 is required for ZBTB18‐mediated neuropathic pain. Supporting this conclusion, pharmacological blockade of CLIC1 channels with the inhibitor IAA‐94 completely reversed ZBTB18 knockdown‐induced mechanical allodynia within 4 h o after intraperitoneal injection in intact rats (Figure 6N). Together, these results demonstrate that ZBTB18 regulates trigeminal neuropathic pain through CLIC1‐dependent pathways, modulating both CLIC1 expression and functional channel activity.

## 3 Discussion

**FIGURE 7:**
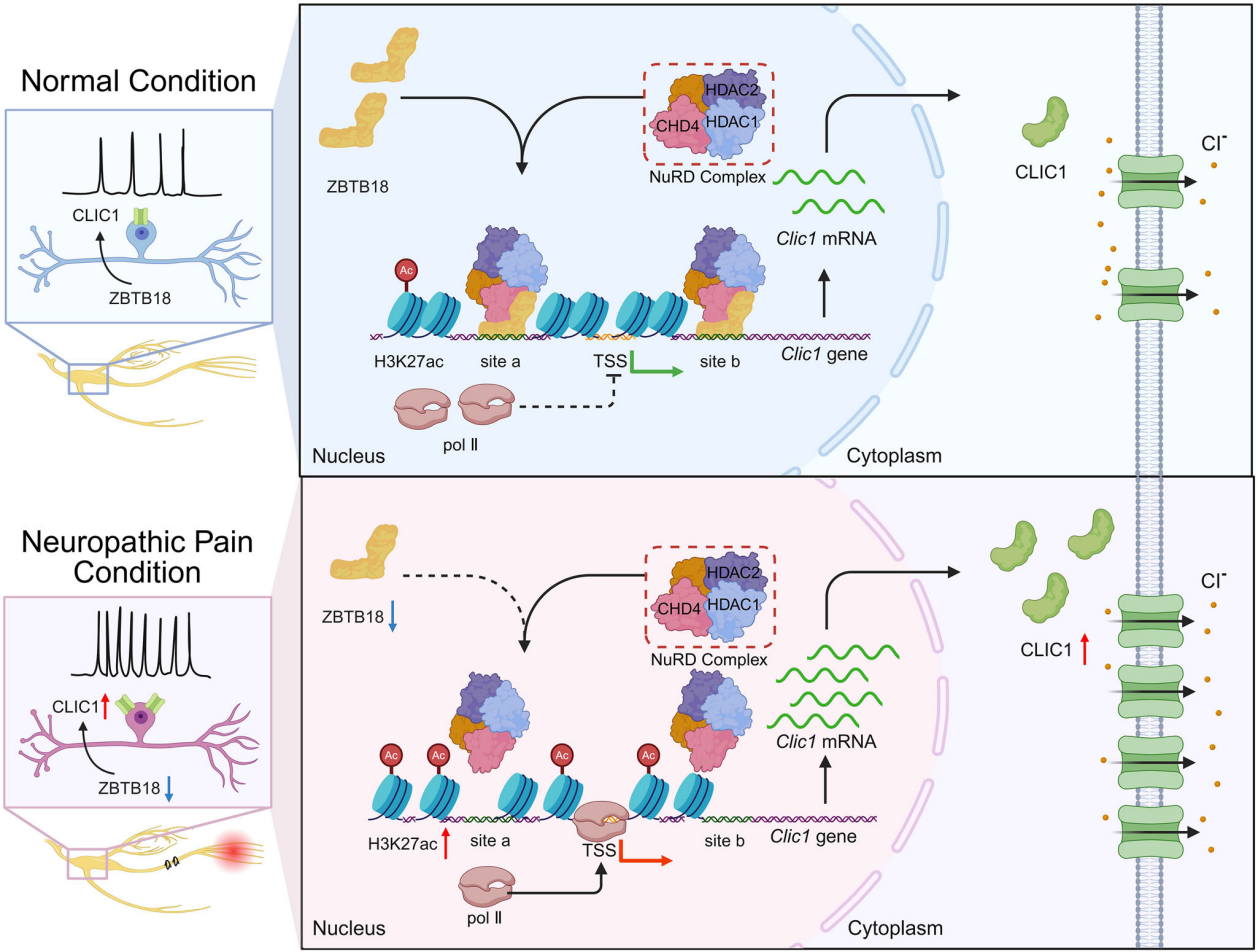
Schematic illustration of ZBTB18‐mediated regulation in neuropathic pain. Under physiological conditions (*upper panel*), TG neurons exhibit high expression of the transcription factor ZBTB18. Through its N‐terminal BTB/POZ domain, ZBTB18 interacts with CHD4, a core component of the NuRD complex, facilitating the recruitment of this transcriptional repressor complex to the Clic1 gene promoter. This recruitment establishes a transcriptionally repressive chromatin state characterized by low H3K27ac levels, thereby suppressing RNA polymerase II (Pol II) binding and Clic1 gene transcription. Consequently, CLIC1 expression remains at basal levels, maintaining normal chloride currents and preserving physiological neuronal excitability in TG neurons. Following CCI‐ION (*lower panel*), ZBTB18 expression is significantly downregulated in TG neurons. This reduction disrupts ZBTB18‐CHD4 interaction, impairing NuRD complex recruitment to the Clic1 promoter. The loss of chromatin repression leads to increased H3K27ac accumulation and enhanced RNA polymerase II occupancy, resulting in robust Clic1 transcriptional activation. The subsequent upregulation of CLIC1 protein and chloride channel currents promotes neuronal hyperexcitability, ultimately driving the development of trigeminal neuropathic pain. Created with BioRender.

In this study, we provided the first evidence to our knowledge that ZBTB18 constrains TG neuronal CLIC1 expression via an epigenetic mechanism dependent on recruitment of the CHD4‐based NuRD complex. Peripheral nerve injury triggered ZBTB18 downregulation in injured TG neurons, which attenuated its binding to the promoter region of Clic1 and its association with the CHD4‐NuRD complex. Consequently, this led to increased H3K27ac accumulation and enhanced RNA polymerase II occupancy in Clic1 promoter, thereby elevating CLIC1 expression. The subsequent upregulation of CLIC1 protein and chloride channel currents promotes neuronal hyperexcitability, ultimately driving the development of trigeminal neuropathic pain. This work elucidated a novel silencing mechanism wherein injury‐induced ZBTB18 downregulation drives neuropathic pain by epigenetically disinhibiting *Clic1* transcription in TG neurons (Figure 7). Targeting the ZBTB18–NuRD–CLIC1 epigenetic axis may thus offer novel therapeutic strategies for clinical neuropathic pain management.

Peripheral nerve injury induces profound transcriptional reprogramming in primary sensory neurons, a process critical for the initiation and maintenance of neuropathic pain [43]. While previous studies have identified several transcription factors—such as activating transcription factor 3, Y‐box binding protein 3, and early growth response 2—that are upregulated in the TG following nerve injury and contribute to pain hypersensitivity [44, 45, 46], the present study identifies ZBTB18 as a transcription factor that is persistently downregulated in TG neurons after nerve injury. This sustained decrease suggests that ZBTB18 may serve as a molecular indicator of nerve injury and the maladaptive transcriptional changes underlying trigeminal neuropathic pain. In contrast to its well‐established role in the central nervous system (CNS) as a neuron‐enriched transcriptional repressor essential for neuronal differentiation, cortical lamination, and synaptic maturation [22, 47, 48], the function of ZBTB18 in the peripheral nervous system (PNS) remains poorly characterized. In this study, we clarified the cellular localization of ZBTB18 in the TG, demonstrating its predominant expression in NeuN‐positive neurons and absence in GS‐positive glial cells. This neuron‐specific expression pattern aligns with findings in the CNS, where ZBTB18 is expressed in regions including the cortical plate, hippocampus, and cerebellum [22, 24, 49], where it functions to maintain transcriptional homeostasis [50]. Importantly, recent single‐cell RNA sequencing has further detected ZBTB18 expression in human dorsal root ganglion sensory neurons, suggesting a conserved, neuron‐restricted expression profile across species [51]. Nonetheless, ZBTB18 expression has been reported in glioblastoma multiforme (GBM), a tumor type for which neural precursor*/stem cells* have been proposed as one of the potential cells of origin [28, 52]. Additionally, our present findings establish that nerve injury‐induced loss of ZBTB18—a key transcriptional stabilizer in neurons [21, 53, 54]—disrupts transcriptional equilibrium in peripheral sensory ganglia, thereby identifying its downregulation as a pivotal event in the transcriptional disinhibition underlying trigeminal neuropathic pain.

Despite advances in mapping transcription factor binding, the mechanisms by which DNA‐bound factors regulate target gene expression remain less clear. This process often involves coactivators or corepressors that “bridge” transcription factors to the basal transcriptional machinery or chromatin modifiers. This knowledge gap is particularly relevant for the BTB‐ZF family—comprising at least 49 proteins, many of which are implicated in nervous system function [55, 56]. Previous studies have reported that ZBTB family members, such as ZBTB27, a well‐characterized BTB‐ZF transcription factor, function primarily as transcriptional repressor [57], and such repressive functions are mediated by the recruitment of co‐repressors NCoR in glioblastoma tissue [58], while in non‐Hodgkin lymphomas tissue recruitment of SMRT to its BTB domain [59]. Moreover, another family member ZBTB1 mediates interaction with the transcriptional repressor KAP‐1 and facilitates the localization of phosphorylated KAP‐1 to chromatin, thereby coordinating chromatin remodeling during DNA repair [60]. Therefore, although the high structural homology among BTB domains of different ZBTB transcription factors—such as ZBTB18, ZBTB27 and ZBTB1 [61, 62]—these domains may seem to exert variable repressive activities by recruiting distinct cofactors. In the present study, we identified ZBTB18‐interacting proteins by co‐immunoprecipitation combined with LC–MS and subsequently confirmed, through in vitro GST pull‐down assays, that in TG neurons, ZBTB18 recruits the NuRD complex through a direct interaction with CHD4 mediated by its BTB domain, and mediates a transcriptional repression of CLIC1 in TG neurons. Thus, BTB domains within the BTB‐ZF transcription factor family are not functionally equivalent, but instead confer distinct regulatory properties that, together with zinc finger‐mediated DNA recognition, factor‐specific expression patterns, and the recruitment of additional cofactors through other protein domains, collectively determine the functional diversity of these transcription factors [61]. Interestingly, ZBTB18 has been reported to act as a transcriptional activator in the neonatal rat pineal gland, where its knockdown reduced expression of melatonin‐synthesis and circadian‐regulated genes [63]; however, these results did not characterize its “bridge” transcription factor functions, and seem to be contradictory to those of most related studies [30, 50, 64]. More broadly, the function of ZBTB18 is not exclusively dependent on its BTB domain. An early study reported that ZBTB18 was identified in a yeast two‐hybrid screen as a direct interactor of DNMT3A, an interaction independent of the BTB domain [64]. In glioblastoma, ZBTB18 interacts with CTBP through its VLDLS motif to recruit HDAC1/2 and repress the expression of Sterol regulatory element–binding protein (SREBP) genes, whereas loss of this interaction promotes tumor malignancy [31]. Another study in the nervous system demonstrated that ZBTB18 forms a complex with FOXG1 during cortical development to cooperatively regulate downstream gene expression [53]. However, none of these direct interacting partners of ZBTB18 were detected in our mass spectrometry dataset. While the discrepancies warrant further studies, different tissue‐ and cell type–specific interaction spectrum of ZBTB18 may be considered.

Although ZBTB18 may have multiple molecular targets, our present findings establish a specific mechanistic link to CLIC1 channel function in trigeminal neuropathic pain. By integrating ChIP‐seq and RNA‐seq data from ZBTB18‐knockdown and CCI‐ION groups, we identified CLIC1 as one of the most significantly responsive genes to ZBTB18 regulation, and propose that the downregulation of ZBTB18 disrupts the occupancy of the ZBTB18–NuRD co‐repressor complex at the *Clic1* promoter region, leading to epigenetic de‐repression and enhanced CLIC1 transcription. CLIC1 belongs to the highly conserved CLIC family of six vertebrate isoforms and differs from classical ion channels by existing in both soluble and membrane‐associated states, with the latter enabling its anion‐conducting activity [42]. Although CLIC1 has not yet been investigated in pain models before, accumulating evidence has indicated that it possesses remarkable functional diversity, with several studies emphasizing roles that are either independent of or ancillary to its function as an independent chloride channel. For instance, in microglia, CLIC1 regulates morphology and NLRP3‐dependent IL‐1β release through chloride‐independent interactions with the actin cytoskeleton [65]. Similarly, in cancers, CLIC1 often acts as a coordinator within larger ion channel networks, such as by interacting with KCTD4 to potentiate calcium influx in esophageal squamous cell carcinoma [66] or cooperating with EAG2 to regulate cell volume in medulloblastoma [67]. In contrast to its previously reported non‐canonical roles, our study demonstrates that membrane‐localized CLIC1 exerts a direct and indispensable chloride channel function in trigeminal ganglion neurons under neuropathic pain conditions. We demonstrate that nerve injury‐induced CLIC1 upregulation enhances neuronal excitability, and this pronociceptive effect is fundamentally dependent on its channel function. Indeed, this paradigm of CLIC1 operating as chloride channel finds important support in several recent studies. In macrophages, CLIC1‐dependent chloride efflux is strictly required for activating the NLRP3 inflammasome and subsequent IL‐1β release [68]. More importantly, consistent with our observations on neuronal excitability, CLIC1 interacts with ERα in hypothalamic neurons to mediate the rapid excitatory effects of 17β‐estradiol, wherein its chloride conductance directly modulates neuronal excitability [69]. Interestingly, distinct from the post‐translational activation mechanisms (e.g., agonist‐induced membrane translocation) as reported previously [70], we elucidate a transcriptional mechanism for CLIC1 upregulation—through the nerve injury‐specific loss of ZBTB18‐mediated repression.

It should be worthy noted that the specificity of this ZBTB18‐CLIC1 axis is further underscored by its absence in a model of inflammatory pain (CFA‐induced), where ZBTB18 expression remains unaltered, suggesting this pathway is a hallmark of neuropathic pain in the TG. Given that ZBTB18 is expressed in DRG neurons and that epigenetic transcriptional regulation represents an important mechanism underlying neuropathic pain development, the ZBTB18–NuRD–CLIC1 regulatory axis identified in this study may have broader pathophysiological relevance beyond the CCI‐ION model. Reanalysis of publicly available transcriptomic datasets reveals that *Zbtb18* is consistently downregulated in DRG neurons across multiple neuropathic pain models, including partial sciatic nerve ligation (PSNL, GSE100035) [16] and spared nerve injury (SNI, GSE201163) [17]. This conserved injury‐induced downregulation across distinct nerve injury paradigms and species indicates that ZBTB18 may act as a shared molecular mediator of neuropathic sensitization. Nonetheless, further validation in additional pain models will help clarify its generalizability and translational value. In addition, we acknowledge that the majority of our mechanistic experiments were performed in male rats. Nevertheless, sexual dimorphism in pain processing has been reported to be largely confined to microglial and macrophage lineages [71], whereas interventions targeting neuronal and astrocytic pathways tend to yield comparable efficacy across sexes [72]. In line with this, the predominant neuronal expression of ZBTB18 and its downstream effector CLIC1 in the TG provides a plausible explanation for the equivalent pain‐modulatory effects observed in both male and female animals in our experimental paradigm. Moreover, while all siRNA reagents used in this study underwent multi‐step efficiency screening in vitro and were validated for target protein knockdown in vivo, and our core functional conclusions were supported by orthogonal overexpression experiments, rescue assays using siRNA‐resistant transgenes were not performed to fully exclude potential off‐target effects. Future work incorporating siRNA‐resistant rescue constructs will further reinforce the specificity of the proposed ZBTB18‐NuRD‐CLIC1 regulatory mechanism.

In summary, our study elucidates a novel molecular mechanism underlying neuropathic pain, wherein downregulation of ZBTB18 in injured TG neurons leads to the loss of epigenetic repression at the Clic1 promoter, thereby facilitating its transcriptional activation. We demonstrate that nerve injury impairs the interaction between ZBTB18 and CHD4, disrupting the recruitment of the NuRD complex to the Clic1 promoter. This loss of chromatin repression results in increased H3K27ac accumulation and enhanced RNA polymerase II occupancy, driving robust Clic1 transcription. The subsequent upregulation of CLIC1 protein and enhanced chloride channel activity promote neuronal hyperexcitability, ultimately contributing to the development of trigeminal neuropathic pain. Notably, restoring ZBTB18 expression in the TG alleviated neuropathic pain without affecting basal or inflammatory pain, highlighting the specificity of this pathway. These findings open new avenues for neuropathic pain management through the targeted regulation of chromatin structure at pain‐related genes, presenting a range of potential therapeutic strategies.

## 4 Materials and Methods

### 4.1 Animal Models

All animal experiments in this study were performed in compliance with the National Institutes of Health (NIH) Guidelines for Animal Research and ethical standards established by the International Association for the Study of Pain (IASP). The experimental protocols were approved by the Animal Care and Use Committee of Soochow University. Adult Sprague–Dawley rats (male and female, 8–10 weeks old) were housed five per cage with soft bedding in temperature‐ and humidity‐controlled conditions under a 12 h light/dark cycle, with food and water available ad libitum. Unless otherwise specified, male rats were used throughout the study. All animals were acclimated to the housing environment for at least 1 week prior to any surgical procedures. All experimental procedures were performed in strict adherence to the principles for minimizing animal suffering and utilizing the minimum number necessary. Trigeminal neuropathic pain was modeled via inducing unilateral chronic constriction injury of the infraorbital nerve (CCI‐ION) using an intraoral approach, as previously described [33, 73, 74]. Briefly, isoflurane‐anesthetized rats received a ∼1 cm incision along the gingivobuccal margin starting at the first molar. The left ION was surgically isolated near the infraorbital foramen using glass rods. Two 4‐0 silk sutures were loosely tied around the nerve with a spacing of 3–4 mm. Sham‐operated animals underwent identical anesthesia and surgical exposure, but without nerve ligation. For inflammatory pain modeling, complete Freund's adjuvant (CFA; 50 µl) was injected into the left buccal pad under brief isoflurane anesthesia. Control animals received an equal volume of saline administered in the same manner [75].

### 4.2 Behavioral Tests

All behavioral assessments were conducted by experimenters blinded to the experimental conditions. Testing was performed at baseline (2 days prior to surgery) and at specified time points after CCI‐ION surgery. As previously described in our established protocols [73, 74, 75], mechanical sensitivity in the orofacial region was assessed using von Frey filaments (Ugo Basile). The left vibrissal pad was stimulated with filaments of increasing force, ranging from 2 g to 15 g. A cutoff value of 15 g was implemented to prevent tissue damage and because this force consistently elicited a head withdrawal response in preliminary tests. In each testing session, every filament was applied three times consecutively, with each application maintained for 2–3 s. A positive nociceptive response was defined according to the criteria originally established by Vos et al. [76]. As the occurrence of at least one of the following behaviors: (i) brisk head withdrawal accompanied by subsequent face‐washing strokes directed to the stimulated area (≥3 strokes), (ii) attack behavior including biting or grabbing of the filament, or (iii) escape behavior characterized by withdrawal from the stimulus and adoption of a crouching position near the cage wall. The minimal von Frey filament force eliciting a positive response was defined as the mechanical escape threshold. Heat sensitivity, expressed as head withdrawal latency, was tested by radiant heat using the Hargreaves apparatus (Ugo Basile) as previously described [74]. Animals were individually placed in Plexiglas chambers and acclimated before testing. A focused radiant heat beam was applied to the ipsilateral vibrissal pad, and stimulation was terminated automatically upon head withdrawal. Head withdrawal latency was defined as the time from stimulus onset to withdrawal response. Five consecutive trials were performed at 5 min intervals, and a cutoff latency of 18 s was used to prevent tissue damage. The radiant heat intensity was adjusted to yield a baseline latency of 8–12 s.

### 4.3 Drug Application and Intra‐TG Injection

Intra‐TG injections were performed according to established protocols [73, 74]. All substances were administered slowly in a 5 µl volume, with the needle remaining in place for 10 min before withdrawal to prevent backflow. Chemically modified siRNAs targeting ZBTB18, CHD4, and CLIC1 (GenePharm, Shanghai), along with their scrambled control siRNA (NC‐siRNA), were labeled with 6‐carboxyfluorescein (6‐FAM) and reconstituted in RNase‐free water. These siRNAs were administered via intra‐TG injection once daily for two consecutive days. The specific siRNA sequences used are provided in Table S1. DNA fragments containing the ZBTB18 binding site were cloned into the pMD19‐T vector to generate decoy DNA, with the empty vector serving as a control. The sequence fidelity of all constructed plasmids was confirmed by DNA sequencing. Decoy DNA was diluted in RNase‐free water to a final concentration of 0.75 µg/µL. Neuron‐specific lentiviral vectors under the human synapsin (hSyn) promoter, including lenti‐hSyn‐ZBTB18‐up (ZBTB18‐up), lenti‐hSyn‐ZBTB18‐down (ZBTB18‐down), lenti‐hSyn‐CLIC1‐up (CLIC1‐up), lenti‐hSyn‐CLIC1‐down (CLIC1‐down), and their corresponding negative controls (NC‐up or NC‐down), were obtained from GenePharma (Shanghai). All vectors carried the eGFP reporter gene and had a minimum titer of 1 × 10^9^ TU/mL. GSK503, a potent inhibitor of enhancer of zeste homolog 2 (EZH2), the enzyme responsible for catalyzing H3K27me3, was purchased from MedChemExpress (MCE) and administered directly into the TG at 5 nmol [33]. Entinostat, a selective class I HDAC inhibitor targeting HDAC1, HDAC2 and HDAC3, was purchased from MCE, dissolved in DMSO, and administered at 30 nmol/d [77]. The CLIC chloride channel inhibitor indanyloxyacetic acid‐94 (IAA‐94) was purchased from Sigma‐Aldrich [69, 78] and dissolved in dimethyl sulfoxide (DMSO), and was administered via intraperitoneal injection with DMSO or IAA‐94 (50 mg/kg body weight).

### 4.4 Immunoblot Analysis

Immunoblotting was performed following established protocols [33, 73, 74]. Rats were euthanized with an overdose of sodium pentobarbital (150 mg/kg, i.p.) and decapitated, after which TG tissues were rapidly dissected for total protein extraction and quantified using the Pierce BCA Protein Assay Kit (Thermo Fisher Scientific). Briefly, protein samples (30 µg per lane) were separated by SDS‒PAGE and transferred onto polyvinylidene fluoride (PVDF) membranes (Millipore). After blocking with 5% nonfat milk, membranes were incubated with the following primary antibodies: ZBTB18 (rabbit, 1:2000, Proteintech), CLIC1 (rabbit, 1:500, Proteintech), CHD4 (rabbit, 1:2000, Cell Signaling Technology), HDAC1 (rabbit, 1:5000, Proteintech), HDAC2 (rabbit, 1:5000, Proteintech), MTA1 (rabbit, 1:2000, Proteintech), RBAP48 (rabbit, 1:2000, Proteintech), Flag (rabbit, 1:10 000, Proteintech), GST (rabbit, 1:10 000, Proteintech), CLIC4 (mouse, 1:100, Santa Cruz), ACTIN (mouse, 1:10 000, Proteintech), and GAPDH (mouse, 1:10 000, Proteintech). Membranes were then incubated with HRP‐conjugated secondary antibodies: goat anti‐rabbit IgG (1:8000, R&D Systems) or goat anti‐mouse IgG (1:8000, R&D Systems). For sequential immunoblotting, membranes were stripped with Western Blot Stripping Buffer (Thermo Fisher Scientific), washed with TBST, reblocked, and reprobed with the indicated antibodies. Protein bands were visualized using Pierce ECL Western Blotting Substrate (Thermo Fisher) and imaged with the ChemiDoc XRS system (Bio‐Rad Laboratories). Band intensities were quantified using Quantity One software.

### 4.5 Immunofluorescence Staining

Immunostaining was performed according to previously established methods [74, 79, 80]. Briefly, fixed trigeminal ganglia (TGs) were embedded in OCT compound (Leica) and sectioned at 10 µm thickness using a cryostat (Leica CM1950). After washing, sections underwent permeabilized and blocked in PBS supplemented with 0.25% Triton X‐100 and 5% goat serum. A subsequent overnight incubation was carried out using primary antibodies including: ZBTB18 (rabbit, 1:200, Proteintech), NF200 (mouse, 1:600, Abcam), IB4 (mouse, 1:400, Sigma), CGRP (mouse, 1:600, Abcam), GS (mouse, 1:500, Abcam), NeuN (mouse, 1:500, Cell Signaling Technology), CLIC1 (mouse, 1:100, Santa Cruz), CHD4 (mouse, 1:200, Proteintech), HDAC1 (mouse, 1:1000, Proteintech), HDAC2 (rabbit, 1:400, Proteintech), and CLIC1 (rabbit, 1:200, Proteintech). Following primary antibody incubation, sections were treated with appropriate fluorescent‐conjugated secondary antibodies. Finally, sections were mounted with antifade mounting medium containing DAPI and visualized under an upright fluorescence microscope (Nikon 104C). Fluorescence images were captured using a cooled CCD camera (CoolSNAP HQ2, Photometrics).

### 4.6 Real‐time Quantitative PCR (RT‒qPCR)

Rats were euthanized with an overdose of sodium pentobarbital (150 mg/kg, i.p.) and decapitated, after which TG tissues were rapidly dissected and total RNA was extracted using RNAiso Plus reagent (Takara). RNA quality and concentration were assessed with a NanoDrop spectrophotometer (Thermo Fisher Scientific). cDNA was synthesized from purified total RNA using the PrimeScript RT Reagent Kit (Takara). Quantitative PCR was performed on a LightCycler 96 System (ROCHE) with SYBR Green qPCR Master Mix (Takara). The thermal cycling protocol consisted of initial denaturation at 94°C for 5 min, followed by 40 cycles of 95°C for 15 s and 60°C for 60 s. Relative gene expression levels were normalized to GAPDH and calculated using the comparative 2^(‐ΔΔCq) method. All primer sequences used in this study are listed in Table S2.

### 4.7 Bisulfite Sequencing

Rats were euthanized 14 days after sham or CCI‐ION surgery, after which ipsilateral TG tissues were collected and genomic DNA was extracted using a genomic DNA extraction kit (TIANGEN). Sodium bisulfite conversion and recovery of genomic DNA were performed using the EpiTect Bisulfite Kit (Qiagen) according to the manufacturer's instructions. CpG island fragments within the Zbtb18 promoter were amplified by PCR using three pairs of bisulfite PCR primers listed in Table S2. PCR products were purified using an agarose gel DNA recovery kit (TIANGEN), and the purified target DNA fragments were ligated into the pMD18‐T vector for sequencing. For each rat, 10 colonies were randomly selected from each amplified fragment for sequencing. The resulting clone sequence files were analyzed using QUMA: QUantification tool for Methylation Analysis.

### 4.8 RNA Sequencing

RNA sequencing (RNA‐seq) was performed with three biological replicates per group in two independent comparisons: ZBTB18‐siRNA versus NC‐siRNA, and ZBTB18‐Decoy versus NC‐Decoy. Total RNA extracted from trigeminal ganglia was processed for transcriptome analysis by Ribobio (Guangzhou, China). Following RNA extraction, mRNA was enriched using poly‐T oligo‐attached magnetic beads. Strand‐specific RNA libraries were constructed with the NEBNext Ultra RNA Library Prep Kit (Illumina). Equimolar amounts of library pools were sequenced in 150 bp paired‐end mode on an Illumina NovaSeq 6000 platform. Raw sequencing reads were quality‐controlled with FastQC, followed by removal of low‐quality sequences and adapter trimming. The resulting clean reads were aligned to the Rattus norvegicus reference genome (Rnor_6.0) using HISAT2. Differential gene expression analysis was performed with DESeq2 based on read counts.

### 4.9 Chromatin Immunoprecipitation‐qPCR and ChIP‐sequencing

Chromatin immunoprecipitation was performed using the SimpleChIP Plus Enzymatic Kit (Cell Signaling Technology). Briefly, TG tissues were cross‐linked with 1.5% formaldehyde in PBS containing 0.5% protease inhibitor cocktail, followed by glycine quenching. After mechanical homogenization, nuclei were isolated by centrifugation. Genomic DNA was digested into 150–500 bp fragments using micrococcal nuclease in ChIP buffer supplemented with protease inhibitors. The lysates were sonicated and centrifuged, and 2% of the supernatant was reserved as input DNA. The remaining lysate was incubated with 2 µg of the following antibodies: anti‐ZBTB18 (Proteintech), anti‐H3K9me2 (Abcam), anti‐H3K27me3 (Abcam), anti‐CHD4 (Cell Signaling Technology), anti‐H3K27ac (Abcam), and anti‐RNA polymerase II (Abcam). Normal IgG (Cell Signaling Technology) was used as a negative control. Immunoprecipitated complexes were captured using ChIP‐grade protein G magnetic beads, washed, and eluted in ChIP elution buffer (0.1 M NaHCO_3_, 1% SDS). After reverse cross‐linking with proteinase K, DNA was purified using the kit's affinity columns. Enrichment was assessed by qPCR with primers listed in Table S2, normalized to input DNA, and verified by electrophoresis on 2% agarose gels (Sigma‒Aldrich). For ChIP‐seq, library construction and bioinformatic analysis were supported by Seqhealth Technology Co., Ltd. (Wuhan, China). Libraries were quality‐checked by electrophoresis, quantified with Qubit 2.0, and sequenced on the DNBSEQ‐T7 platform (MGI Tech) in PE150 mode. Raw reads were processed with Trimmomatic (v.0.36) to remove low‐quality and adapter‐contaminated sequences. Clean reads were aligned to the Rnor_6.0_101 reference genome using STAR (v.2.5.3a). Read distribution was analyzed with RSeQC (v.2.6), and peaks were called using MACS2 (v.2.1.1). Peak annotation and distribution were performed with bedtools (v.2.25.0). Differential binding peaks were identified by Fisher's exact test, motif analysis was conducted with HOMER (v.4.10), and genome‐wide visualization was generated using IGV (v.2.4.16).

### 4.10 Co‐Immunoprecipitation and Mass Spectrometry

Co‐IP was performed using the Classic IP/Co‐IP Kit (Thermo Fisher Scientific) according to the manufacturer's protocol. For Flag‐CoIP assays, PC12 cells were transiently transfected with FLAG‐tagged ZBTB18 constructs (full‐length WT, ΔLinker, ΔBTB, and ΔZF mutants) or empty vector control (Sangon Biotech). Trigeminal ganglion tissues or transfected PC12 cells were lysed, with 10% of the lysate reserved as input control. The remaining lysates were incubated overnight with 5 µg of the following antibodies: anti‐ZBTB18 (Proteintech), anti‐CHD4 (Cell Signaling Technology), anti‐FLAG (Proteintech), or rabbit IgG as a negative control (Thermo Fisher Scientific). Immunocomplexes were captured using protein A/G magnetic beads, washed three times with lysis buffer, and eluted. Eluates were denatured in 5× sample buffer at 95°C for 5 min and analyzed by immunoblotting. For detecting ZBTB18, CHD4, HDAC1, HDAC2, MTA1, and RBAP48, membranes were processed as described above, except that Clean‐Blot IP Detection Reagent (HRP) (Thermo Fisher Scientific) was used as the secondary antibody. For mass spectrometry analysis, immunoprecipitated samples were separated by SDS‐PAGE and visualized by silver staining. The gel was fixed, sensitized, stained with silver solution, and developed before immediate termination with acetic acid. Alternatively, co‐immunoprecipitated proteins were digested with trypsin, and the resulting peptides were acidified, desalted using Strata‐X SPE columns, and separated by ultra‐high performance liquid chromatography. LC‐eluted peptides were analyzed by an Orbitrap Exploris 480 mass spectrometer equipped with a nano‐electrospray ion source (spray voltage: 2.3 kV). Full MS scans (400–1200 m/z) were acquired at 60 000 resolution, and MS/MS scans were performed at 15 000 resolutions with a first fixed mass of 110 m/z. MS/MS data were processed using MaxQuant (v.1.6.15.0) and searched against the Rattus_norvegicus 10116_PR_20231121 fasta database (47 943 entries) supplemented with decoy and contaminant sequences.

### 4.11 GST Pull‐Down Assay

The GST‐ZBTB18 fusion proteins were produced in E. coli BL21 cells and purified using GST 4FF affinity chromatography (Sangon Biotech) according to the manufacturer's instructions. To test for direct binding, GST‐ZBTB18 fusion protein was equilibrated in binding buffer for 20 min at ambient temperature with FLAG tagged fusion proteins, including FLAG‐CHD4, FLAG‐HDAC1, FLAG‐HDAC2, FLAG‐MTA1, and FLAG‐RBAP48, which had been expressed in PC12 cells and pre‐purified with anti‐FLAG magnetic beads (Bimake). Post‐equilibration, this mixture was combined with glutathione agarose 4B resin (GE Medical), per the manufacturer's guidelines, and subjected to a 2‐h rotation at room temperature. Following trifold washes with the binding buffer, bound proteins were eluted using SDS loading buffer. The eluted protein fractions were then analyzed on SDS‐PAGE, and then analyzed via western blotting.

### 4.12 Screening of Target siRNAs in PC12 Cells

PC12 cells were cultured in complete medium containing DMEM/F‐12 (Gibco), 10% fetal bovine serum and 1% antibiotics. All cells were maintained in a humidified incubator (Thermo Fisher Scientific) at 37°C and 5% CO_2_. After digestion with 0.25% trypsin (Sigma), the cells were resuspended in complete medium. Cells were transfected with NC‐siRNA or four independent siRNAs targeting *Zbtb18*, *Chd4* or *Clic1* using Lipofectamine 3000 (Invitrogen). The siRNA sequences used are provided in Table S1. Total RNA was extracted 48 h after transfection for qPCR analysis to evaluate knockdown efficiency of each independent siRNA

### 4.13 Dual‐Luciferase Reporter Assay

Wild‐type and mutant *Clic1* promoter sequences were cloned into the pGL3‐basic vector. The wild‐type construct contained a 591 bp fragment (−482 to +109 bp) spanning the transcriptional start site, while mutant variants included single‐site mutations (mut‐1, mut‐2) or a double mutation (dmut) in ZBTB18 binding sites. All constructs were verified by Sanger sequencing (Azenta Life Sciences). PC12 cells were cotransfected using Lipofectamine 3000 (Invitrogen) with 500 ng of pGL3‐promoter construct, 500 ng of ZBTB18 overexpression plasmid, and 100 ng of pRL‐TK *Renilla* luciferase internal control. Firefly and *Renilla* luciferase activities were quantified sequentially 48 h post‐transfection utilizing the Dual‐Glo Assay System (Promega) on a TD‐20/20 luminometer (Turner BioSystems). Firefly luciferase activity was normalized to *Renilla* activity, and results are expressed relative to the wild‐type control group.

### 4.14 Dissociation of TG Neurons

Trigeminal ganglion (TG) neurons were acutely isolated from rats according to established protocols [75]. Briefly, TGs were rapidly dissected from rats following decapitation under deep anesthesia. The ganglia were then cleared of connective tissues in ice‐cold, oxygenated DDB solution and subjected to sequential enzymatic digestion with 2.5 mg/ml collagenase D (Roche) at 37°C for 50 min followed by 2 mg/ml trypsin (Sigma) at 37°C for 15 min. Following dissociation by gentle trituration with flame‐polished Pasteur pipettes, single neurons were plated onto Matrigel‐coated glass coverslips (Merck) and incubated overnight at 37°C. The culture medium was replaced with fresh complete medium the following day, and all electrophysiological recordings were completed within 24 h post‐plating.

### 4.15 Patch‐Clamp Electrophysiology

Retrogradely labeled small‐diameter trigeminal ganglion (TG) neurons (soma diameter <30 µm) were identified for electrophysiological recording four days after the subcutaneous administration of the retrograde tracer DiI (20 mg/ml, Thermo Fisher) into the rat vibrissal pad. Whole‐cell patch‐clamp recordings were acquired from TG neurons at room temperature (22–24°C) following established procedures [75, 79, 80]. Signals were amplified using a MultiClamp 700B amplifier (Molecular Devices), low‐pass filtered at 2 kHz, and digitized at 10 kHz with a Digidata 1440A system (Molecular Devices). Data acquisition and analysis were performed using pCLAMP 10 and Clampfit 10.2 software (Molecular Devices), respectively. Patch pipettes (3–5 MΩ) were filled with appropriate intracellular solutions. CLIC channel currents were recorded using the following solutions (in mM). The bath solution contained: 130 NaCl, 5.5 KCl, 24 HEPES, 1 MgCl_2_, 0.5 CaCl_2_, and 5 d‐glucose; pH was adjusted to 7.4 with NaOH. The pipette solution contained (in mM): 128 N‐methyl‐glucamine‐Cl, 5 KCl, 2.5 CaCl_2_, 1 MgCl_2_, 10 HEPES, 5 glucose, and 10 tetraethylammonium‐Cl; pH was adjusted to 7.3–7.4 with HCl [69]. Under voltage‐clamp conditions with the membrane potential held at −60 mV, total chloride channel currents were recorded in the presence of channel inhibitors including 1 µM TTX (voltage‐gated sodium channels), 10 µM TTA‐P2 (T‐type calcium channels), 50 µM nifedipine (L‐type calcium channels), and 5 mM 4‐aminopyridine (potassium channels) [79, 81]. Currents were evoked using a 1‐s voltage ramp from –80 mV to +80 mV. To isolate CLIC1‐mediated currents, neurons were perfused with 100 µM IAA‐94 (a chloride channel inhibitor; Sigma‐Aldrich) for 3 min. The specific CLIC channel currents (IAA‐94‐sensitive component) were determined by digital subtraction of the residual currents after IAA‐94 application from the total currents. Current‐clamp recordings were obtained with the intracellular pipette solution containing (in mM): 110 KCl, 25 HEPES, 10 NaCl, 4 Mg‐ATP, 0.3 Na‐GTP, and 2 EGTA (pH adjusted to 7.4 with KOH; 295 mOsm). The extracellular solution was composed of (in mM): 128 NaCl, 2 KCl, 2 CaCl_2_, 2 MgCl_2_, 25 HEPES, and 30 glucose (pH adjusted to 7.4 with NaOH; 305 mOsm).

### 4.16 Data Analysis and Statistics

All data are presented as mean ± SEM. For data collection and statistical analyses, we employed Microsoft Excel, Clampfit 10.2 (Molecular Devices), and GraphPad Prism 8.0. Sample sizes were not predetermined via statistical methods; rats were randomly assigned without any formal randomization algorithm. For comparisons between two groups, a two‐tailed unpaired Student's t‐test was employed. Multiple group comparisons were analyzed by one‐way ANOVA, while datasets involving multiple time points were assessed using two‐way ANOVA. When ANOVA indicated significant differences, *post hoc* Bonferroni testing was applied for further analysis. A *p*‐value of less than 0.05 was considered statistically significant. For statistical analysis, measured values deviating from the group mean by more than three standard deviations were defined as extreme outliers and excluded from the final analysis.

## Author Contributions

J.T., Y.Z., and M.X. as the corresponding authors conceived the project and supervised all experiments. S.W., Y.T., Z.H., Y.Z., Y.S., D.J., W.L., G.C. and F.J. designed and performed the experiments. Y.S. and F.J. were responsible for material acquisition. S.W., Y.Z., Z.H. and Y.T. analyzed the data. The manuscript was drafted by J.T., Y.Z., M.X., and S.W. All authors read and approved the final manuscript.

## Funding

This work was supported by grants from the Brain Science and Brain‐like Intelligence Technology‐National Science and Technology Major Project (No. 2025ZD0214900), the National Science Foundation for Distinguished Young Scholars of China (No. 82425019), the National Key Research and Development Program of China (No. 2024YFC2510103), the National Natural Science Foundation of China (Nos. 82271245, 82371218 and 82402888), the MOE Key Laboratory of Geriatric Diseases and Immunology (No. JYN202403), the Postgraduate Research & Practice Innovation Program of Jiangsu Province (No. KYCX25_3477), the Jiangsu Market Supervision Administration Science and Technology Project (No. KJ2022038), the Jiangsu Key Laboratory of Drug Discovery and Translational Research for Brain Diseases (No. JKLDDTRBD), and the Priority Academic Program Development of Jiangsu Higher Education Institutions (No. PAPDJHEI).

## Conflicts of Interest

The authors declare no conflicts of interest.

## Supporting information




**Supporting File**: advs77364‐sup‐0001‐SuppMat.pdf.

## Data Availability

The data that support the findings of this study are openly available in NCBI Gene Expression Omnibus at https://www.ncbi.nlm.nih.gov/gds/?term=, reference number GSE224814, GSE310186, GSE310187, GSE337672.
