# Supplementary material for: ZBTB18 Dysfunction Promotes Neuropathic Pain via CHD4‐based Epigenetic Disinhibition of CLIC1 Channels in Sensory Neurons
Source: Adv Sci (Weinh). 2026 Aug 27;13(63):e77364. doi: 10.1002/advs.77364 (PMC13521199; doi:10.1002/advs.77364)
Supplement: Supplementary file 1 [file ADVS-13-e77364-s001.pdf]

Supporting Information

ZBTB18 dysfunction promotes neuropathic pain via CHD4-based epigenetic disinhibition of CLIC1 channels in sensory neurons

Shoupeng Wang [1,#], Zitong Huang [1,#], Yu Tao [1,#], Yunmei Zhang [1], Yufang Sun [1,6], Dongsheng Jiang [2], Weiwei Lu [1,6], Fuhai Ji [3,6], Gang Chen [3,6], Min Xu [4,*], Yuan Zhang [5,*], Jin Tao [1,3,6,*]

[1] The First Affiliated Hospital of Soochow University, School of Basic Medical Sciences, Suzhou Medical College of Soochow University, Suzhou 215123, P.R. China;

[2] Precision Research Center for Refractory Diseases, Shanghai General Hospital, Shanghai Jiao Tong University School of Medicine, Shanghai 201620, P.R. China;

[3] Department of Anesthesiology & Department of Neurosurgery, The First Affiliated Hospital of Soochow University, Suzhou 215006, P.R. China;

[4] Department of Neurosurgery, Kunshan Hospital of Traditional Chinese Medicine, Kunshan Affiliated Hospital of Yangzhou University, Kunshan 215300, P.R. China;

[5] Clinical Research Center of Neurological Disease, Department of Geriatrics, The Second Affiliated Hospital of Soochow University, Suzhou 215004, P.R. China;

[6] Jiangsu Key Laboratory of Drug Discovery and Translational Research for Brain Diseases, Centre for Ion Channelopathy, Soochow University, Suzhou 215123, P.R. China.

[#] These authors contribute to this work equally.

Running title: ZBTB18 dysfunction promotes neuropathic pain

***To whom correspondence should be addressed:**

Dr. Jin Tao, E-mail: taoj@suda.edu.cn

Dr. Yuan Zhang, E-mail: yuanzhang@suda.edu.cn

Dr. Min Xu, E-mail: 15950155750@yzu.edu.cn

This *Supporting Information* file includes: SI Figures S1 to S28

SI Tables S1 to S2

SI Data S1 to S3

SI Figures S1 to S28

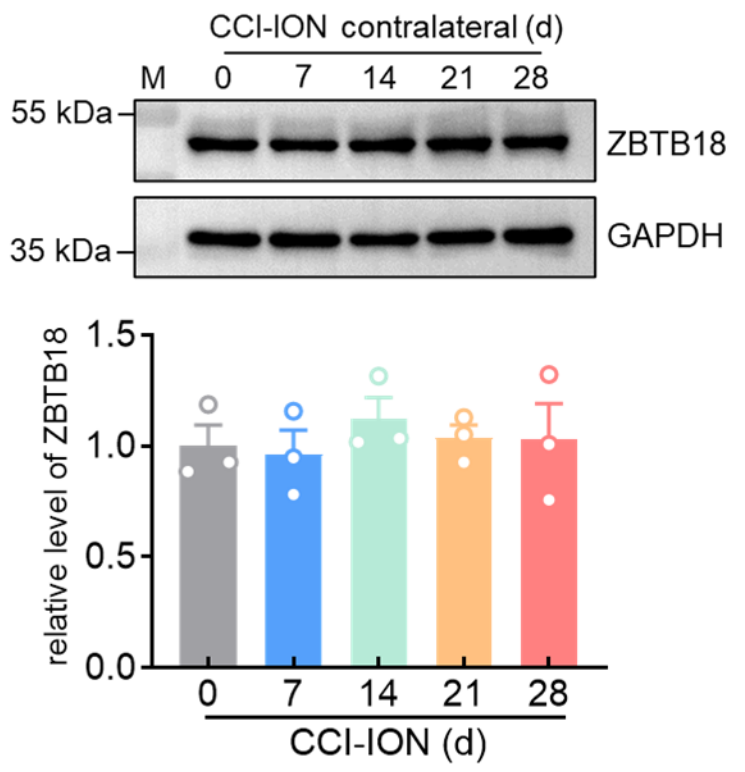

Fig. S1. Quantitative analysis shows that CCI-ION surgery did not significantly alter ZBTB18 protein levels in the contralateral TGs across the indicated time points. $p > 0.05$, compared with day 0 group, one-way ANOVA, $n = 3$ rats/time point. Shown blots are representative of three independent experiments.

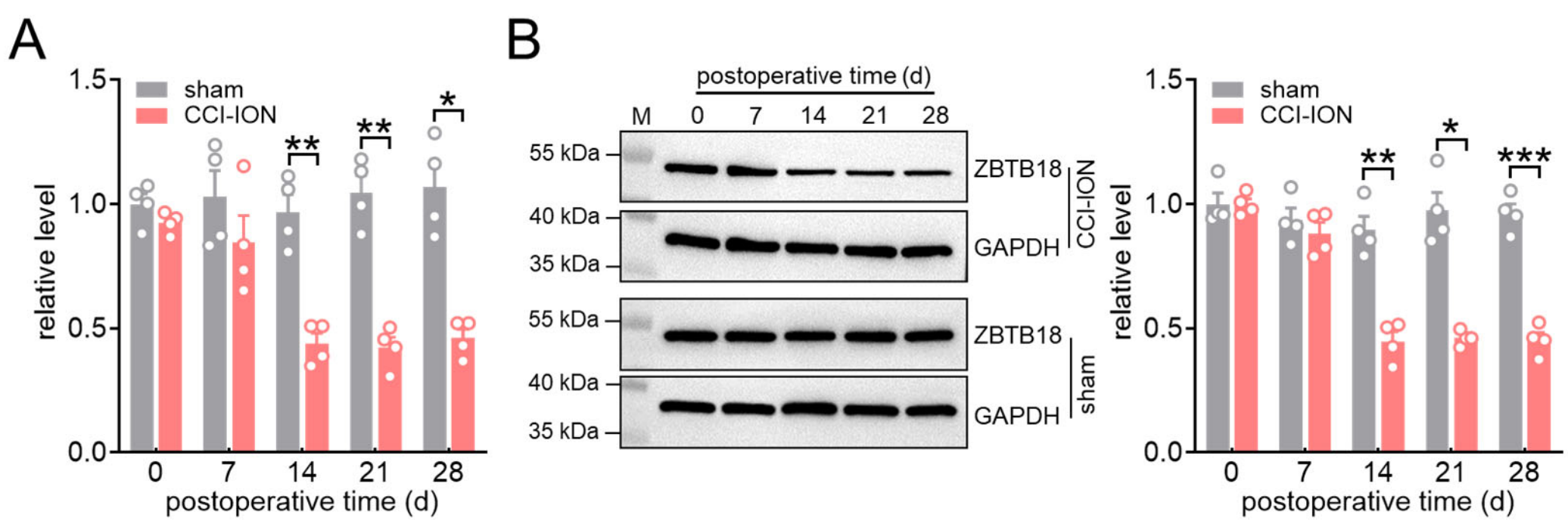

Fig. S2. mRNA and protein expression of ZBTB18 in TGs after CCI-ION in female rats. *(A)* Time-dependent changes in *Zbtb18* mRNA expression after CCI-ION in female rats. $^*p < 0.05$, $^{**}p < 0.01$ compared with corresponding sham group, two-way ANOVA, $n = 4$ rats/time point/group. *(B)* Immunoblot analysis of ZBTB18 protein expression over time. $^*p < 0.05$, $^{**}p < 0.01$, $^{***}p < 0.001$ compared with corresponding sham group, two-way ANOVA, $n = 4$ rats/time point/group. Shown blots are representative of four independent experiments.

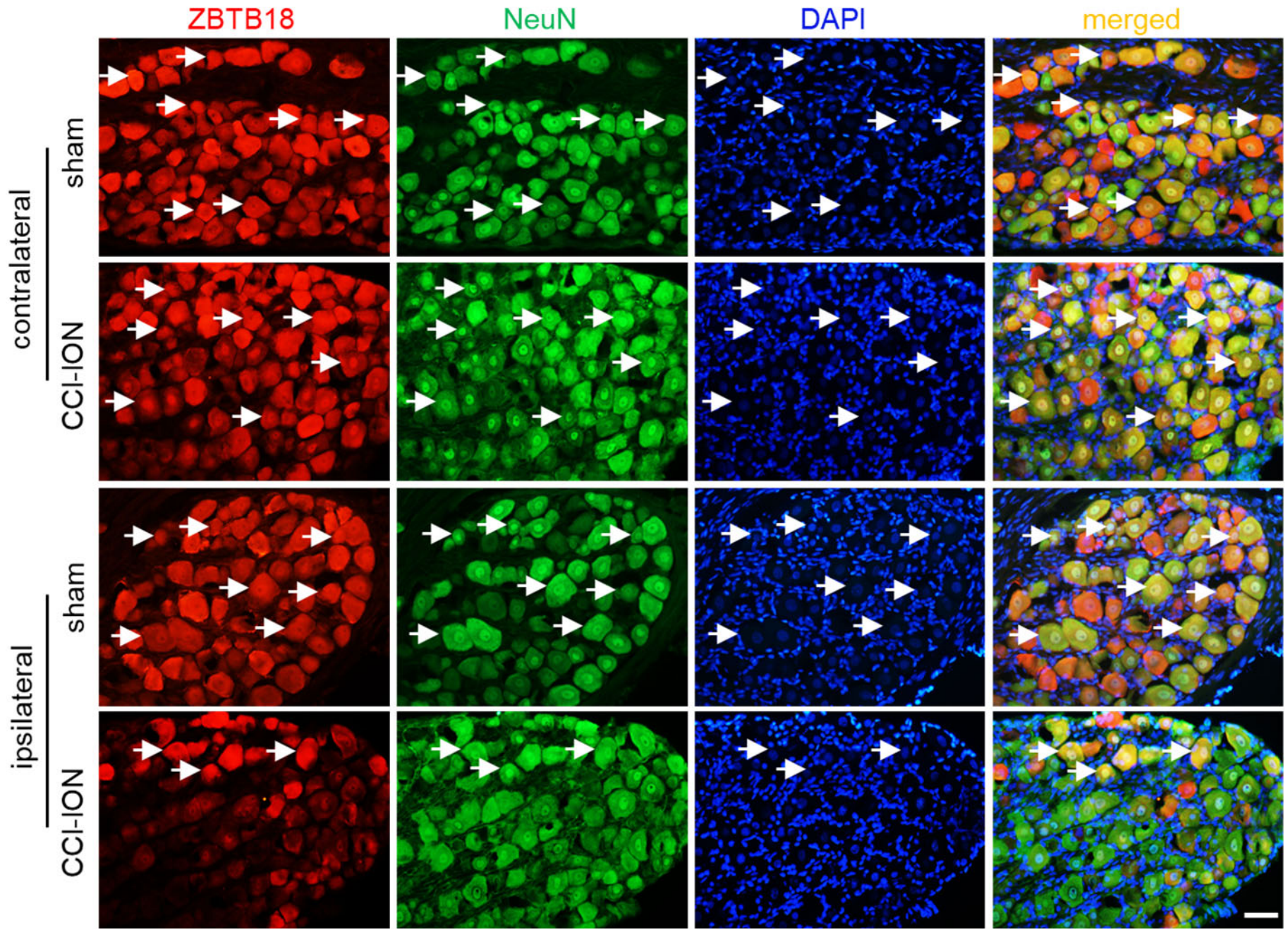

Fig. S3. Full immunofluorescence co-localization images of ZBTB18 (red) with NeuN (green) in TG neurons are included in Fig. 1L. Arrows indicate examples of positive neurons. Scale bar, 50 μm.

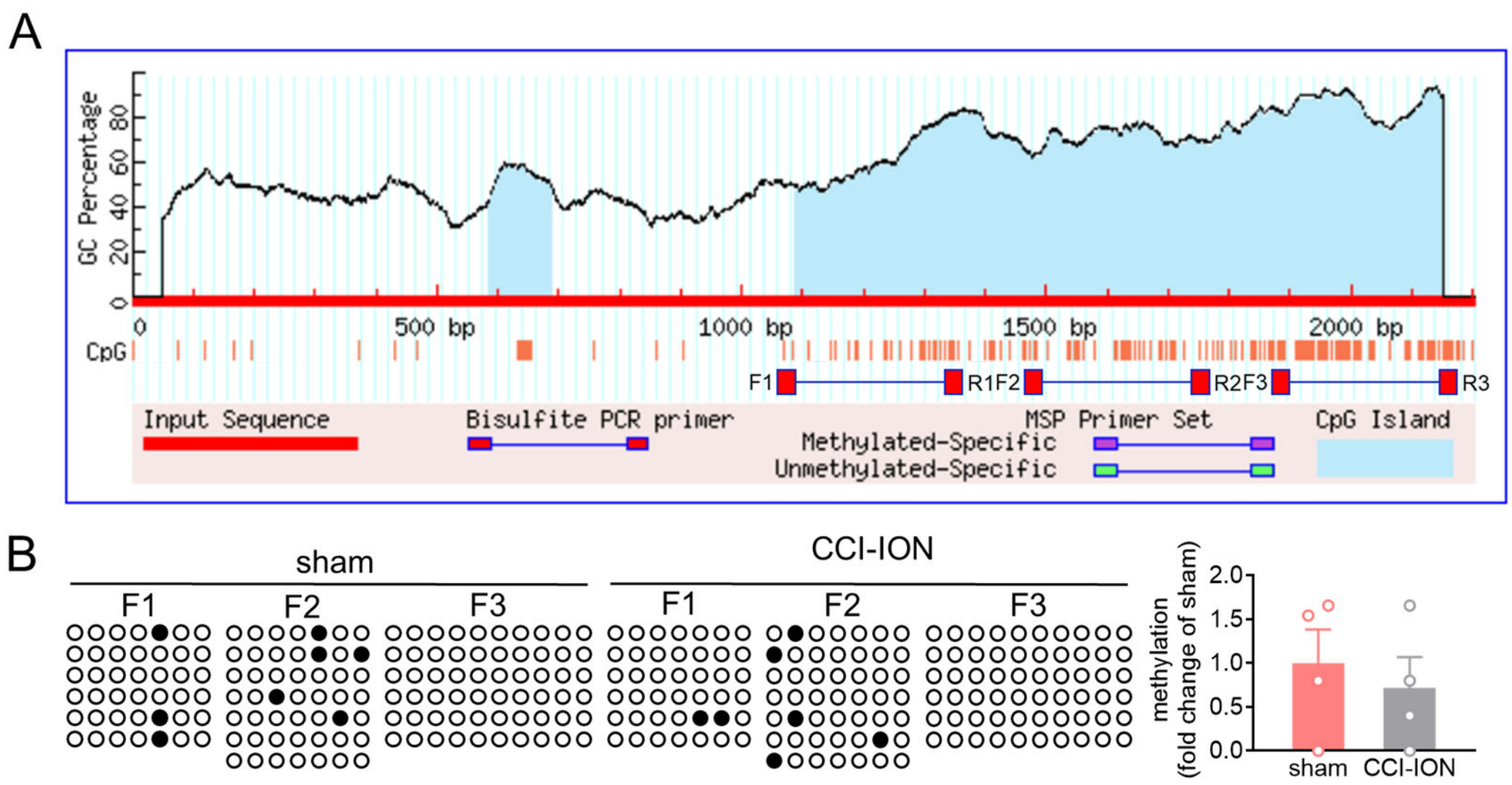

Fig. S4. Nerve injury does not alter DNA methylation status at the *Zbtb18* promoter region in TGs. *(A)* Bioinformatic prediction by MethPrimer revealed CpG island regions (blue regions) and three BSP target fragments within the *Zbtb18* promoter region. *(B)* Bisulfite sequencing PCR analysis of *Zbtb18* promoter methylation status in TGs of sham and CCI-ION rats 14 days after surgery. Open circles represent unmethylated CpG sites; filled circles represent methylated CpG sites. $p > 0.05$, compared with sham group, unpaired *t*-test, $n = 4$ rats/group.

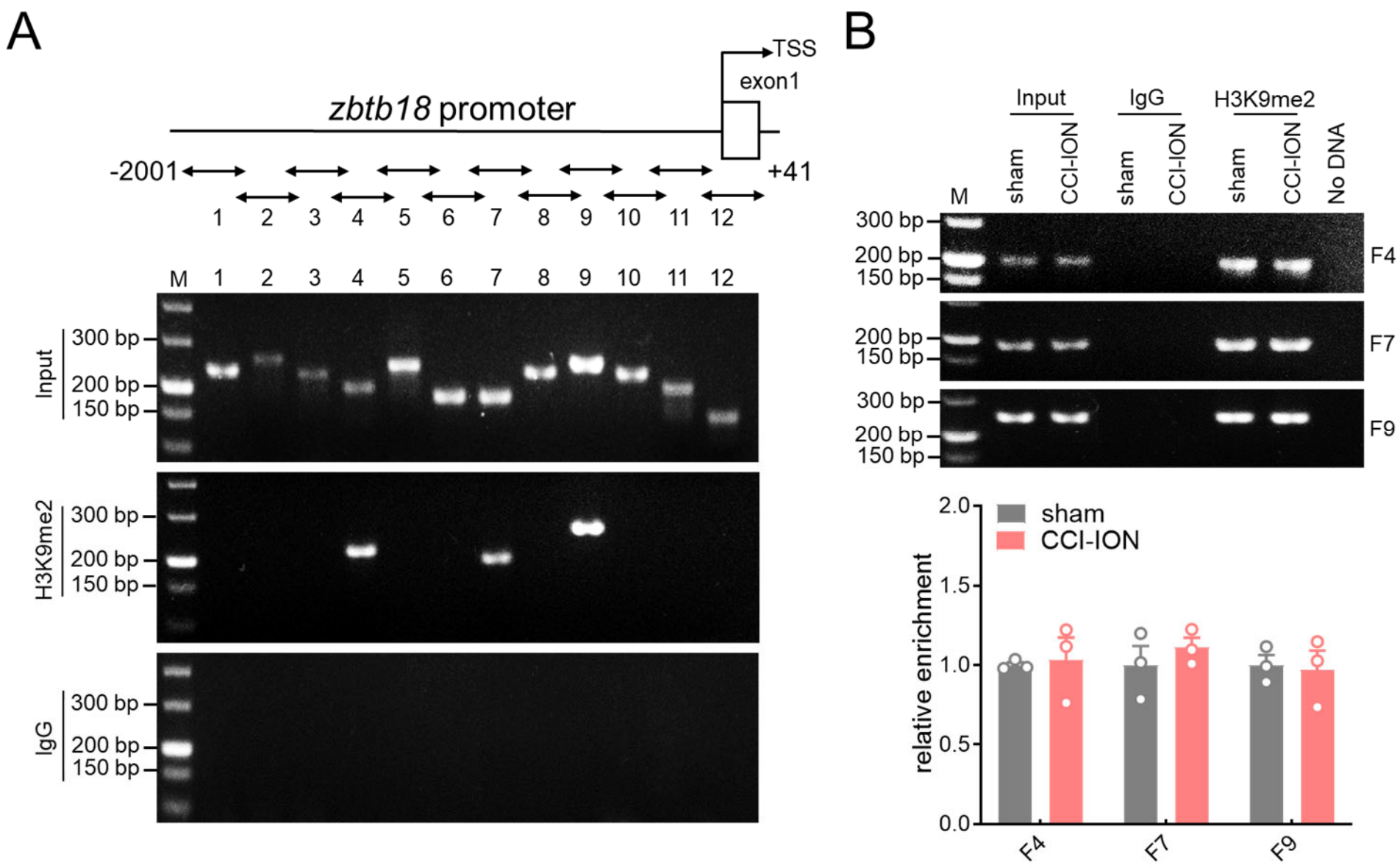

Fig. S5. Nerve injury does not alter H3K9me2 levels at the *Zbtb18* promoter region in TGs. *(A)* Schematic of *Zbtb18* promoter and exon 1 regions immunoprecipitated via an anti-H3K9me2 antibody in rat TGs. Data are representative of three independent experiments. *(B)* H3K9me2 enrichment at fragments F4, F7 and F9 remained unchanged after CCI-ION surgery. $p > 0.05$, compared with corresponding sham group, multiple t-test, $n = 12$ rats/group. Representative blots from three experiments.

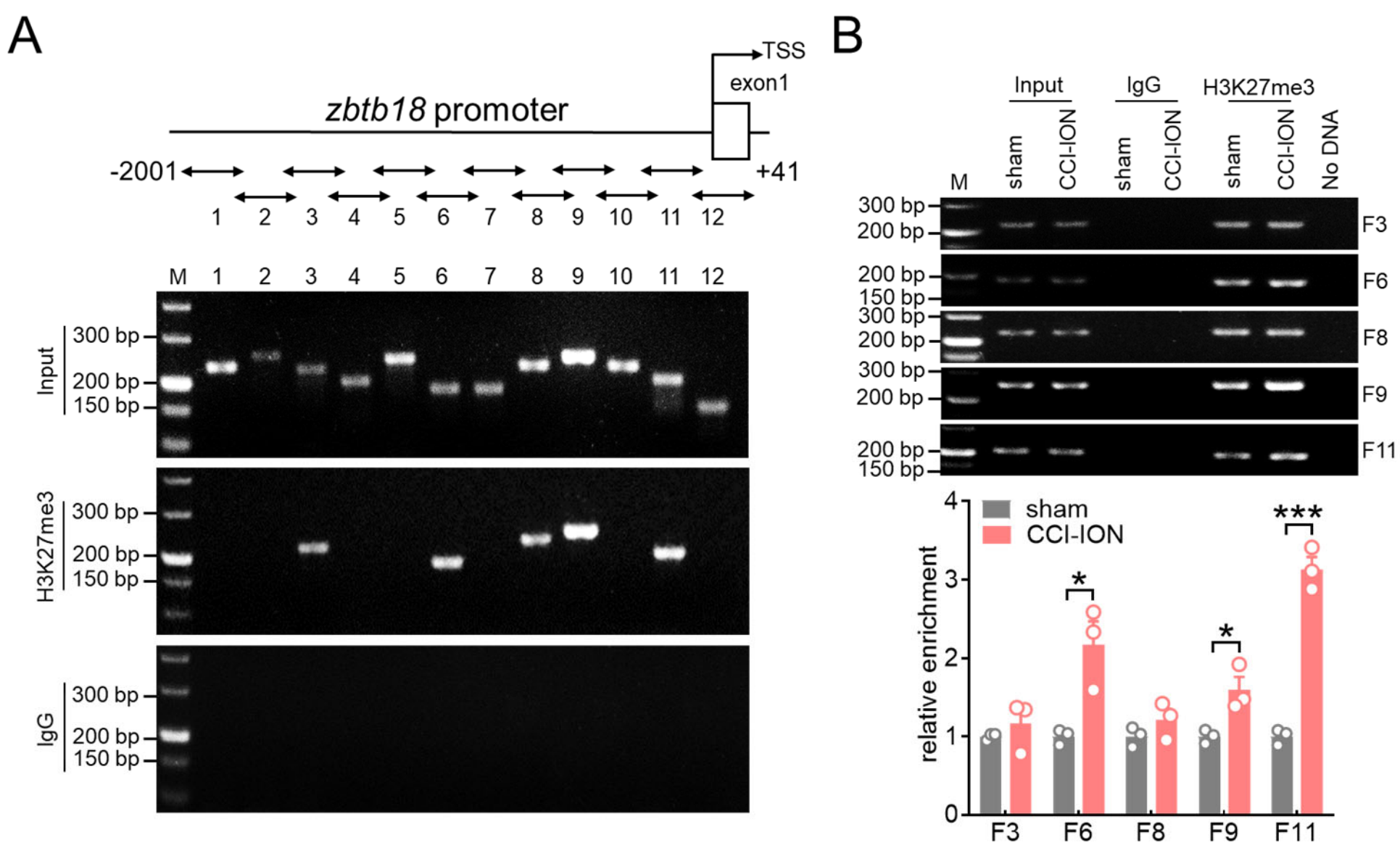

Fig. S6. Nerve injury increases H3K27me3 levels at the *Zbtb18* promoter region in TGs. *(A)* Schematic of *Zbtb18* promoter and exon 1 regions immunoprecipitated via an anti-H3K27me3 antibody in rat TGs. Data are representative of three independent experiments. *(B)* H3K27me3 enrichment at fragments F6, F9 and F11 significantly increased after CCI-ION surgery, but not at fragments F3 or F8. $^{*}p < 0.05$, $^{***}p < 0.001$, compared with corresponding sham group, multiple t-test, $n = 12$ rats/group. Representative blots from three experiments.

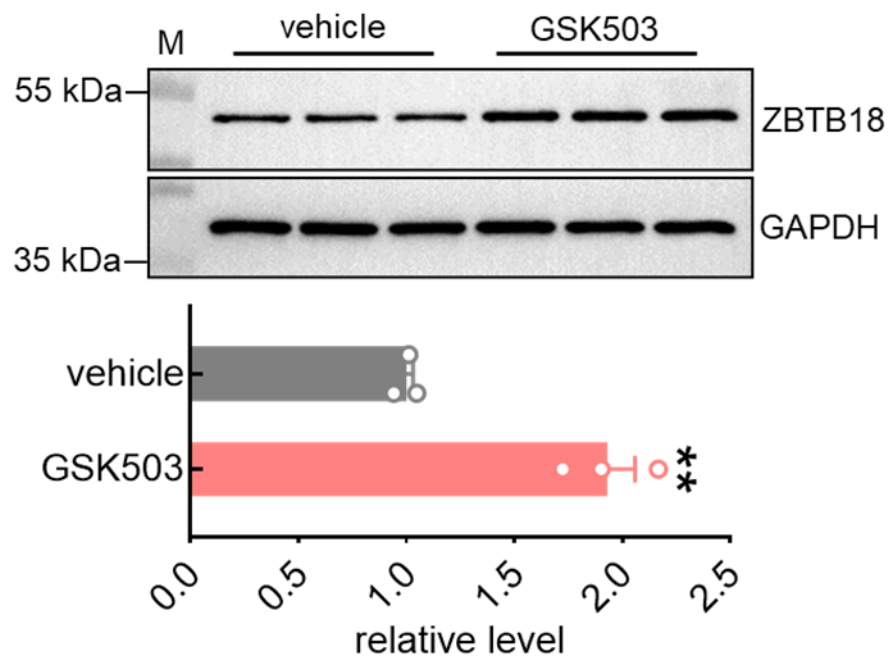

Fig. S7. Intra-TG injection of GSK503 (5 nmol) increased ZBTB18 protein expression on day 14 after CCI-ION. $^{**}p < 0.01$ compared with vehicle group, unpaired *t*-test, $n = 3$ rats/group. Shown blots are representative of three independent experiments.

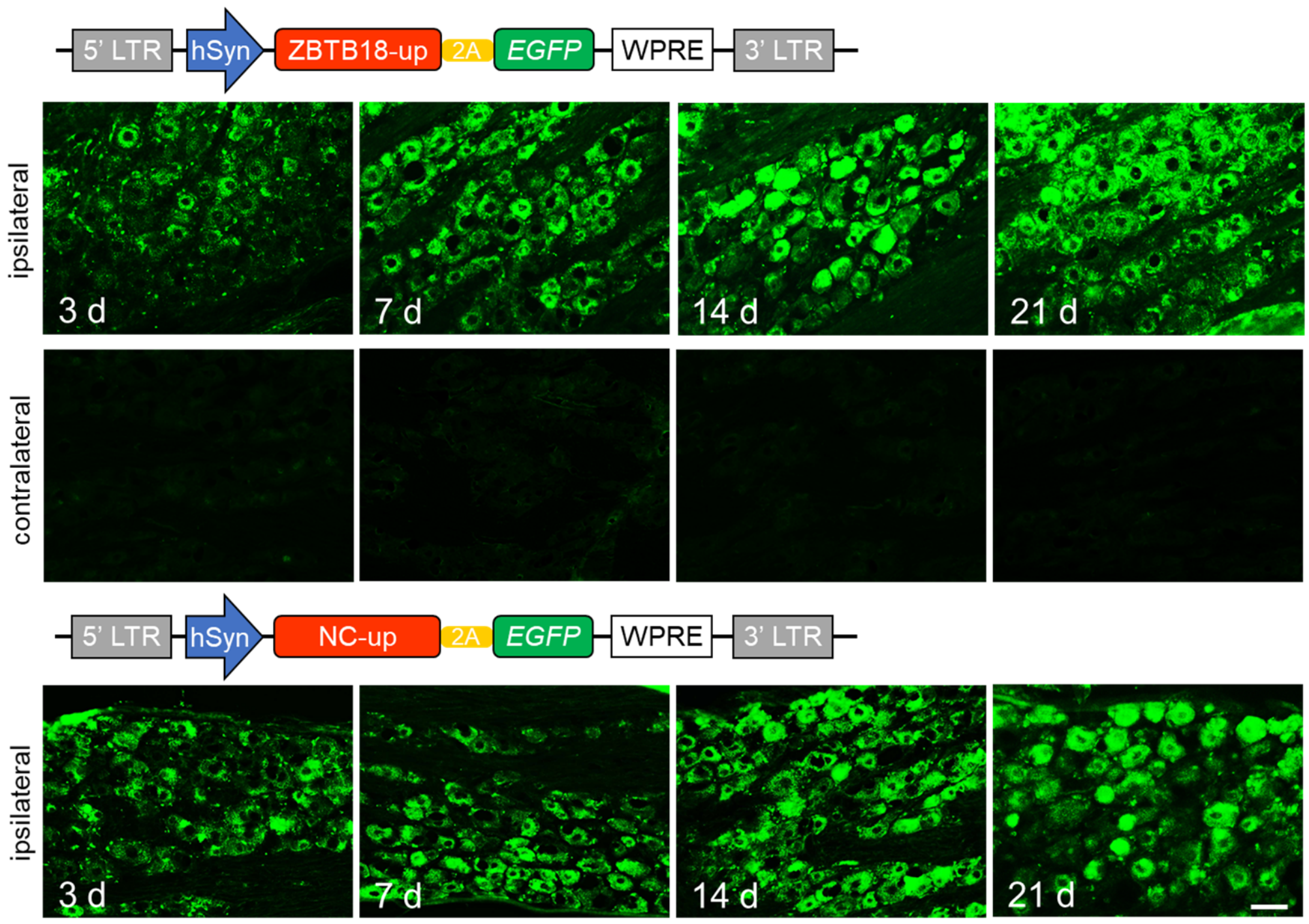

Fig. S8. Validation of lentiviral transduction efficiency in the TG of intact rats. eGFP fluorescence in ipsilateral TG sections confirms successful neuronal transduction at 3 days after intra-TG injection of lenti-hSyn-ZBTB18-up-eGFP (ZBTB18-up) or lenti-hSyn-NC-up-eGFP (NC-up), with sustained transgene expression observed up to 21 days post-injection; no eGFP fluorescence was detected in contralateral TG sections from ZBTB18-up treated rats. Data are representative of three independent experiments. Scale bar: 50 μm.

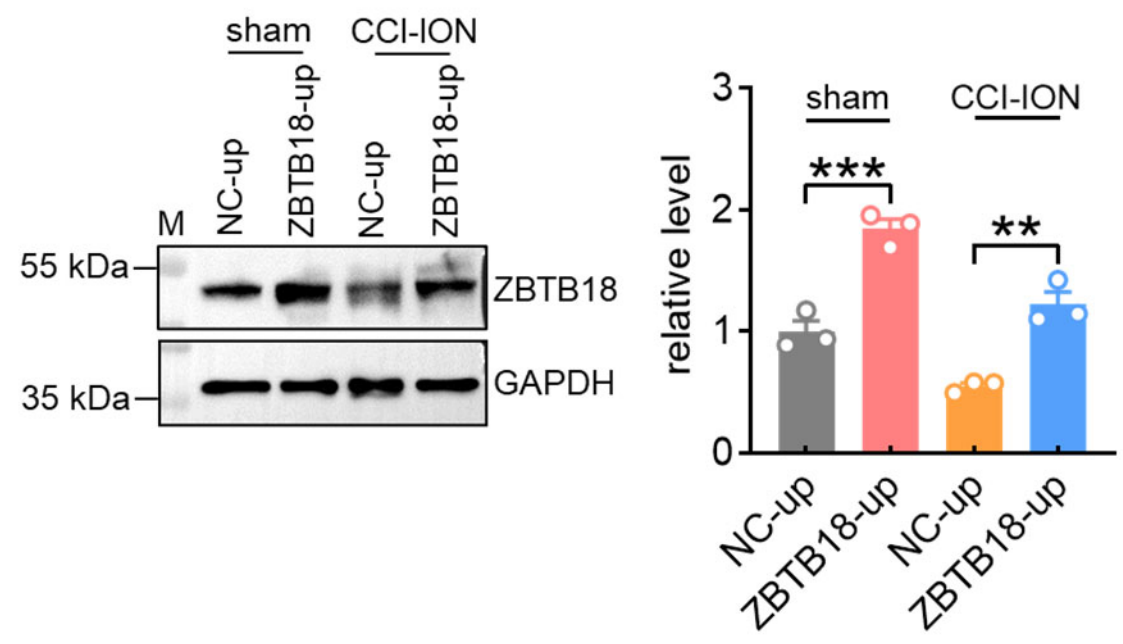

Fig. S9. ZBTB18-up prevented the downregulation of ZBTB18 protein induced by CCI-ION. **$p < 0.01$, ***$p < 0.001$ compared with corresponding NC-up group, one-way ANOVA, n = 3 rats/group. Shown blots are representative of three independent experiments.

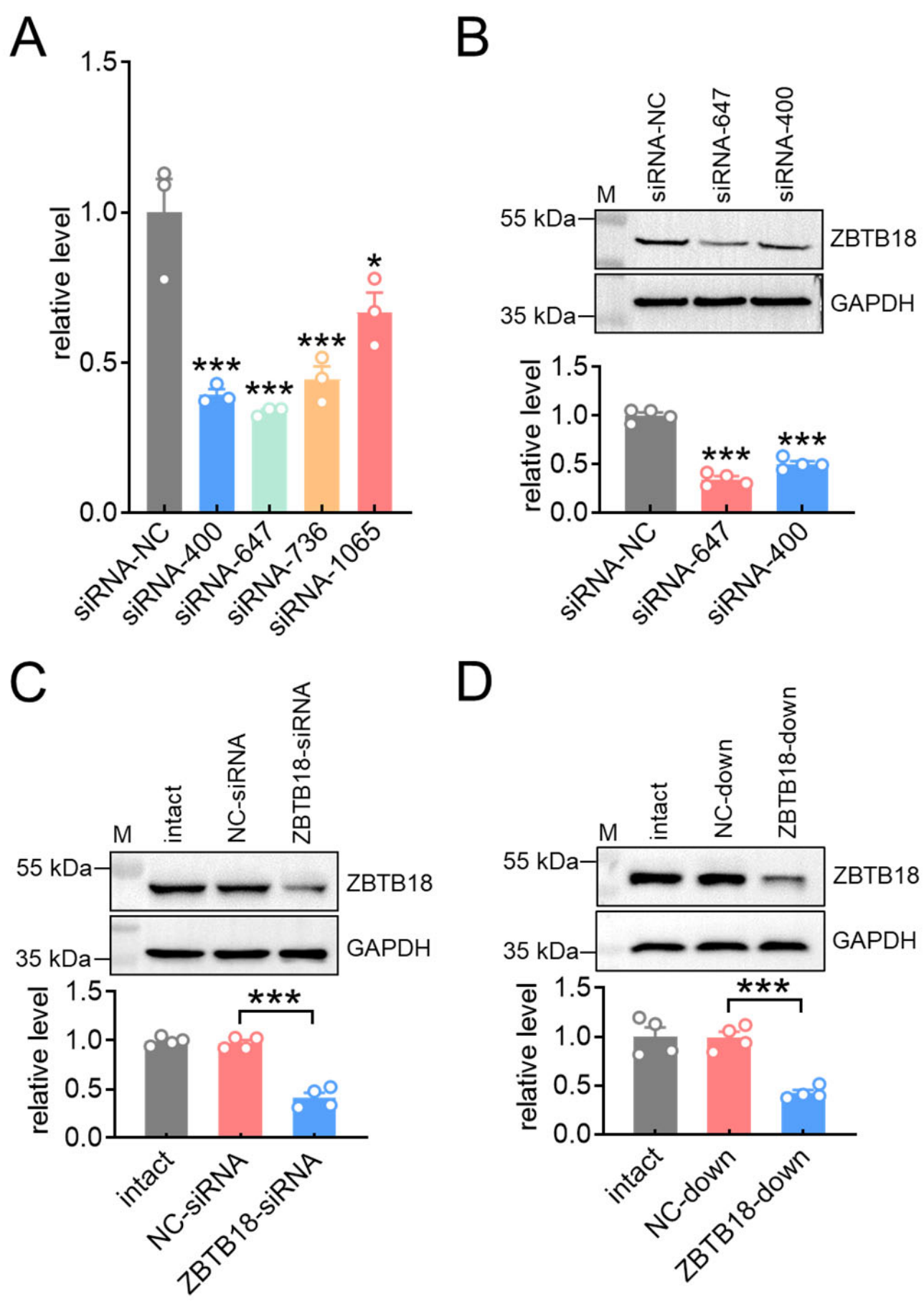

Fig. S10. Regulation of ZBTB18 expression by siRNA and shRNA. *(A) Zbtb18* mRNA levels in PC12 cells 48 h after transfection with four independent siRNAs (siRNA-400, siRNA-647, siRNA-736 and siRNA-1065). *$p < 0.05$, ***$p < 0.001$ compared with siRNA-NC group, one-way ANOVA, n = 3. *(B)* ZBTB18 protein levels after intra-TG delivery of modified siRNA-647 or siRNA-400. ***$p < 0.001$ compared with siRNA-NC group, one-way ANOVA, n = 4

rats/group. Shown blots are representative of four independent experiments. *(C)* Intra-TG delivery of ZBTB18-siRNA reduced ZBTB18 protein levels. ***$p < 0.001$ compared with NC-siRNA group, one-way ANOVA, n = 4 rats/group. Shown blots are representative of four independent experiments. *(D)* Intra-TG administration of lentiviral-mediated shRNA against ZBTB18 (ZBTB18-down) decreased the protein abundance of ZBTB18. ***$p < 0.001$ compared with NC-down group, one-way ANOVA, n = 4 rats/group. Shown blots are representative of four independent experiments.

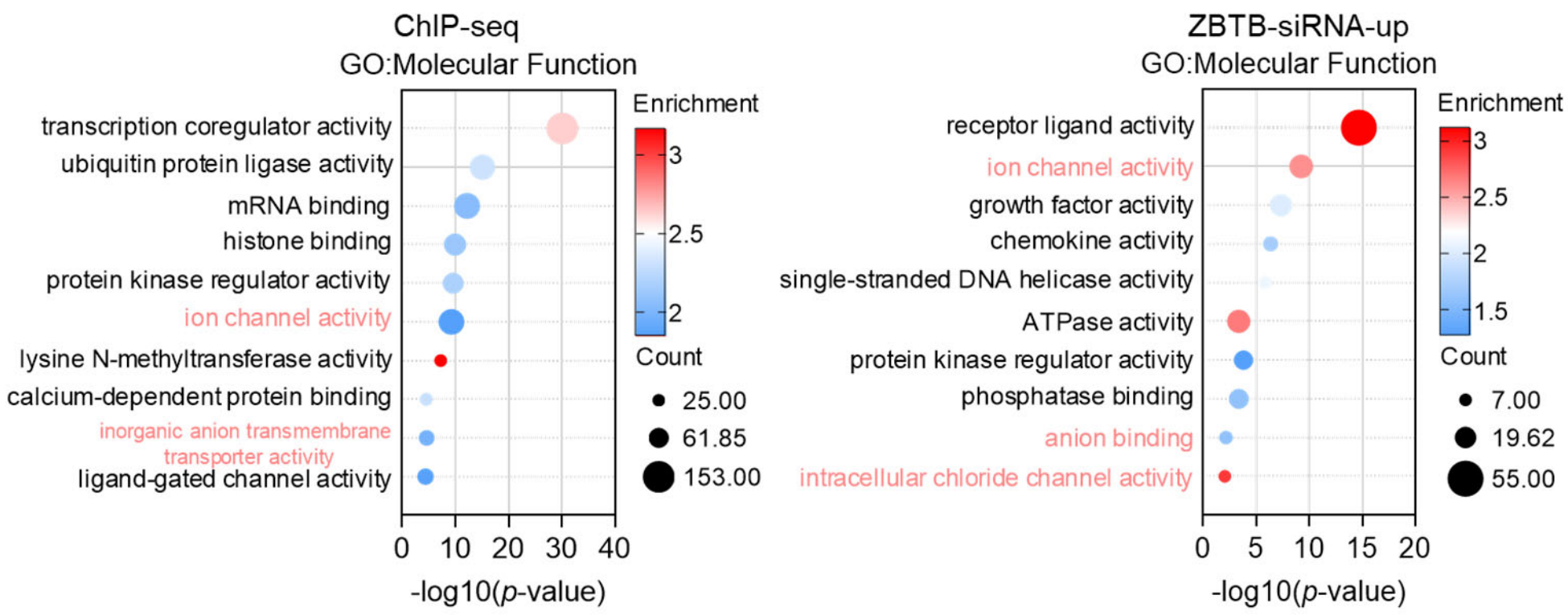

Fig. S11. Gene Ontology (GO) analysis of enriched molecular function terms for ZBTB18 ChIP-seq targets (left) and genes upregulated after ZBTB18 knockdown (right).

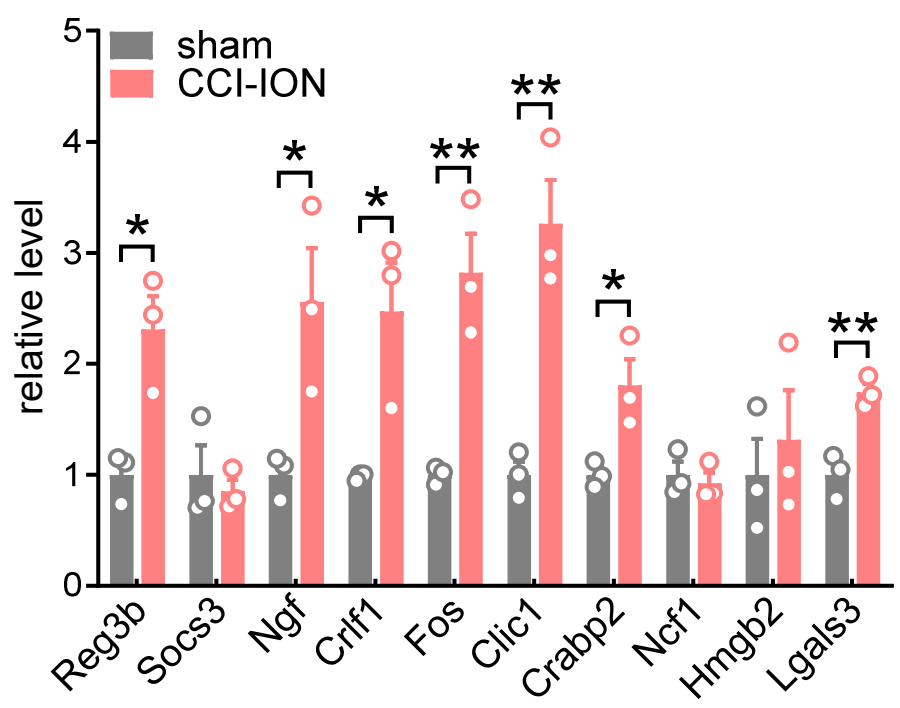

Fig. S12. qPCR validation of the top ten candidate target genes upregulated in ipsilateral TGs 14 days after CCI-ION. *$p < 0.05$, **$p < 0.01$ compared with corresponding sham group, multiple t-test, n = 3 rats/group.

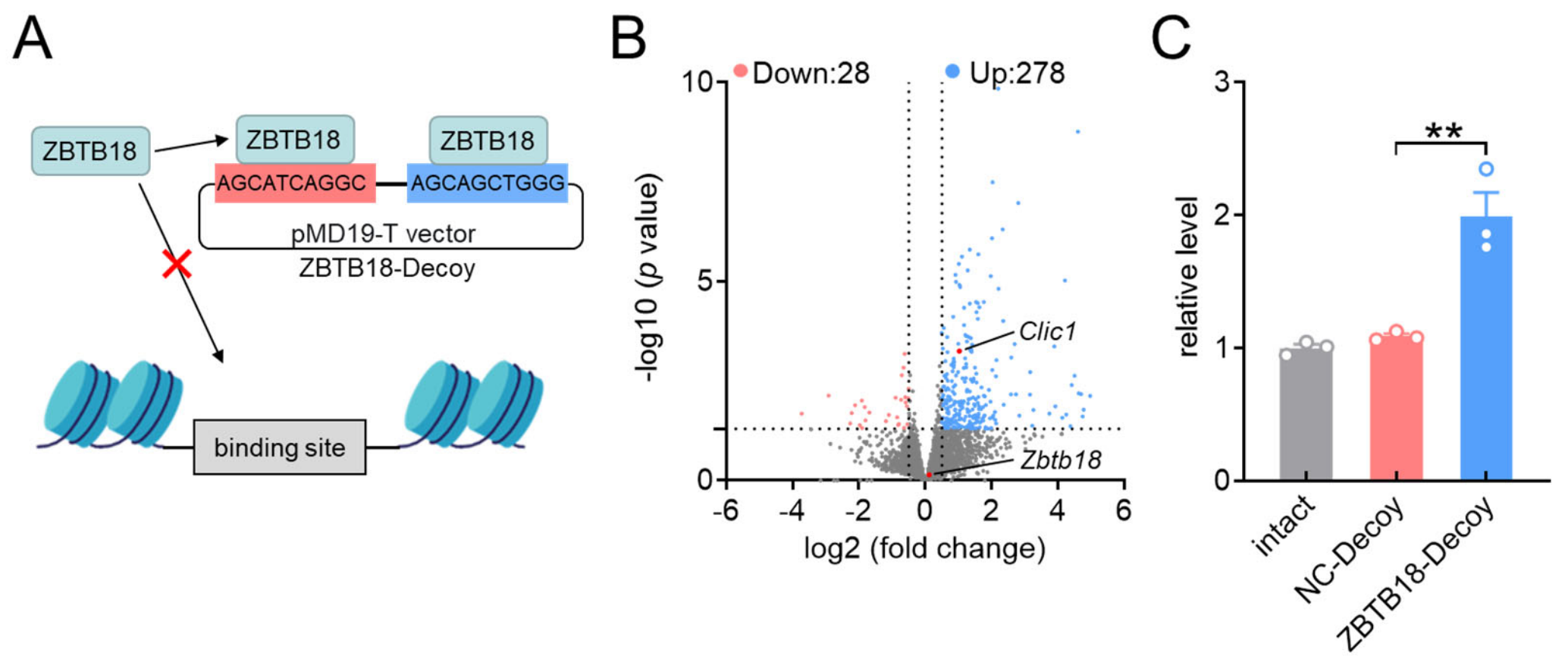

Fig. S13. Blocking ZBTB18 binding to the *Clic1* promoter enhances *Clic1* transcription. *(A)* Schematic graph showed the blockade of ZBTB18 binding to the *Clic1* promoter by decoy DNA. *(B)* Volcano plot illustrating transcripts upregulated (*blue*) and downregulated (*red*) in ZBTB18-Decoy-treated TGs compared to negative control decoy (NC-Decoy). *(C)* qPCR analysis of *Clic1* mRNA expression in TGs at 3 days after intra-TG delivery of ZBTB18-Decoy or NC-Decoy. $n = 3$ rats per group; $**p < 0.01$ versus NC-Decoy group, one-way ANOVA.

A

ZBTB18-associated peptides		
ZBTB18	6	5
Mi2β(CHD4)	17	14
HDAC1	6	6
HDAC2	3	4
MTA1	1	2
RbAP48(Rbbp4)	8	8
RbAP46(Rbbp7)	5	4

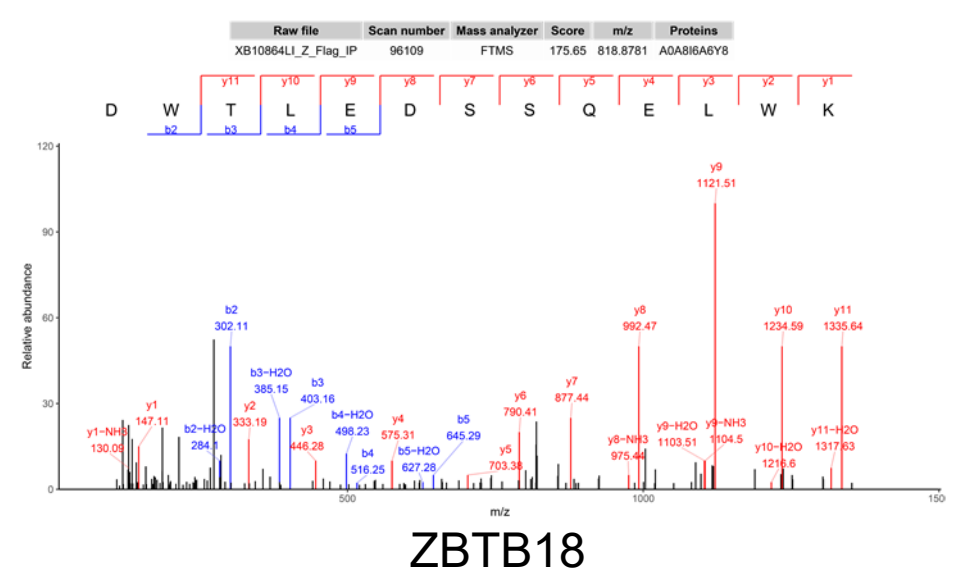

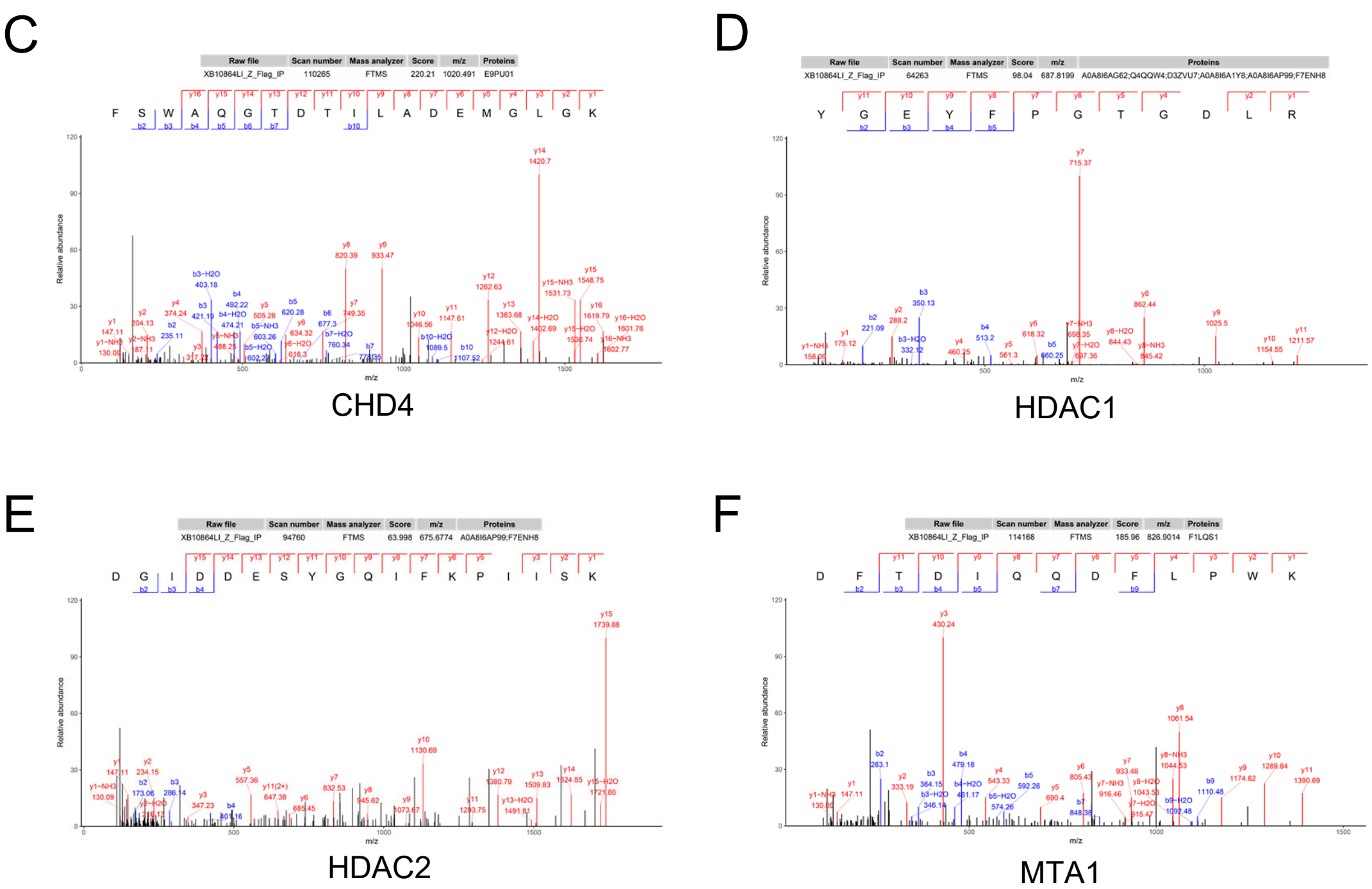

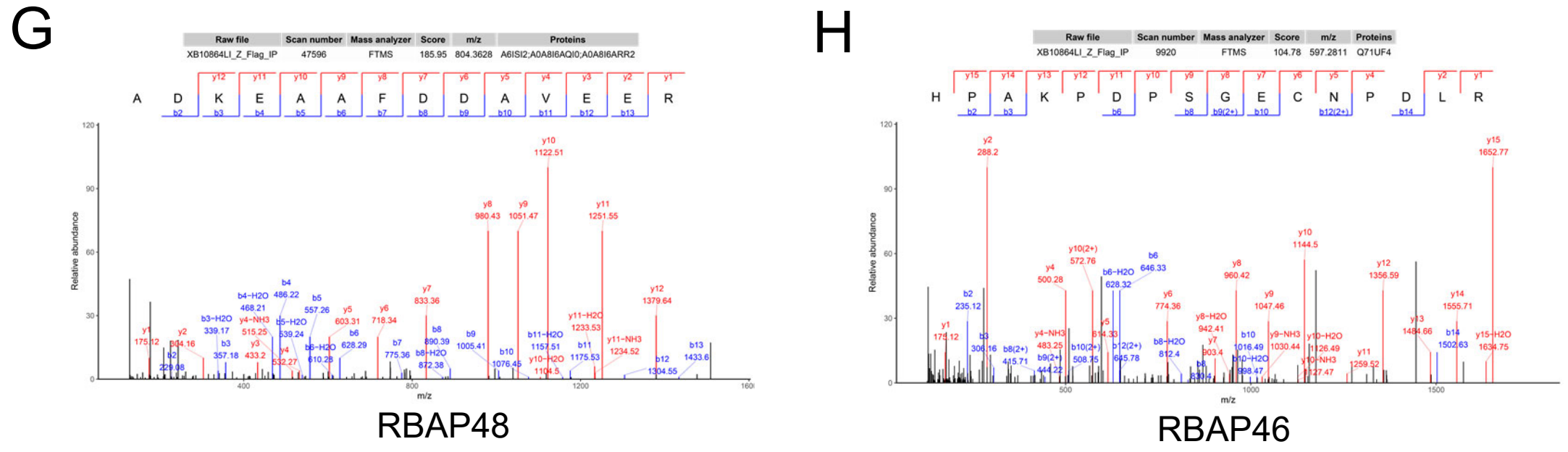

Fig. S14. Identification of ZBTB18 and NuRD complex subunits by mass spectrometry. (*A*) List of NuRD complex-related peptides associated with ZBTB18 as identified by LC-MS/MS analysis. (*B-H*) Representative MS/MS spectrograms of selected ZBTB18-interacting proteins.

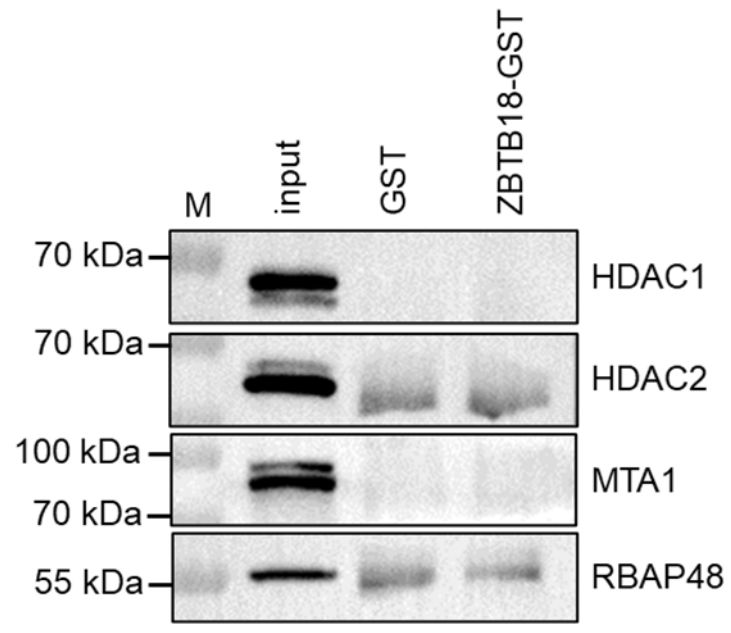

Fig. S15. Assessment of direct interactions between ZBTB18 and other NuRD complex components. GST pull-down assays demonstrate that ZBTB18 does not directly interact with HDAC1, HDAC2, MTA1, or RBAP48.

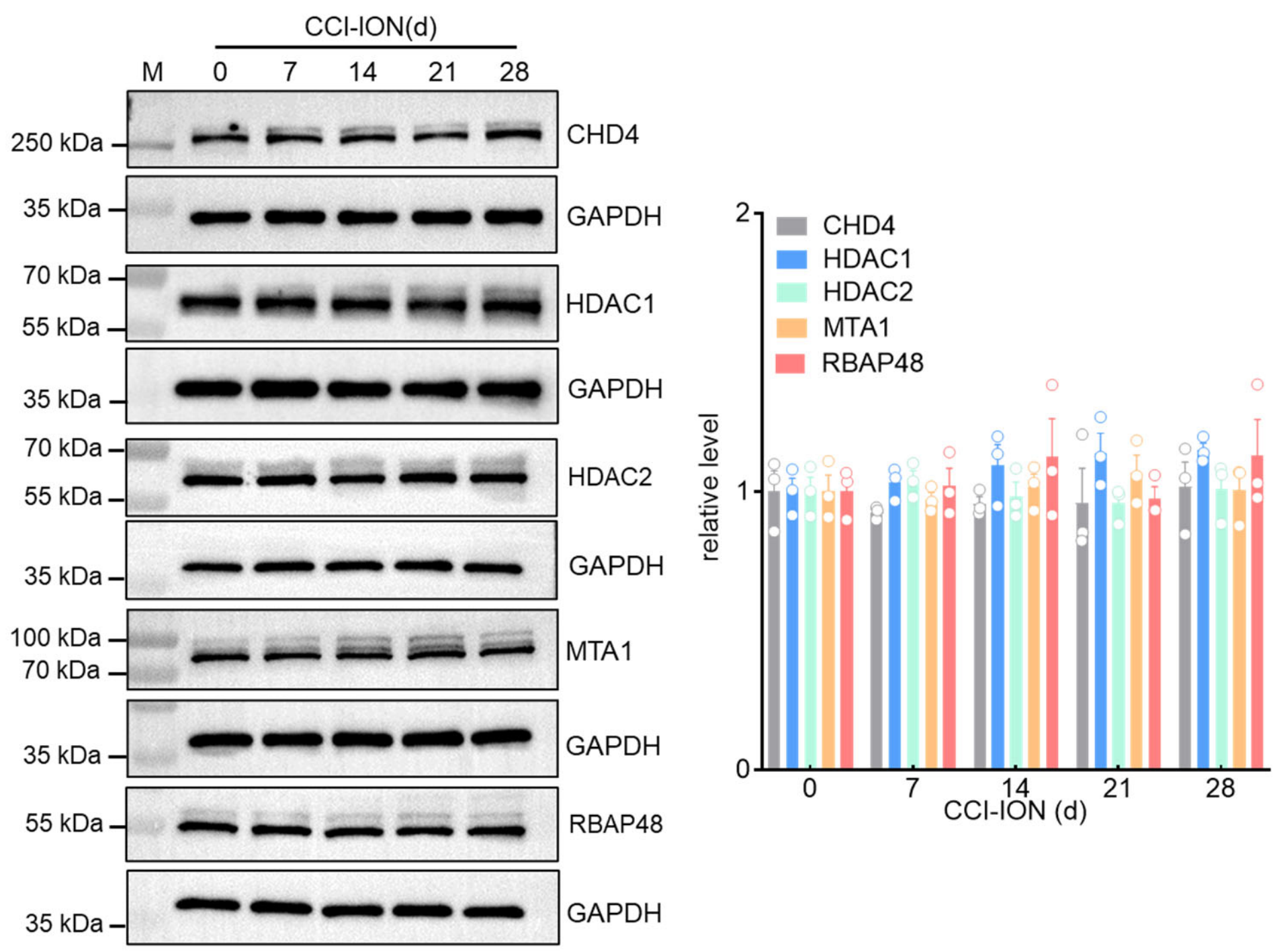

Fig. S16. Protein expression of NuRD complex subunits in trigeminal ganglia following nerve injury. Quantitative analysis of CHD4, HDAC1, HDAC2, MTA1, and RBAP48 protein levels shows no significant changes across the indicated time points after surgery. $p > 0.05$, compared with corresponding day 0 group, two-way ANOVA, $n = 3$ rats/time point/group. Shown blots are representative of three independent experiments.

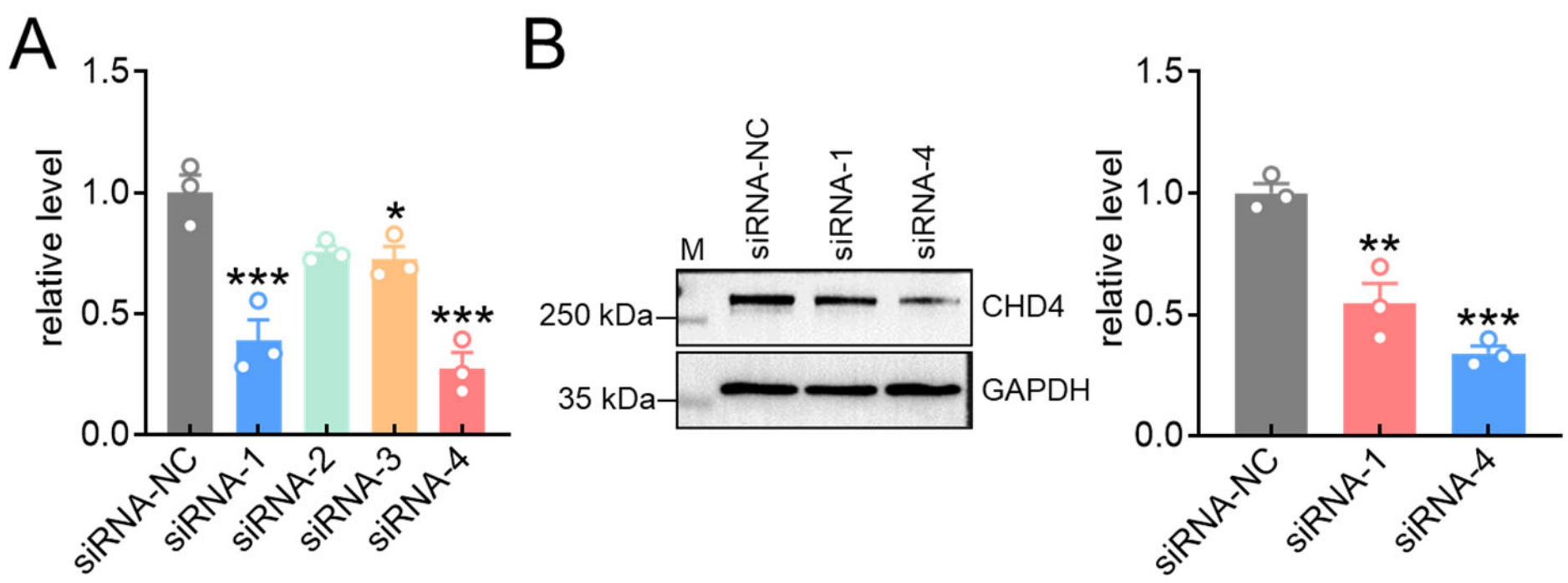

Fig. S17. Screening of siRNAs targeting CHD4 in rats. *(A)* CHD4 mRNA levels in PC12 cells 48 h after transfection with four independent siRNAs (siRNA-1, siRNA-2, siRNA-3 and siRNA-4). *$p < 0.05$, ***$p < 0.001$ compared with siRNA-NC group, one-way ANOVA, $n = 3$. *(B)* CHD4 protein levels after intra-TG delivery of modified siRNA-1 or siRNA-4. **$p < 0.01$, ***$p < 0.001$ compared with siRNA-NC group, one-way ANOVA, $n = 3$ rats/group. Shown blots are representative of three independent experiments.

ZBTB18 residue	CHD4 residue	Interaction type
ARG-18	LYS-1521	Hydrogen bond Interaction
ASP-55	GLN-888	Hydrogen bond Interaction
LYS-115	GLU-833	Hydrogen bond Interaction
GLU-163	LYS-803	Hydrogen bond Interaction
GLY-162	LYS-825	Hydrogen bond Interaction
SER-226	SER-1897	Hydrogen bond Interaction
ASP-58	LYS-832	Electrostatic interaction
ARG-60	ASP-1388	Electrostatic interaction

Fig. S18. Structural model of the ZBTB18-CHD4 complex showing detailed residue-residue interaction types at the binding interface.

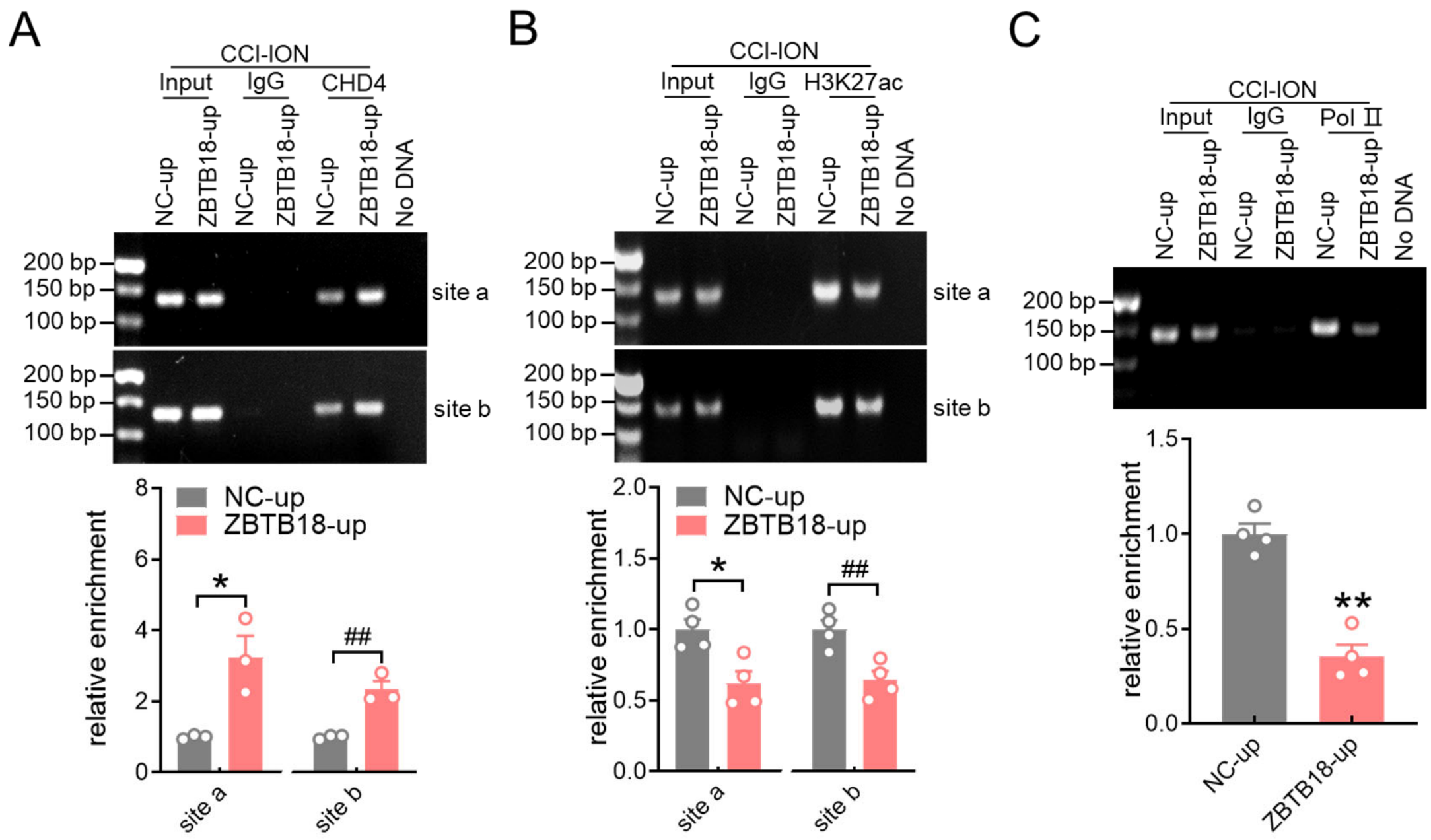

Fig. S19. Intra-TG administration of ZBTB18-up prevents CCI-ION-induced epigenetic alterations at the *Clic1* promoter and Pol II recruitment at the *Clic1* transcription start site. (*A-B*) ZBTB18-up prevented the CCI-ION-induced reduction in CHD4 occupancy (*A*) and the increase in H3K27ac enrichment (*B*) at two binding sites in the *Clic1* promoter (n = 12-16 rats per group; *$p < 0.05$ versus NC-up for site a, $^{\#\#}p < 0.01$ versus NC-up for site b, multiple t-test). (*C*) Intra-TG administration of ZBTB18-up prevented the CCI-ION-induced increase in RNA polymerase II (Pol II) enrichment at the *Clic1* transcription start site region (n = 16 rats per group; **$p < 0.01$ versus NC-up, unpaired t-test).

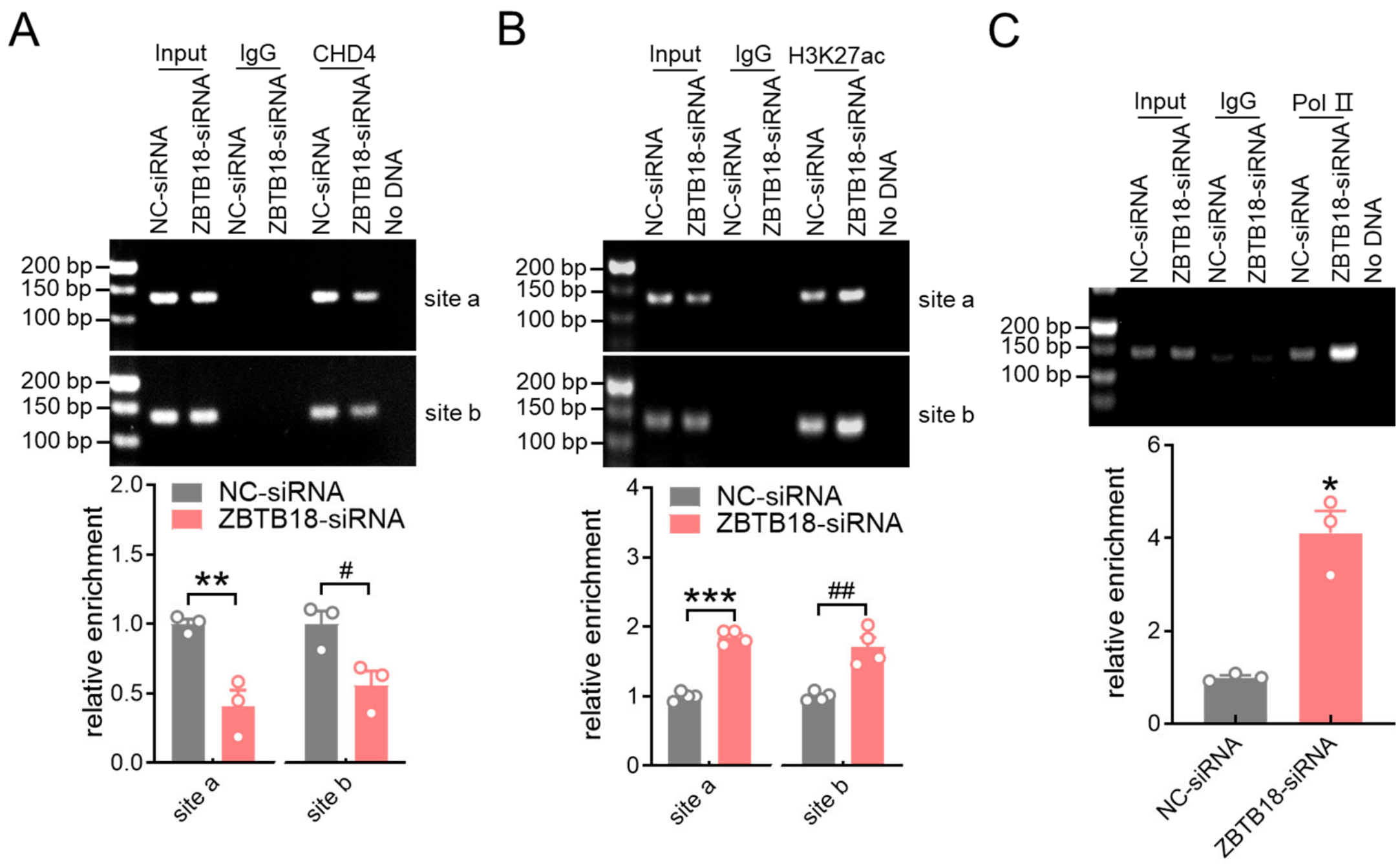

Fig. S20. ZBTB18 knockdown alters epigenetic states at the *Clic1* promoter and enhances Pol II recruitment at the *Clic1* transcription start site in intact rats. (*A-B*) Intra-TG administration of ZBTB18-siRNA significantly reduced CHD4 occupancy (*A*) and increased H3K27ac enrichment (*B*) at two binding sites in the *Clic1* promoter (n = 12-16 rats per group; **$p < 0.01$, ***$p < 0.001$ versus NC-siRNA for site a; $^{\#}p < 0.05$, $^{\#\#}p < 0.01$ versus NC-siRNA for site b; multiple t-test). (*C*) Intra-TG administration of ZBTB18-siRNA significantly increased RNA polymerase II (Pol II) enrichment at the *Clic1* transcription start site region in intact rats (n = 12 rats per group; *$p < 0.05$ versus NC-siRNA, unpaired t-test).

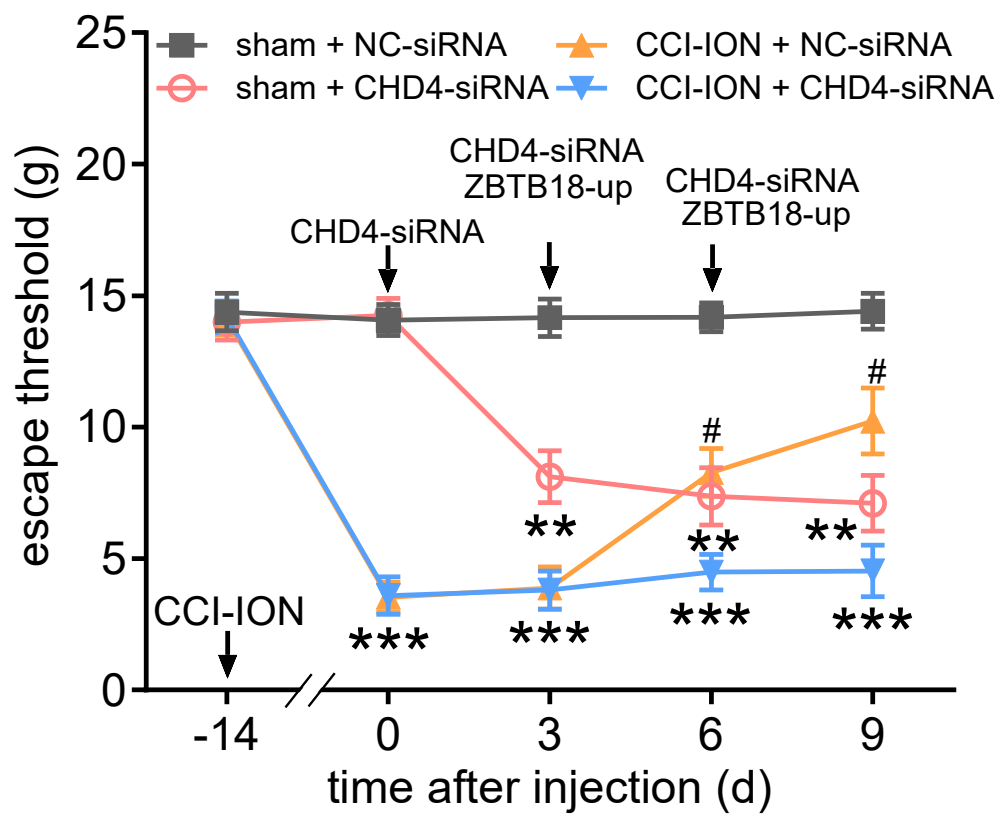

Fig. S21. CHD4-siRNA pretreatment induced mechanical allodynia in intact rats and abolished ZBTB18-up-mediated alleviation of mechanical allodynia in CCI-ION rats. **$p < 0.01$, ***$p < 0.001$ compared with corresponding sham + NC-siRNA group; $^{\#}p < 0.05$, compared with corresponding CCI-ION + CHD4-siRNA group, two-way ANOVA, n = 6 rats/group.

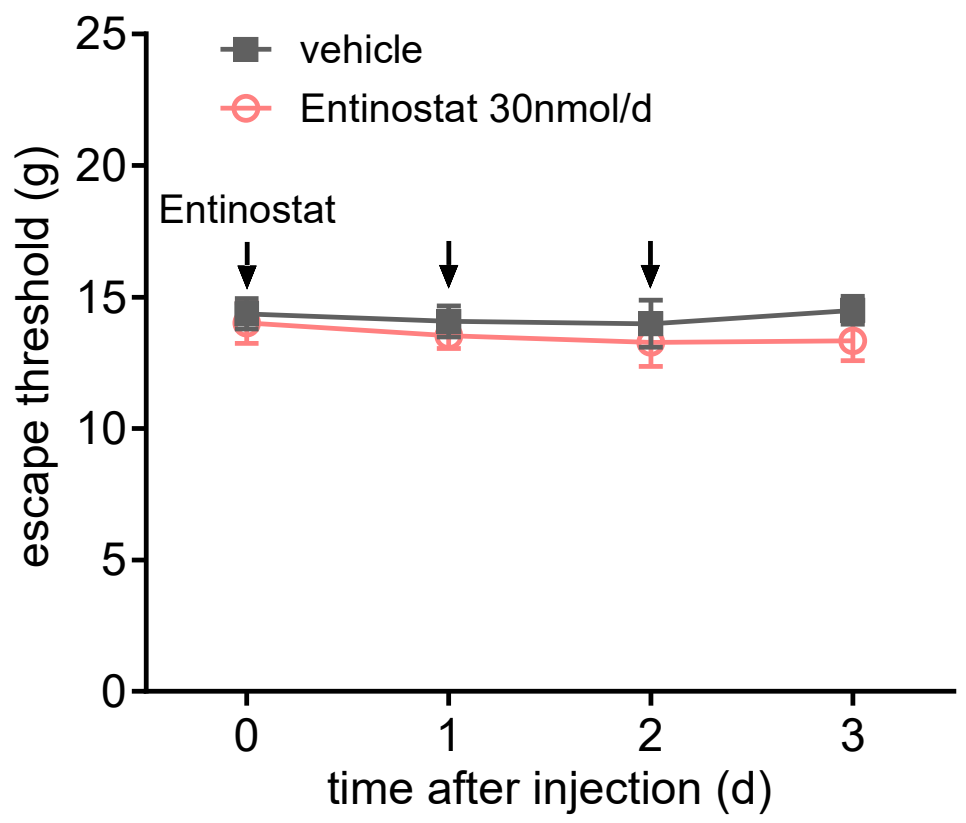

Fig. S22. Intra-TG injection of the class I HDAC inhibitor entinostat had no effect on basal mechanical thresholds in intact rats. $p > 0.05$, compared with corresponding vehicle group, two-way ANOVA, n = 6 rats/group.

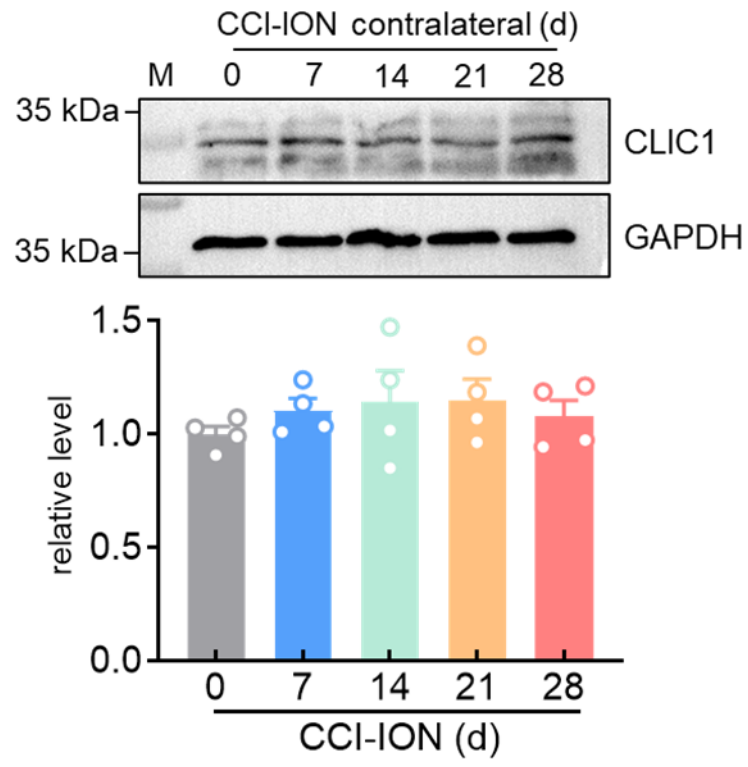

Fig. S23. Quantitative analysis shows that CCI-ION surgery did not significantly alter CLIC1 protein levels in the contralateral TGs across the indicated time points $p > 0.05$, compared with day 0 group, one-way ANOVA, $n = 4$ rats/time point/group. Shown blots are representative of four independent experiments.

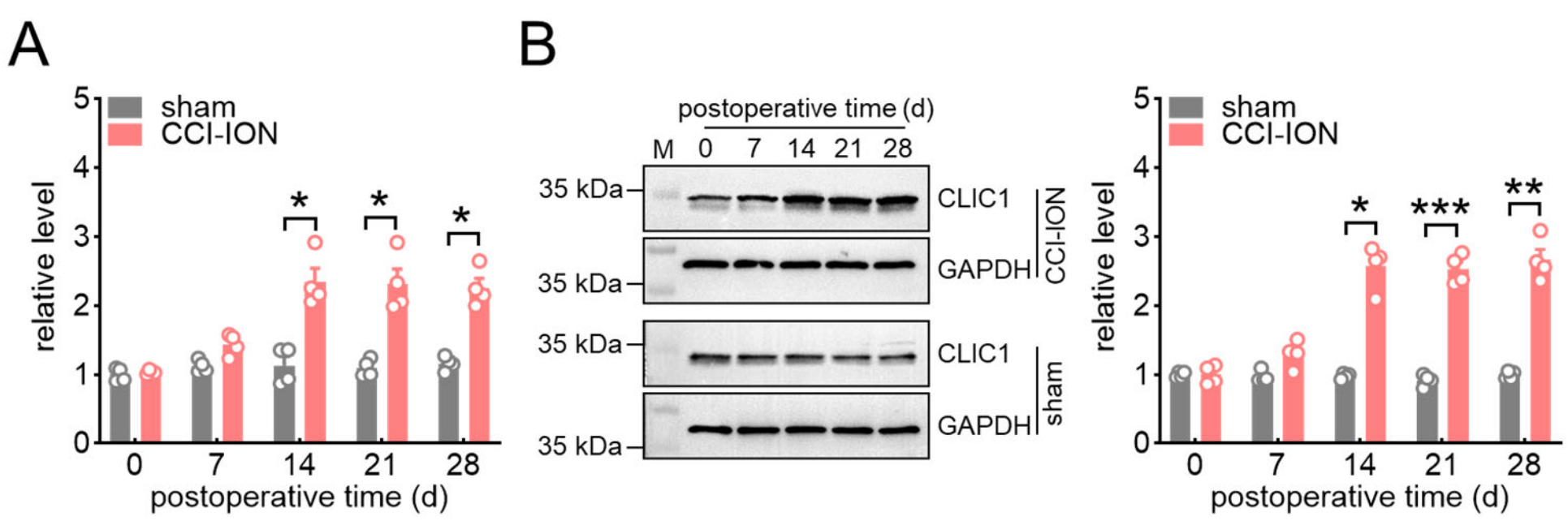

Fig. S24. mRNA and protein expression of CLIC1 in TGs after CCI-ION in female rats. *(A)* Time-dependent changes in *Clic1* mRNA expression after CCI-ION in female rats. $*p < 0.05$, compared with corresponding sham group, two-way ANOVA, $n = 4$ rats/time point/group. *(B)* Immunoblot analysis of CLIC1 protein expression over time. $*p < 0.05$, $**p < 0.01$, $***p < 0.001$ compared with corresponding sham group, two-way ANOVA, $n = 4$ rats/time point/group. Shown blots are representative of four independent experiments.

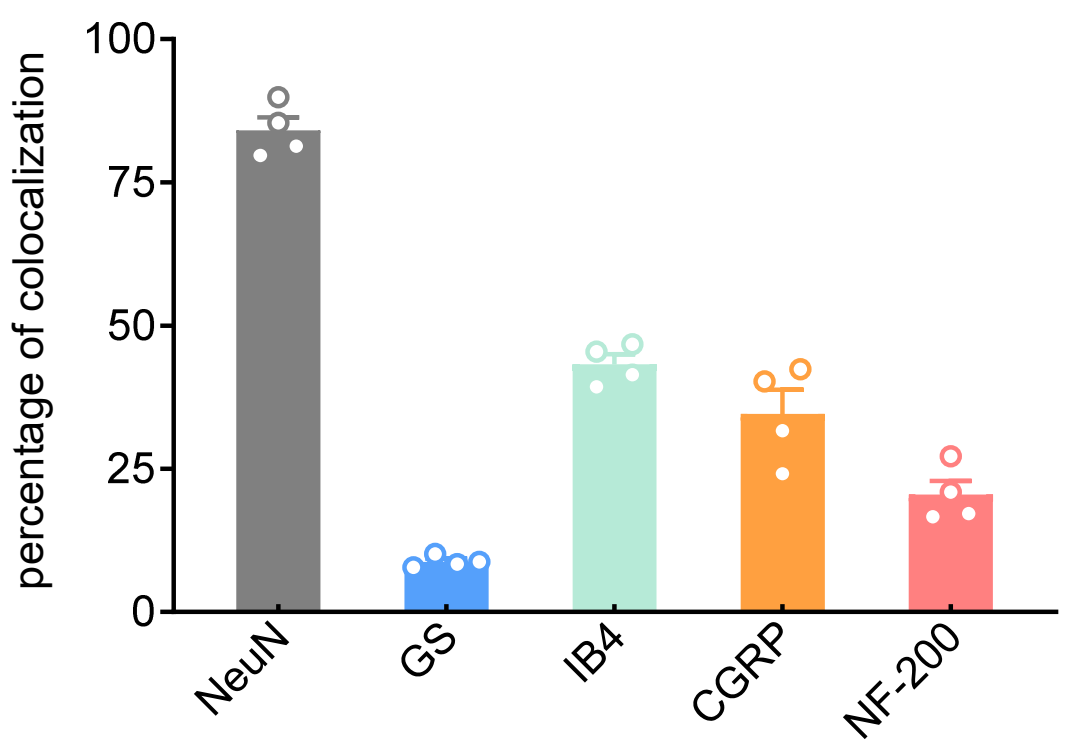

Fig. S25. Quantification of CLIC1-positive neurons coexpressing specific markers. n = 4 biological replicates.

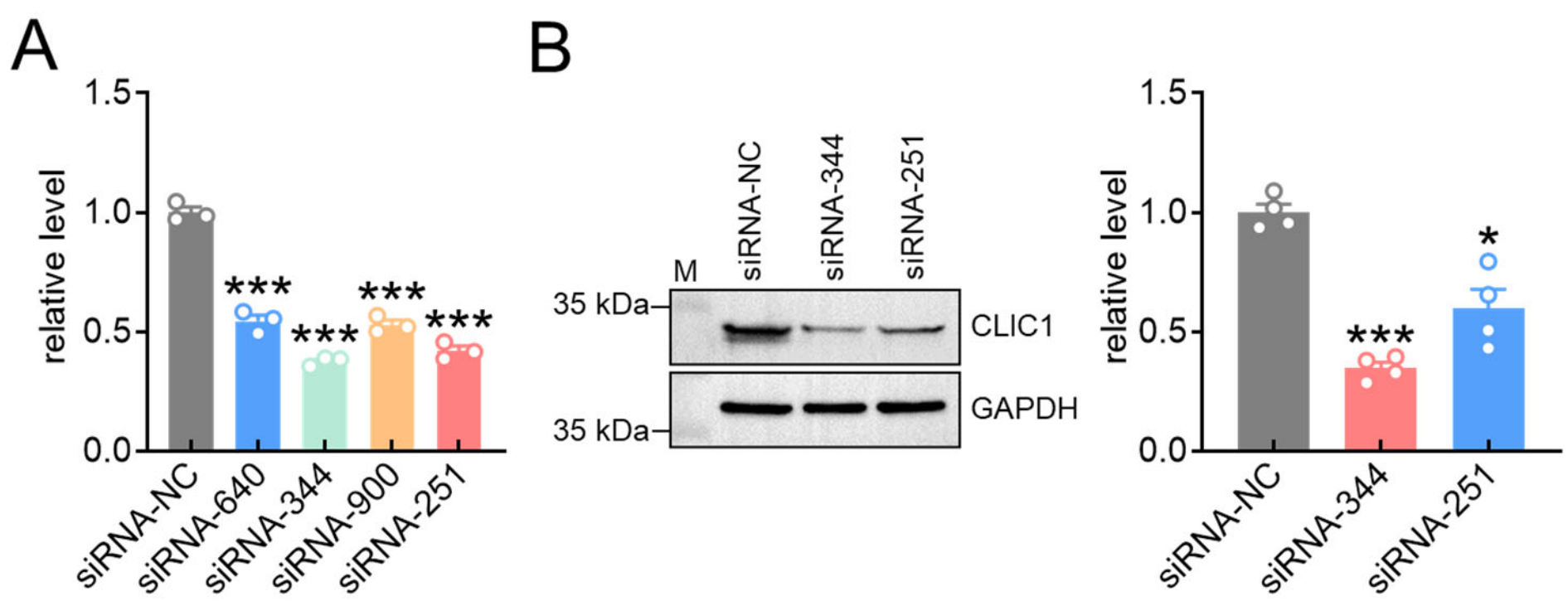

Fig. S26. Screening of siRNAs targeting CLIC1 in rats. *(A)* CLIC1 mRNA levels in PC12 cells 48 h after transfection with four independent siRNAs (siRNA-640, siRNA-344, siRNA-900 and siRNA-251). ***$p < 0.001$ compared with siRNA-NC group, one-way ANOVA, $n = 3$. *(B)* CLIC1 protein levels after intra-TG delivery of modified siRNA-344 or siRNA-251. *$p < 0.05$, ***$p < 0.001$ compared with siRNA-NC group, one-way ANOVA, $n = 4$ rats/group. Shown blots are representative of four independent experiments.

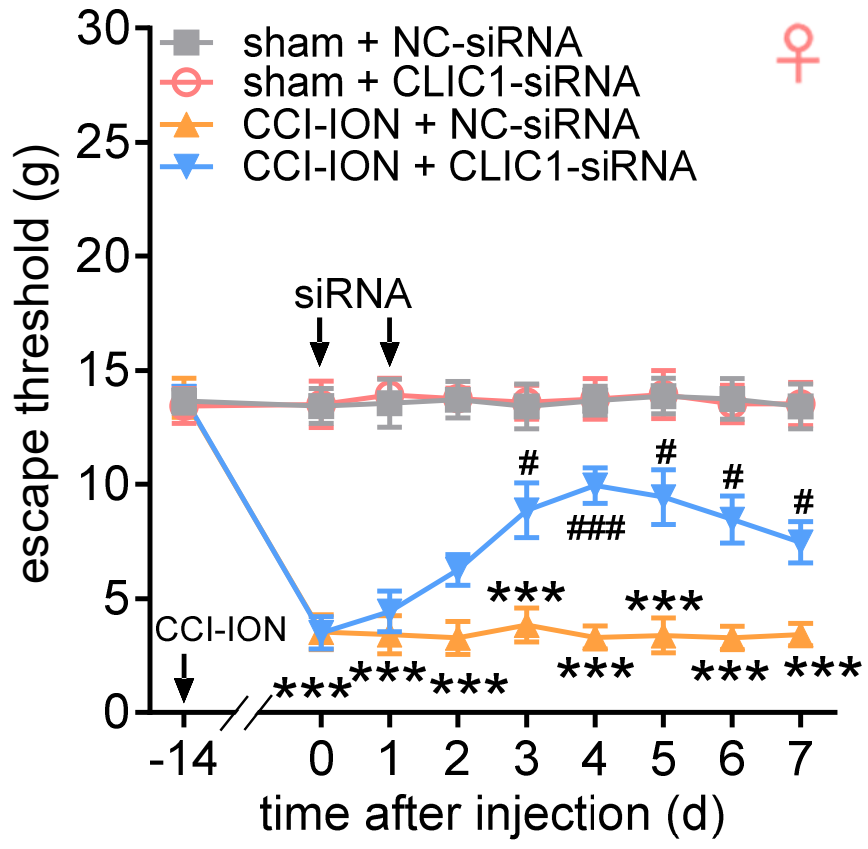

Fig. S27. Intra-TG injection of CLIC1-siRNA ameliorated mechanical allodynia in female CCI-ION rats. ***$p < 0.001$ compared with corresponding sham + NC-siRNA group; $^{\#}p < 0.05$, $^{\#\#\#}p < 0.001$ compared with corresponding CCI-ION + NC-siRNA group, two-way ANOVA, $n = 6$ rats/group.

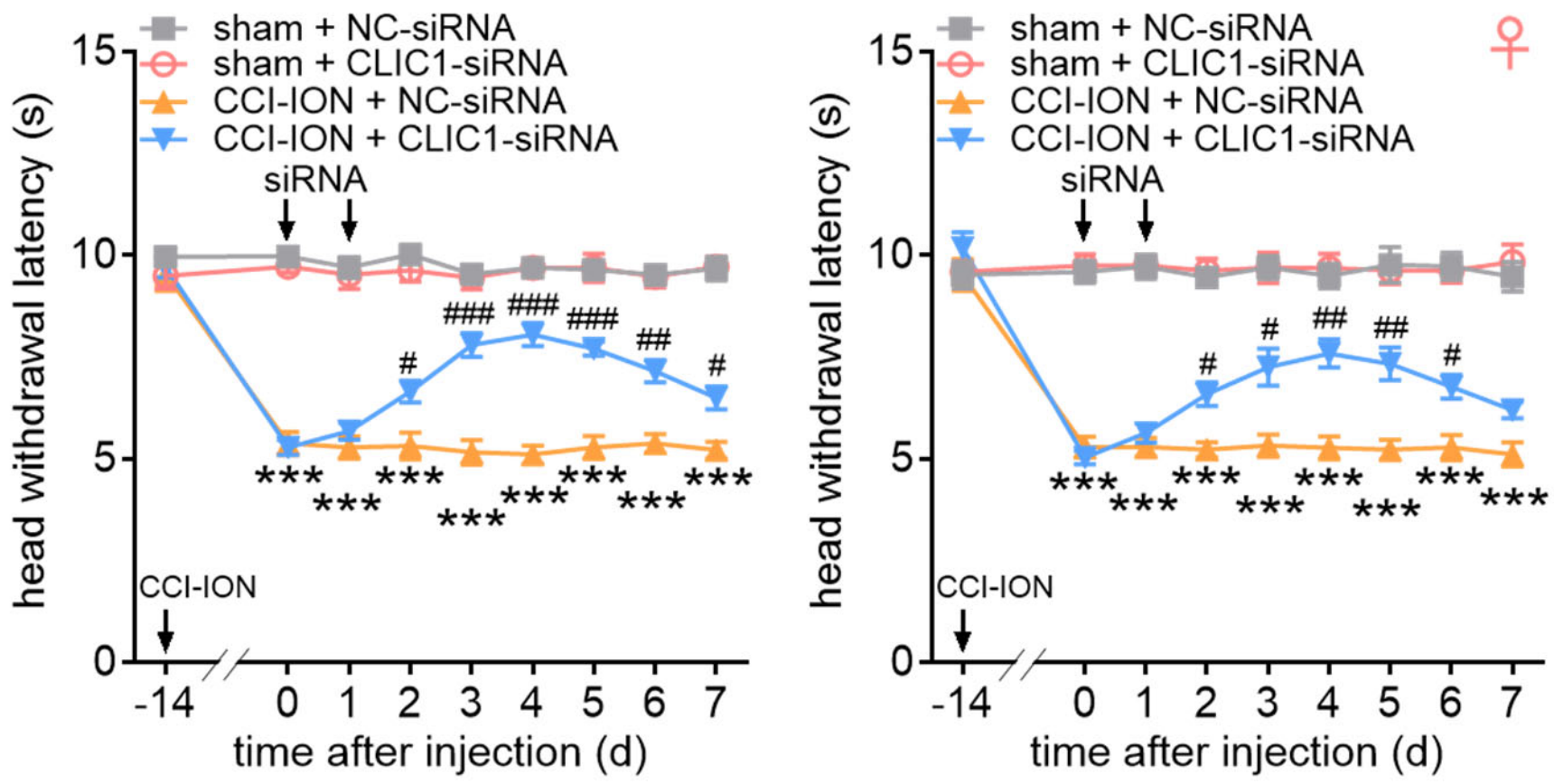

Fig. S28. Intra-TG injection of CLIC1-siRNA ameliorated thermal hyperalgesia in male (left) and female (right) CCI-ION rats. ***$p < 0.001$ compared with corresponding sham + NC-siRNA group; $^{\#}p < 0.05$, $^{\#\#}p < 0.01$, $^{\#\#\#}p < 0.001$ compared with corresponding CCI-ION + NC-siRNA group, two-way ANOVA, $n = 6$ rats/group.

SI Tables S1 to S2

Table S1: siRNAs used in the current study.

Name	Sense	Antisense
ZBTB18-Rat-1065	GGAACAUGUGGAUGCGAUUTT	AAUCGCAUCCACAUGUUCCTT
ZBTB18-Rat-647	GCAGCUCAACAGUCUUUTT	AAAGACUCUGUUGAGCUGCTT
ZBTB18-Rat-400	GCUUCAAGUUGUUCGGAUATT	UAUCCGAACAACUUGAAGCTT
ZBTB18-Rat-736	CAGCCUUUCAGGAGUUGAATT	UUCAACUCCUGAAAGGCUGTT
CHD4-Rat-1	GCAAUUAUGCGAUAUGGUATT	UACCAUAUCGCAUAAUUGCTT
CHD4-Rat-2	CACUCGAAAUUUUGAAGCATT	UGCUUCAAAAUUUCGAGUGTT
CHD4-Rat-3	CCAAGGACCUGAAUGAUGATT	UCAUCAUUCAGGUCCUUGGTT
CHD4-Rat-4	CUAUCGCUAUGGAAUAAAATT	UUUUAUUCCAUAGCGAUAGTT
CLIC1-Rat-344	GGAGUCACCUUCAACGUUATT	UAACGUUGAAGGUGACUCCTT
CLIC1-Rat-640	CCUGAAGGUUCUAGACAAUTT	AUUGUCUAGAACCUUCAGGTT
CLIC1-Rat-900	GUCCUGACGAUGAAGAGAUTT	AUCUCUUCAUCGUCAGGACTT
CLIC1-Rat-251	CAGGUCGAAUUGUUCGUGATT	UCACGAACAAUUCGACCUGTT
NC-siRNA	UUCUCCGAACGUGUCACGUTT	ACGUGACACGUUCGGAGAATT

Table S2: Sequences of nucleotides used in the current study.

Name	Forward	Reverse
Gapdh	GTGCTGAGTATGTCGTGGAGT	CAGTCTTCTGAGTGGCAGTGAT
Zbtb42	TCTCAGAGGAGTCGAGTGGG	AACGAATCTTGCTCGGCCTT
Zbtb38	TGGGCGTGAAGGAGTTCATC	CCGCTCGTGGTTCCTTTTTG
Zbtb44	ATGTCCAACCTGTGGGGTTC	AGGCGATGCATCTTTAGCGA
Zbtb6	CAGAGCGAAGGGGAGTGAAG	TCACTGAATGTTGTGTTTCAGGG
Zbtb47	GCCCCAATATGAAACGGCAC	AGCATGTTGGAGAAGCGGAA
Zbtb11	CATTGCACTCAATGTGAGAAAAGT	CAAAACTGGCACTGGAACGG
Zbtb48	ATCAAGGCCAAGCACAGGAA	GTCCAGACTGGCTTGGGTG
Zbtb18	TGATGAAGACGAAGGCGAAG	GGGCTGGCTACTGTTTTTCC
Zbtb16	AGAGGACCGTAAGGCTCGAT	CTGCTGCCTTGGTAGGACTC
ZBTB18-F1 methylation	ATAAGTATTAATTTTATTGGAAAGGTT	AAAACCTACTTCCAAACTAAAAACT
ZBTB18-F2 methylation	GTTAGTTTTTAGTTTGGAAGTAGGTT	ACRACRCRACCCAAATATAACCC
ZBTB18-F3 methylation	TYGGGYGGGGTTATATTTGG	CCTCCTCCTCTACCRCCRC
Zbtb18-F1	ACATCTTTTTCCTTTTATCCTCT	GATCTGCTAGTCTCTGTCTCTCA
Zbtb18-F2	TTCATCTAAATACTGGTAGCCCG	AGTCTTCAGATTCAACCCGAATC
Zbtb18-F3	AAAACCACTTGTAAACGCCCCTA	CAAACACAAAAACACAGTATCCC
Zbtb18-F4	TTTCCAACAGAATATCAATATCAGT	CAAAGGAGTAAAGAAGTTGAAAGTA
Zbtb18-F5	ATTCAATGTCTGATTTGCCCAC	AAAAAGTTGAGTAACAAGCGGG
Zbtb18-F6	GTGTGTTGAGTTCCTCGCTATG	CCCTTCAGATAAACTTCCTCCC
Zbtb18-F7	CCTTCCATTATATCCAGAGCAGAA	TGAGATTGATACTTGTAGACGCTT
Zbtb18-F8	CCTTCCTTACAAAGCCGTGG	TGCGAGCTGCAAACTCCTAG
Zbtb18-F9	CCTCTCACAGCCGCCTAG	GTGTGGAGCGAGCGAGTC
Zbtb18-F10	CTTATGGCCGTCGCGGTGCA	CTGTTGGGTGGGCGTGTTTT
Zbtb18-F11	GAAAAAACACGCCCACCC	TCAGCGATTGAGCGAAAG
Zbtb18-F12	CGCTTTCGCTCAATCGC	CCTGCCAGACGCACGGG

site a	GACAGGCTTTCAGAGGAC	AGTGAGGTTAGGAATACGG
site b	CGGTCGGTGAGTCAGC	GGCGCAACCTAAGTCC
Clic1-TSS	GACCCACTCCCACGATCA	GCTCAGTGCACCCCACA
Reg3b	AACCCAATGGAGGTGGATGG	GACAAGCTGCCACAGAATCC
Socs3	CTTCAGCTCCAAGAGCGAGT	TAGGTTCTTGGTCCCCGACT
Ngf	TTTTGATCGGCGTACAGGCA	GAGGGCTGTGTCAAGGGAAT
Crlf1	TTAGCTTCCGCCTGTACGAC	GGATCCCCTCGTCCTGGTTT
Fos	TACTACCATTCCCCAGCCGA	GCTGTCACCGTGGGGATAAA
Crabp2	TGAGGAAGATTGCTGTGGCT	GCTCCTCAAATTCCTCCCCG
Ncf1	GATGTTCCCCATTGAGGCCG	TCTGGCTTCTTCACCTGGC
Hmgb2	TCCCAAGAGACCACCGTCTG	TTTTGCCCTTGGCACGGTAT
Lgals3	CTTCCACTTTAACCCCCGCT	TGAATGGTTTGCCGCTCTCA
CLIC1	AACCCCGAATCCAACACCTC	GGAGTCCCTTCTCTAGGTTGTC
CLIC2	CTCAACACCCAAGCAGACCC	CTTCAGTTCTTCAGGTTTTCTGGC
CLIC3	AGCTCTTTGTGAAGGCCAGTGA	ATTGTGATCCCGGTGCGAAG
CLIC4	CTCCCCTCCCTGACGAAATC	TGGGAATGTCAAAGTTGCGG
CLIC5	AAAGCCAGCCGATCTACACAA	CCAGTTTGGGGTACTTCTCAGG
CLIC6	GAAGAGTTGGGGCGAGTGAA	CATCAGAGCCAGCCTTGACA

SI Data S1 to S3

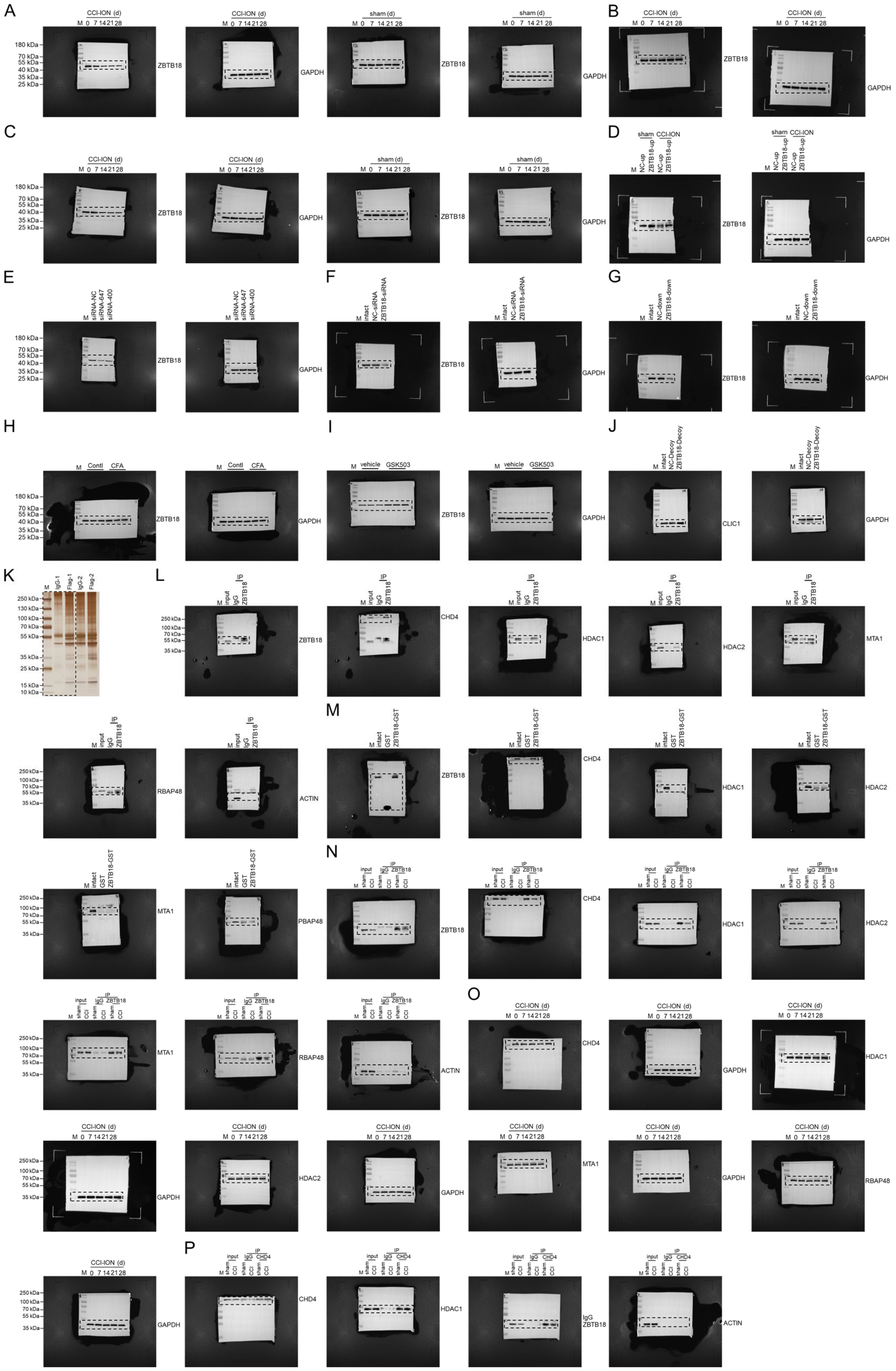

Data S1: Unedited blot images in this study.

(A) Unedited blots for Fig. 1G.

(B) Unedited blots for Fig. *S1*.

(C) Unedited blots for Fig. *S2B*.

(D) Unedited blots for Fig. *S9*.

(E) Unedited blots for Fig. *S10B*.

(F) Unedited blots for Fig. *S10C*.

(G) Unedited blots for Fig. *S10D*.

(H) Unedited blots for Fig. 2K.

(I) Unedited blots for Fig. *S7*.

(J) Unedited blots for Fig. 3K.

(K) Unedited blots for Fig. 4A.

(L) Unedited blots for Fig. 4C.

(M) Unedited blots for Figs. 4D and *S15*.

(N) Unedited blots for Fig. 4E.

(O) Unedited blots for Fig. *S16*.

(P) Unedited blots for Fig. 4F.

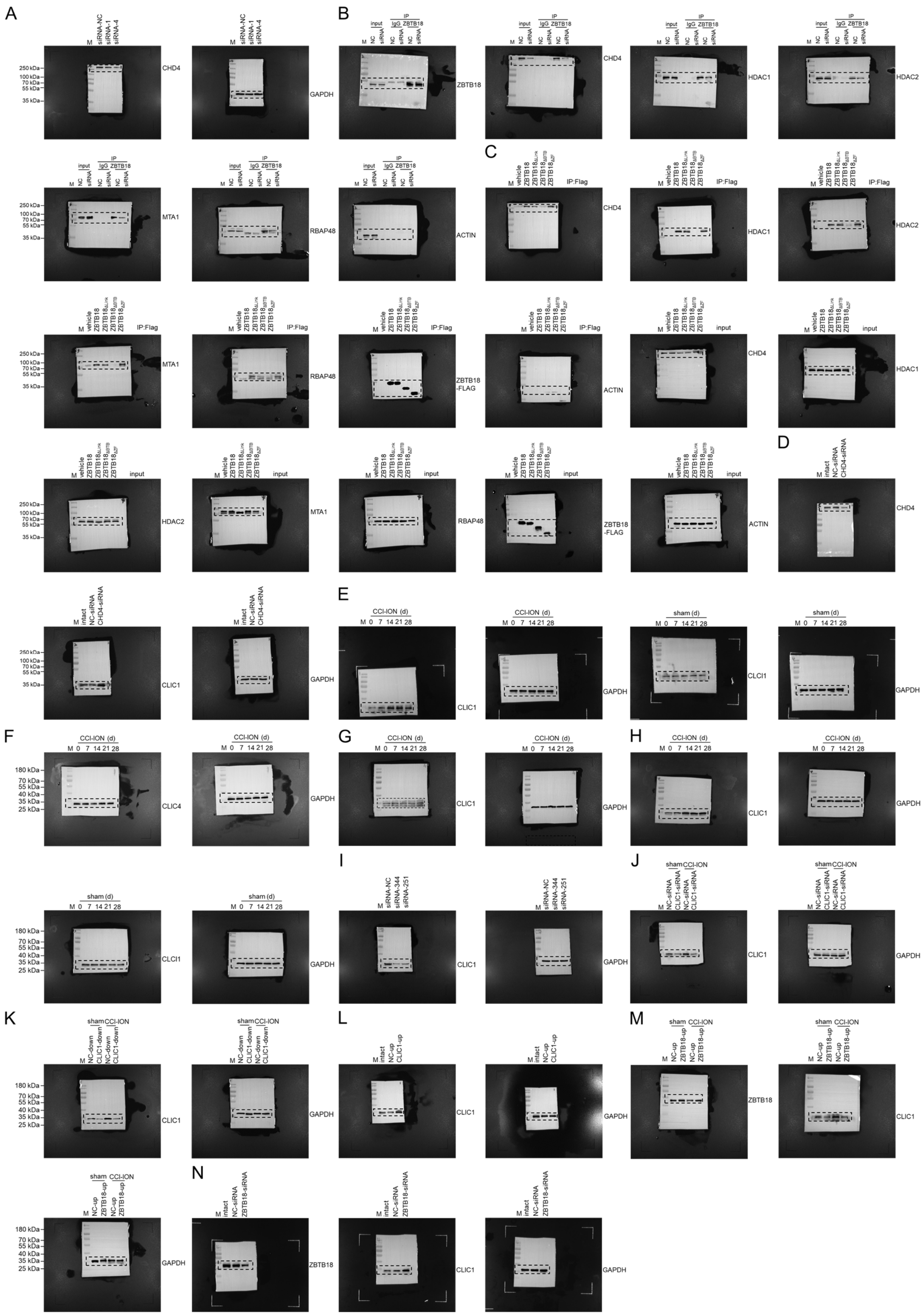

A
M
siRNA-NC
siRNA-1
siRNA-4
250 kDa
100 kDa
70 kDa
55 kDa
35 kDa
CHD4
GAPDH
B
input
IP
IgG
ZBTB18
NC
siRNA
ZBTB18
CHD4
HDAC1
HDAC2
MTA1
RBAP48
ACTIN
C
vehicle
IP:Flag
input
ZBTB18-FLAG
D
intact
NC-siRNA
CHD4-siRNA
CLIC1
E
CCI-ION (d)
sham (d)
M 0 7 14 21 28
F
180 kDa
40 kDa
25 kDa
CLIC4
G
H
I
siRNA-344
siRNA-251
J
sham
CCI-ION
CLIC1-siRNA
K
NC-down
CLIC1-down
L
NC-up
CLIC1-up
M
ZBTB18-up
N
ZBTB18-siRNA

Data S2: Unedited blot images in this study.

(A) Unedited blots for Fig. *S17B*.

(B) Unedited blots for Fig. 4G.

(C) Unedited blots for Fig. 4J.

(D) Unedited blots for Fig. 4O.

(E) Unedited blots for Fig. 5C.

(F) Unedited blots for Fig. 5D.

(G) Unedited blots for Fig. *S23*.

(H) Unedited blots for Fig. *S24B*.

(I) Unedited blots for Fig. *S26B*.

(J) Unedited blots for Fig. 5G.

(K) Unedited blots for Fig. 5N.

(L) Unedited blots for Fig. 5Q.

(M) Unedited blots for Fig. 6A.

(N) Unedited blots for Fig. 6G.

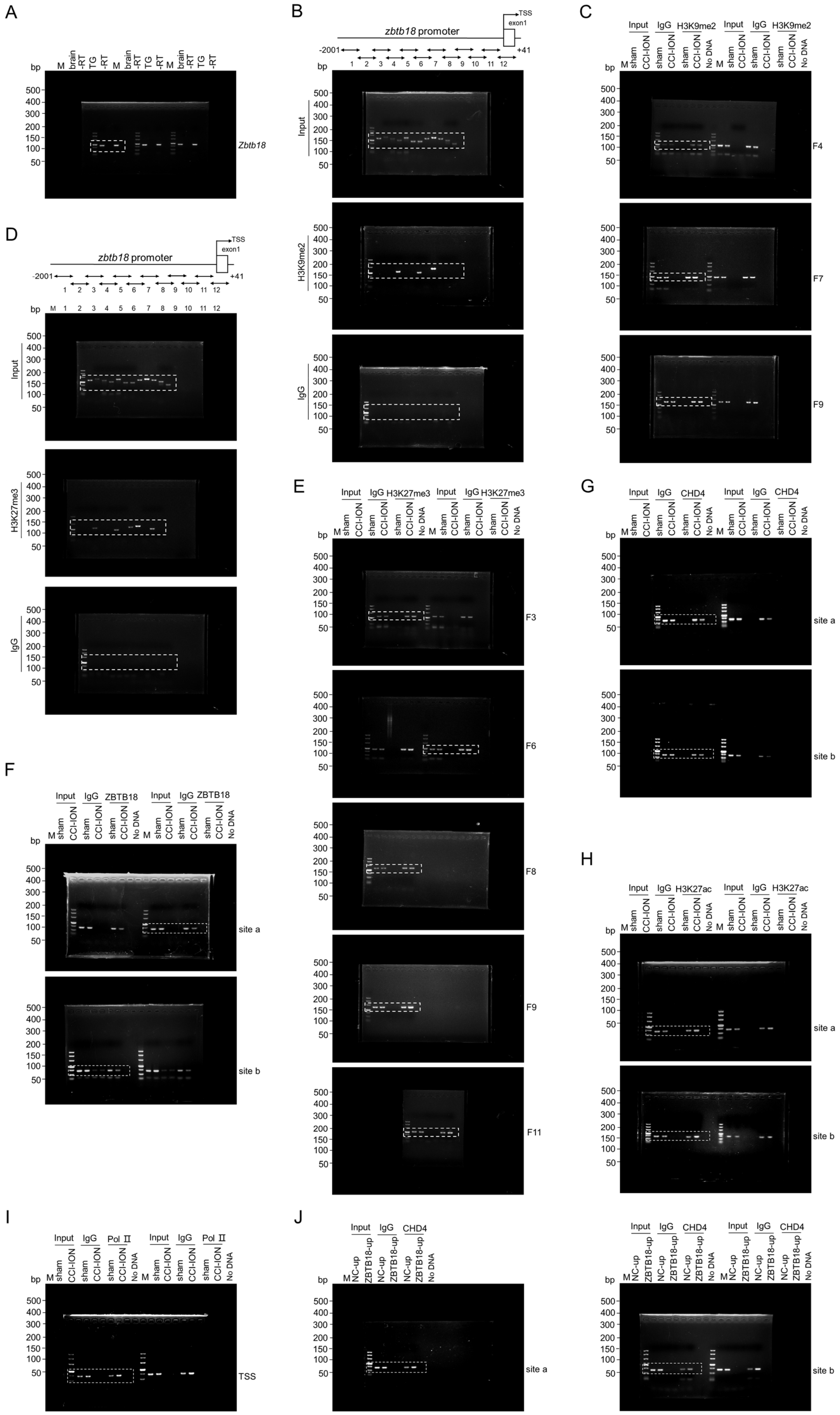
A
B
C
D
E
F
G
H
I
J
bp
M
brain
-RT
TG
Zbtb18
zbtb18 promoter
TSS
exon1
-2001
+41
1
2
3
4
5
6
7
8
9
10
11
12
500
400
300
200
150
100
50
Input
H3K9me2
IgG
H3K27me3
ZBTB18
CHD4
H3K27ac
Pol II
sham
CCI-ION
No DNA
NC-up
ZBTB18-up
F3
F4
F6
F7
F8
F9
F11
site a
site b
TSS

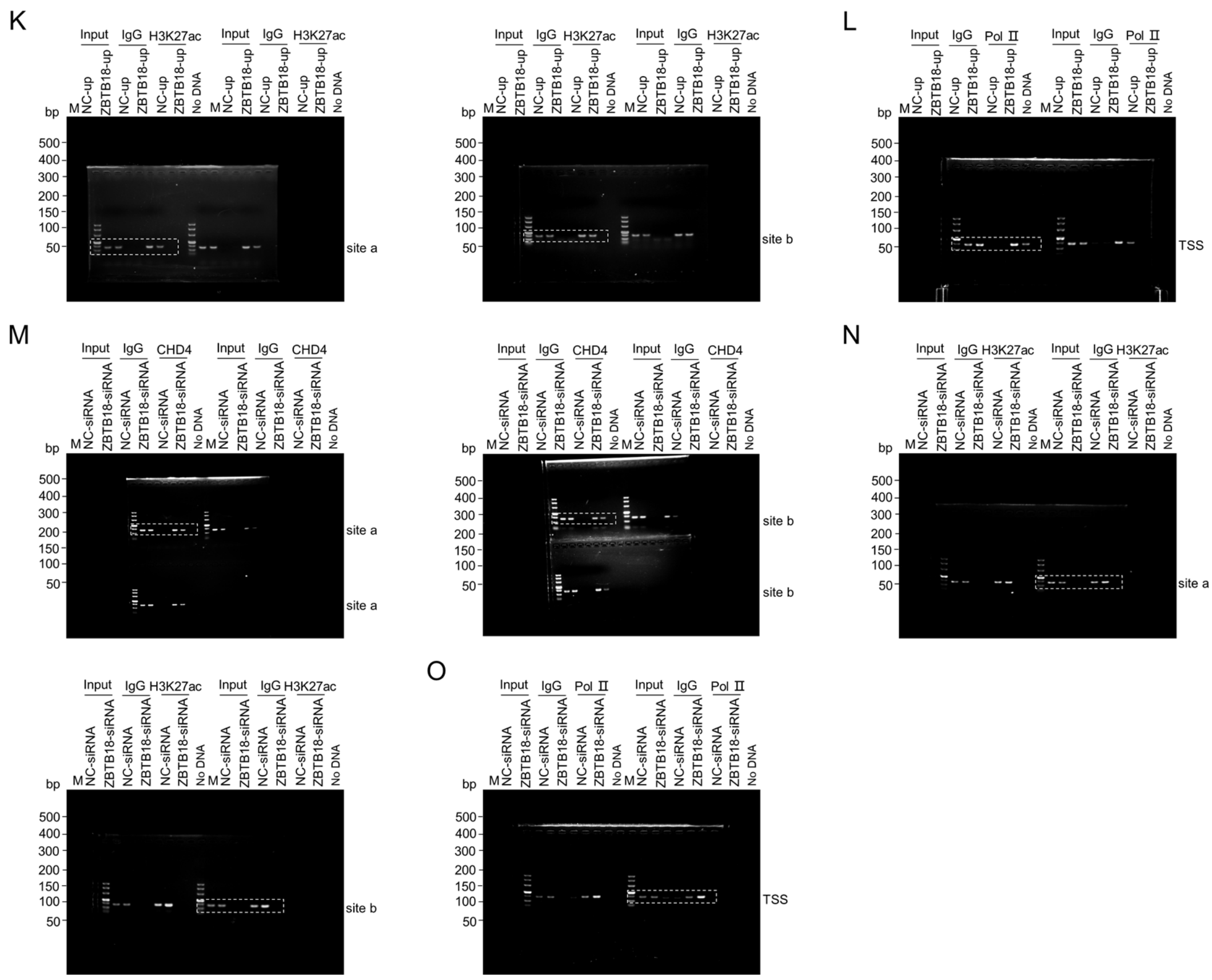

Data S3: Unedited gel images in this study.

(A) Unedited gels for Fig. 1D.

(B) Unedited gels for Fig. *S5A*.

(C) Unedited gels for Fig. *S5B*.

(D) Unedited gels for Fig. *S6A*.

(E) Unedited gels for Fig. *S6B*.

(F) Unedited gels for Fig. 3I.

(G) Unedited gels for Fig. 4L.

(H) Unedited gels for Fig. 4M.

(I) Unedited gels for Fig. 4N.

(J) Unedited gels for Fig. *S19A*.

(K) Unedited gels for Fig. *S19B*.

(L) Unedited gels for Fig. *S19C*.

(M) Unedited gels for Fig. *S20A*.

(N) Unedited gels for Fig. *S20B*.

(O) Unedited gels for Fig. *S20C*.
